# Comprehensive volumetric phenotyping of the neonatal brain in Down syndrome

**DOI:** 10.1093/cercor/bhad171

**Published:** 2023-05-26

**Authors:** Abi Fukami-Gartner, Ana A Baburamani, Ralica Dimitrova, Prachi A Patkee, Olatz Ojinaga-Alfageme, Alexandra F Bonthrone, Daniel Cromb, Alena U Uus, Serena J Counsell, Joseph V Hajnal, Jonathan O’Muircheartaigh, Mary A Rutherford

**Affiliations:** Centre for the Developing Brain, School of Biomedical Engineering and Imaging Sciences, King’s College London, St. Thomas’ Hospital, London SE1 7EH, United Kingdom; MRC Centre for Neurodevelopmental Disorders, Institute of Psychiatry, Psychology and Neuroscience, King’s College London, London SE1 1UL, United Kingdom; Centre for the Developing Brain, School of Biomedical Engineering and Imaging Sciences, King’s College London, St. Thomas’ Hospital, London SE1 7EH, United Kingdom; Centre for the Developing Brain, School of Biomedical Engineering and Imaging Sciences, King’s College London, St. Thomas’ Hospital, London SE1 7EH, United Kingdom; Department of Forensic and Neurodevelopmental Science, Institute of Psychiatry, Psychology and Neuroscience, King’s College London, London SE5 8AF, United Kingdom; Centre for the Developing Brain, School of Biomedical Engineering and Imaging Sciences, King’s College London, St. Thomas’ Hospital, London SE1 7EH, United Kingdom; Centre for the Developing Brain, School of Biomedical Engineering and Imaging Sciences, King’s College London, St. Thomas’ Hospital, London SE1 7EH, United Kingdom; Centre for Brain and Cognitive Development, Birkbeck, University of London, London WC1E 7HX, United Kingdom; Centre for the Developing Brain, School of Biomedical Engineering and Imaging Sciences, King’s College London, St. Thomas’ Hospital, London SE1 7EH, United Kingdom; Centre for the Developing Brain, School of Biomedical Engineering and Imaging Sciences, King’s College London, St. Thomas’ Hospital, London SE1 7EH, United Kingdom; Centre for the Developing Brain, School of Biomedical Engineering and Imaging Sciences, King’s College London, St. Thomas’ Hospital, London SE1 7EH, United Kingdom; Department of Biomedical Engineering, School of Biomedical Engineering and Imaging Sciences, King’s College London, London SE1 7EH, United Kingdom; Centre for the Developing Brain, School of Biomedical Engineering and Imaging Sciences, King’s College London, St. Thomas’ Hospital, London SE1 7EH, United Kingdom; Centre for the Developing Brain, School of Biomedical Engineering and Imaging Sciences, King’s College London, St. Thomas’ Hospital, London SE1 7EH, United Kingdom; Department of Biomedical Engineering, School of Biomedical Engineering and Imaging Sciences, King’s College London, London SE1 7EH, United Kingdom; Centre for the Developing Brain, School of Biomedical Engineering and Imaging Sciences, King’s College London, St. Thomas’ Hospital, London SE1 7EH, United Kingdom; MRC Centre for Neurodevelopmental Disorders, Institute of Psychiatry, Psychology and Neuroscience, King’s College London, London SE1 1UL, United Kingdom; Department of Forensic and Neurodevelopmental Science, Institute of Psychiatry, Psychology and Neuroscience, King’s College London, London SE5 8AF, United Kingdom; Centre for the Developing Brain, School of Biomedical Engineering and Imaging Sciences, King’s College London, St. Thomas’ Hospital, London SE1 7EH, United Kingdom; MRC Centre for Neurodevelopmental Disorders, Institute of Psychiatry, Psychology and Neuroscience, King’s College London, London SE1 1UL, United Kingdom

**Keywords:** Down syndrome, magnetic resonance imaging, neonate, normative modeling, volumetric

## Abstract

Down syndrome (DS) is the most common genetic cause of intellectual disability with a wide range of neurodevelopmental outcomes. To date, there have been very few *in vivo* neuroimaging studies of the neonatal brain in DS. In this study we used a cross-sectional sample of 493 preterm- to term-born control neonates from the *developing Human Connectome Project* to perform normative modeling of regional brain tissue volumes from 32 to 46 weeks postmenstrual age, accounting for sex and age variables. Deviation from the normative mean was quantified in 25 neonates with DS with postnatally confirmed karyotypes from the *Early Brain Imaging in DS* study. Here, we provide the first comprehensive volumetric phenotyping of the neonatal brain in DS, which is characterized by significantly reduced whole brain, cerebral white matter, and cerebellar volumes; reduced relative frontal and occipital lobar volumes, in contrast with enlarged relative temporal and parietal lobar volumes; enlarged relative deep gray matter volume (particularly the lentiform nuclei); and enlargement of the lateral ventricles, amongst other features. In future, the ability to assess phenotypic severity at the neonatal stage may help guide early interventions and, ultimately, help improve neurodevelopmental outcomes in children with DS.

## Introduction

Down syndrome (DS) is the most common viable chromosomal abnormality affecting an estimated 1 in 700 ~ 1000 live births worldwide annually (De Graaf et al. 2017; de Graaf et al. 2021). It is caused by the partial or complete triplication of *Homo sapiens* chromosome 21 (*Hsa21*), which can occur as a result of three different mechanisms: free trisomy 21 (i.e. a free supernumerary *Hsa21* in all cells, also known as nondisjunction, in ~ 95% of cases), translocation (i.e. translocation of all or part of *Hsa21* onto another chromosome, in ~ 3% of cases), or mosaicism (i.e. only a proportion of cells have trisomy 21, in ~ 2% of cases) (Devlin 2004; Devlin and Morrison 2004; Shin et al. 2010; Morris et al. 2012).

DS is a complex genomic disorder, which affects both physical and cognitive development to produce a well-recognized syndromic phenotype that includes characteristic craniofacial and musculoskeletal features, increased risk for a number of health issues, and a distinctive cognitive and behavioral phenotype, although the severity of specific physical and cognitive impairments vary considerably between affected individuals (Silverman 2007; Karmiloff-Smith et al. 2016).

From a cognitive perspective, DS is the most common cause of intellectual disability with a known genetic etiology. The majority of individuals are classified as having mild to moderate disability, although a wide and largely unexplained range of neurodevelopmental outcomes are observed. The cognitive phenotype of individuals with DS demonstrates strengths in visual learning, but weaknesses in expressive language, verbal working memory, and episodic memory (Chapman and Hesketh 2000; Silverman 2007; Antonarakis et al. 2020).

From a physical perspective, individuals with DS are generally of short stature with characteristic craniofacial features, including a brachycephalic skull with a flat occiput (Starbuck et al. 2017; Rodrigues et al. 2019). Clinical comorbidities commonly observed in neonates, include congenital heart defects (CHDs), gastrointestinal malformations (e.g. duodenal atresia, Hirschsprung’s disease), haematological disorders (e.g. thrombocytopenia, polycythaemia), endocrine issues (e.g. hypothyroidism), and hearing and visual impairments amongst others (Stoll et al. 2015; Startin et al. 2020).

CHDs are present in approximately 50% of neonates with DS, the most common of which are atrioventricular septal defects (AVSD, in ~ 42% of CHD cases in DS), ventricular septal defects (VSD, ~ 22%) and atrial septal defects (ASD, ~ 16%), whereas other cardiac defects are also noted in smaller numbers (Bergström et al. 2016; Versacci et al. 2018). Previous studies have shown that children with DS and an associated AVSD had poorer neurodevelopmental outcomes in multiple areas of assessment, including fine motor skills, as well as expressive and receptive language, compared with children with DS with a structurally normal heart (Visootsak et al. 2011, 2013, 2016).

Magnetic resonance imaging (MRI) has been used to investigate differences in whole and/or regional brain volumes in DS from infancy (Gunbey et al. 2017), early childhood (Kates et al. 2002; Kaufmann et al. 2003; Śmigielska-Kuzia et al. 2011), middle childhood (Pinter et al. 2001a, 2001b; Carter et al. 2008; Carducci et al. 2013), adolescence, to young adulthood (Jernigan and Bellugi 1990; Jernigan et al. 1993; Menghini et al. 2011; Lee et al. 2016, 2020; Levman et al. 2019; McCann et al. 2021) (see Hamner et al. 2018 for a review of pediatric neuroimaging in DS). However, there is still a gap in knowledge about structural brain development in DS at the very earliest timepoints (i.e. fetal and neonatal) and how this may be associated with later neurodevelopmental outcomes. It is currently not possible to predict the severity of later neurodevelopmental outcomes at an early stage (e.g. antenatally or in early postnatal life).

To date, there have been very few *in vivo* fetal or neonatal neuroimaging studies in DS, despite the presence of clearly identifiable structural and morphological differences *in utero* and at birth respectively (Patkee et al. 2020; Tarui et al. 2020; Yun et al. 2021). Previous volumetric studies have shown that whole brain and cerebellar volumes were smaller than age-matched euploid controls from the second trimester (< 28 weeks gestational age, GA), whereas total cortical gray matter (GM) volume was reduced from the third trimester onwards (> 28 weeks GA) (Patkee et al. 2020; Tarui et al. 2020). However, to date, volumetric differences in regional cortical GM, regional white matter (WM), specific deep GM structures and other segments have not been examined in detail in neonates with DS. Furthermore, prior group-level analyses have not quantified individual variability within the highly heterogenous cohorts of participants with DS.

In this study, we aimed to conduct the first detailed regional volumetric analysis of the neonatal brain in DS. We used a robust cross-sectional sample of 493 preterm- to term-born control neonates from the *developing Human Connectome Project* (dHCP) to perform normative modeling of regional brain tissue volumes from 32 to 46 weeks postmenstrual age (PMA) at scan. Deviation from the normative mean was quantified in 25 neonates with DS from the *Early Brain Imaging in DS* (eBiDS) study, accounting for sex, age at scan, and age from birth variables.

## Materials and methods

### Ethical approval

Ethical approval was provided by London-based National Research Ethics Committees for the following studies: *quantification of fetal brain development using MRI* (trisomy 21 participant subgroup, henceforth denoted “T21 study”) [07/H0707/105], *Early Brain Imaging in DS* (eBiDS) [19/LO/0667] and the dHCP [14/LO/1169]. Informed written parental consent was obtained prior to MRI in all above studies, and prior to neurodevelopmental follow-up in the dHCP, in accordance with the declaration of Helsinki.

### Participants

#### Neonates with DS

Neonates with a postnatally confirmed karyotype for DS were recruited from the neonatal unit and postnatal wards at St Thomas’ Hospital London and invited for a neonatal scan up to < 46 weeks PMA at scan. Additionally, former fetal scan participants with a confirmed postnatal diagnosis of DS were invited for a neonatal scan if they had consented to be contacted post-delivery. A total of 36 neonates with DS were recruited to the T21 and eBiDS studies between 2014 and 2021. Ten neonatal scans were excluded from analysis due to the use of old acquisition protocols, which were not in line with the dHCP controls. One neonate was excluded from analysis due to an acute brain infarction/parenchymal hemorrhage. Therefore, data from 25 neonates with DS [12 female, 10 preterm births < 37 weeks GA, PMA at scan median (range) = 40.57 (32.43–45.57) weeks] (Table S1), scanned using the same acquisition parameters as dHCP controls, were used for analysis in this study.

#### Preterm- and term-born neonates for normative modeling

A total of 493 preterm- to term-born neonates [243 female, 86 preterm births < 37 weeks GA, of which 33 were born < 32 GA, PMA at scan median (range) = 41.00 (31.14–45.14) weeks] were selected from the dHCP (www.developingconnectome.org) (Edwards et al. 2022) for normative modeling of brain tissue volumes from 32 to 46 weeks PMA at scan. Exclusion criteria included incidental findings on MRI (Carney et al. 2021) (detailed in section *MR image review,* below) and Bayley III Scales of Infant Development (BSID-III) cognitive and motor composite scores (test mean [SD] = 100 [15]) below 70 (> 2 SD below the test mean) at 18 months (Michalec 2011). No repeat scans from the same neonate were used. Healthy neonates from twin pregnancies were included. Taking all the above exclusion criteria into account, this preterm- to term-born sample was used as a reference “control group” for the purposes of this specific study.

### Clinical information

Weight, head circumference (HC), and relevant clinical details were taken at the time of scan. Weights (at birth and scan, in kg) and HC (at birth and scan, in cm) were converted into *z*-scores based on the RCPCH UK-WHO growth charts (Wright et al. 2010) using the “*childsds*” package v0.7.6 in R (Table S1). For neonates with DS, CHD diagnosis (*n* = 13, 52% of DS group) and details of additional clinical comorbidities can be found in Supplementary Tables S2 and S3, respectively.

### MRI acquisition and pre-processing

Neonatal MRI data were acquired on a Philips Achieva 3 Tesla system using a dedicated 32-channel neonatal head coil and positioning system at the Evelina Newborn Imaging Centre, Evelina London Children’s Hospital (UK) as per (Hughes et al. 2017). Imaging was performed during natural sleep without sedation. *T*_2_-weighted scans were acquired with repetition time (TR) = 12,000 ms, echo time (TE) = 156 ms, flip angle = 90°, SENSE factor = 2.11/2.58 (axial/sagittal). The resultant in-plane resolution was 0.8 mm × 0.8 mm with a slice thickness of 1.6 mm and a slice overlap of 0.8 mm. Images were motion-corrected (Cordero-Grande et al. 2018) and super-resolution reconstructed (Kuklisova-Murgasova et al. 2012) resulting in a 0.5 mm^3^ isotropic pixel resolution.

### MR image review

All MRI scans were examined by a neonatal neuroradiologist. Exclusion criteria for the dHCP scans were incidental findings with possible or likely significance for clinical outcome and/or imaging analysis, including acute infarction or parenchymal hemorrhage, and major lesions within the WM, cortical GM, cerebellum, or basal ganglia. However, we did not exclude neonates with < 10 punctate white matter lesions (PWML), small subependymal cysts, small subdural hemorrhages, or hemorrhages in the caudothalamic notch, as these are common findings in low-risk neonates (Carney et al. 2021). In the DS group, one scan was excluded from analysis due to an acute brain infarction/parenchymal hemorrhage.

### MR image segmentation

Motion-corrected and reconstructed T_2_-weighted images (Kuklisova-Murgasova et al. 2012; Cordero-Grande et al. 2018) were corrected for bias-field inhomogeneities, brain extracted and segmented using the dHCP structural pipeline (https://github.com/BioMedIA/dhcp-structural-pipeline; Accessed 2021 November 15), an automated tissue structure segmentation algorithm optimized for the neonatal brain (Makropoulos et al. 2014, 2016, 2018). T_2_-weighted images were segmented into seven main tissue/fluid classes: extra-cerebral cerebrospinal fluid (eCSF), lateral ventricles, cortical GM, WM, deep GM, cerebellum, and brainstem (hereafter referred to as “main tissue classes”). In this program, the eCSF included the third and fourth ventricles but excluded the lateral ventricles. The lateral ventricles included the *cavum septum pellucidum*, a transient fluid-filled cavity located in the midline of the brain, between the left and right anterior horns, which if still present, typically closes in the neonatal period (Farruggia and Babcock 1981; Sundarakumar et al. 2015). Cortical GM, deep GM, and WM were further automatically segmented into the specific tissue segments listed in Table 1. Tissue segments for all DS and control scans were visually inspected for accuracy by the first author (A.F-G.), and where appropriate, any mislabelled voxels were manually corrected using ITK-SNAP (version 3.8.0) (Yushkevich et al. 2006). Tissue segments were used to extract absolute (in cm^3^) and relative (i.e. proportional) volumes. Relative volumes were calculated as the proportion of each tissue volume over total tissue volume (TTV), except for the lateral ventricles, which were calculated as a proportion of total brain volume (TBV), and eCSF as a proportion of intracranial volume (ICV) as defined in Table 1.

**Table 1:** Neonatal brain segmentation and relative volume calculation.

**Segment**	**Description**	**Relative volume**
**A) Whole brain volumes**		
Intracranial volume (ICV)	All brain segments, excluding extracranial background	−
Total brain volume (TBV)	All brain segments, excluding extracranial background and eCSF	−
Total tissue volume (TTV)	All brain segments, excluding extracranial background, eCSF, and lateral ventricles	−
**B) Total GM or WM Volumes**		
Total cortical GM	Frontal lobe gray matter (GM), temporal lobe GM, parietal lobe GM, occipital lobe GM, insula GM, dingulate GM	/TTV
Total deep GM	Caudate nucleus, lentiform nucleus, thalamus, and intracranial background	/TTV
Total WM	Frontal lobe white matter (WM), temporal lobe WM, parietal lobe WM, occipital lobe WM, insula WM, cingulate WM	/TTV
**C) Regional volumes**		
Total frontal lobe	Frontal lobe GM and WM	/TTV
Total temporal lobe	Temporal lobe GM and WM	/TTV
Total parietal lobe	Parietal lobe GM and WM	/TTV
Total occipital lobe	Occipital lobe GM and WM	/TTV
Total insula	Insula GM and WM	/TTV
Total cingulate	Cingulate GM and WM	/TTV
Posterior fossa	Cerebellum and brainstem	/TTV
Basal ganglia	Caudate nucleus and lentiform nucleus	/TTV
**D.1) Specific tissue volumes**		
	Frontal lobe GM, frontal lobe WM, temporal lobe GM, temporal lobe WM, parietal lobe GM, parietal lobe WM, occipital lobe GM, occipital lobe WM, insula GM, insula WM, cingulate GM, cingulate WM, cerebellum, brainstem, caudate nucleus, lentiform nucleus, thalamus, hippocampus, amygdala	/TTV
**D.2) Specific CSF-filled volumes**		
eCSF	Extra-cerebral cerebrospinal fluid (eCSF), including the third and fourth ventricles.	/ICV
Lateral ventricles	Lateral ventricles, including cavum septum pellucidum (if still present).	/TBV

Each brain segment of interest is accompanied by a description of tissue segments and structures included, and where applicable, how a relative volume was calculated. The table is categorized into A) WBVs, B) total GM or WM volumes, C) regional volumes, D.1) specific tissue volumes, and D.2) specific CSF-filled volumes. The left and right brain regions were consolidated for all labels.

### Normative modeling using Gaussian process regression

Gaussian process regression (GPR) was used to model the development of absolute and relative tissue volumes in the control sample from 32 to 46 weeks PMA at scan. GPR modeling was implemented using GPy in Python (https://sheffieldml.github.io/GPy/; accessed 2021 November 15) as per (Bonthrone et al. 2021; Dimitrova et al. 2021). GPR is a Bayesian non-parametric regression method that provides a point estimate of the average volume and measures of predictive confidence for every observation, whereas accounting for modeled covariates. The difference between predicted and observed values, normalized by the predictive confidence (i.e. standard deviation, SD), represents the deviation of a data point from the expected mean, expressed as a *z*-score in units of SD (Marquand et al. 2016). Subsequently, the GPR model was used to extract individualized absolute and relative volume *z*-scores (by segment, as listed in Table 1) for an independent sample of 25 neonates with DS, accounting for sex, PMA at scan (in weeks), and age from birth (in weeks) variables.

### Statistical analyses

Individual absolute and relative volumetric *z*-scores extracted from GPR modeling were used in ensuing statistical analyses. Shapiro–Wilk and Kolmogorov–Smirnov tests were used to test normality in each dataset. In general, non-parametric tests such as the Mann–Whitney *U* test and Kruskal–Wallis one-way test of variance were used to test statistical difference between groups, including DS vs control, or DS neonates with CHD vs without CHD. Cliff’s delta (*d*, ranging from −1 to 1) was used to assess effect size using the “effsize” package v 0.8.1 in R. Effect sizes were categorized as negligible (*d* ≤ 0.147), small (0.148 ≤ *d* ≤ 0.33), medium (0.34 ≤ *d* ≤ 0.474), or large (*d* ≥ 0.475) (Romano et al. 2006; Torchiano 2020). Extreme deviations in volume were taken as a *z*-score ≤ − 2.6 or ≥ + 2.6 SD, representing the top and bottom 0.5% of the control population, as per (Dimitrova et al. 2021). Simple linear regressions were used to model the relationship between (i) volumetric *z*-scores and PMA at scan and (ii) volumetric *z*-scores and whole brain volume (WBV) *z*-scores (“WBV covariation analysis”) for each brain segment. Linear regression was used for both analyses as non-linear models did not provide significantly improved statistical fit over linear models. For both analyses, median regression (Tau = 0.5) and quartile regressions (Tau = 0.25 and 0.75) were fitted using the “quantreg” package v5.95 in R (Koenker 2005). Akaike information criterion (AIC) was used to assess relative goodness of fit for median regression. Median regressions were used to obtain WBV-adjusted group median *z*-scores as per (McGreevy et al. 2009). For simple linear regressions, the coefficient of determination (R^2^ and adjusted R^2^) was used to indicate goodness of model fit. Spearman’s rank correlation coefficient (Rho, *ρ*) was used to assess the strength of correlation, which was considered very weak from 0 < *ρ ≤* 0.19, weak from 0.20 *≤ ρ ≤* 0.39, moderate from 0.40 *≤ ρ ≤* 0.59, strong from 0.60 *≤ ρ ≤* 0.79 and very strong from 0.80 *≤ ρ ≤* 1.00. The extra sum-of-squares *F*-test (in GraphPad Prism v9.1.1.) was used to test for differences in the slope or intercept (i.e. elevation) parameters of two separate simple linear models (e.g. DS vs control, or DS neonates with CHD vs without CHD). This test compares whether a combined model or two separate linear models provide a better goodness of fit for the data. The result is expressed as an *F* ratio, from which a *P*-value is calculated. For all above analyses, Benjamini and Hochberg’s false discovery rate was applied to correct for multiple comparisons (reported as “pFDR”) and statistical significance was set at pFDR < 0.05. All analyses and visualizations were performed in GraphPad Prism v9.1.1 or R v4.1.0 and 3D brain visualizations were created using HCP workbench (Marcus et al. 2011) or ITKSNAP (Yushkevich et al. 2006).

### Data availability

The dHCP is an open-access project. The imaging data used in this study were included in the third dHCP data release (2021) (Edwards et al. 2022), which can be downloaded by registering and completing a data usage agreement at http://data.developingconnectome.org. Data from the eBiDS study are available from the corresponding author upon reasonable request.

## Results

### Demographic characteristics of participant groups

Demographic information for the DS (*n* = 25, 48.0% female) and control groups (*n* = 493, 49.3% female) are summarized in Table 2. The sex ratio (pFDR = 1.00) and PMA at scan (pFDR = 0.73, *d* = −0.04) were not significantly different between the DS and control groups. However, GA at birth was significantly earlier in the DS group (pFDR = 0.0003, *d* = −0.56).

**Table 2:** Demographic characteristics of participant groups.

	Control	Down Syndrome	pFDR	Sig.	Cliff’s delta (*d*)	Effect size
Sample size (n)	493	25				
GA at birth (weeks), median (IQR) [range]	39.86 (38.14–40.71) [24.71–43.00]	37.14 (36.22–38.07) [31.43–41.71]	**0.0003**	***	−0.56	large
PMA at scan (weeks), median (IQR) [range]	41.00 (39.14–42.57) [31.14–45.14]	40.57 (38.43–43.15) [32.43–45.57]	0.73	ns	−0.04	negligible
Preterm birth < 37 GA, no. (%)	86 (17.5%)	10 (40.0%)	0.05	ns		
Female, no. (%)	243 (49.3%)	12 (48.0%)	1.00	ns		
Non-singleton, no. infants (%)	55 (11.2%)	1 (4.0%)	0.49	ns		
						
Weight at birth (kg), median (IQR)	3.30 (2.80, 3.69)	2.70 (2.39, 3.09)	**0.0003**	***	−0.46	medium
Weight at birth *z*-score^^^, median (IQR)	−0.19 (−0.78, 0.43)	−0.31 (−0.87, 0.38)	0.57	ns	−0.08	negligible
Weight at scan (kg), median (IQR)	3.40 (2.80, 3.80)	3.11 (2.62, 3.50)	0.08	ns	−0.23	small
Weight at scan *z*-score^, median (IQR)	−0.44 (−1.18, 0.19)	−0.98 (−1.82, −0.02)	0.08	ns	−0.22	small
						
HC at birth (cm), median (IQR)	34.0 (33.0, 35.0)	32.0 (31.0, 33.0)	**0.0003**	***	−0.61	large
HC at birth *z*-score^, median (IQR)	−0.04 (−0.84, 0.78)	−0.91 (−1.42, −0.19)	**0.0013**	**	−0.43	medium
HC at scan (cm), median (IQR)	35.0 (33.0, 36.2)	33.4 (32.3, 34.4)	**0.006**	**	−0.34	medium
HC at scan *z*-score^, median (IQR)	−0.12 (−0.98, 0.67)]	−1.06 (−1.90, −0.31)	**0.0016**	**	−0.39	medium

Details of GA at birth, PMA at scan, number of preterm births (< 37 weeks GA), sex and non-singleton neonates (e.g. twins) are provided for both control (*n* = 493) and DS (*n* = 25) groups. Mann–Whitney *U* or Fisher’s exact tests were conducted between the two groups and Benjamini and Hochberg’s FDR multiple comparison correction (pFDR) was applied. Cliff’s delta (*d*) test was used to assess effect size. pFDR < 0.05 are in bold. (^^^) indicates *z*-scores calculated using the RCPCH UK-WHO growth charts and not GPR modeling. [Abbreviations: GA = gestational age at birth, HC = head circumference, IQR = inter-quartile range, PMA = postmenstrual age at scan.]

As a cohort, neonates with DS weighed less at birth (median = 2.70 kg, *d* = −0.46, pFDR = 0.0003). However, after correcting for individual sex and age using *z*-scores derived from the RCPCH UK-WHO growth charts, DS birth weight (median *z*-score = −0.31 SD, *d* = −0.08, pFDR = 0.57) and scan weight (median *z*-score = −0.98 SD, *d* = −0.22, pFDR = 0.082) were not significantly different from control. Neonates with DS had a smaller absolute head circumference (HC) at birth (median = 32.0 cm, *d* = −0.61, pFDR = 0.0003) and at scan (median = 33.4 cm, *d* = −0.34, pFDR = 0.006), which remained smaller than control even after correcting for individual sex and age using *z*-scores (HC median *z*-score at birth = −0.91 SD, *d* = −0.43, pFDR = 0.0013; HC median *z*-score at scan = −1.06 SD, *d* = −0.39, pFDR = 0.0016) (Table 2).

### WBVs and most underlying absolute tissue volumes were significantly smaller in neonates with DS

**Fig. 1:**
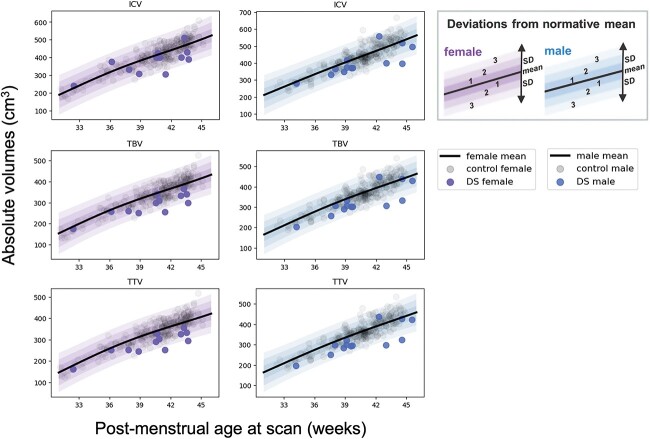
Normative modeling of absolute WBVs in neonates. GPR plots of absolute WBVs for females (in purple) and males (in blue) from 32 to 46 weeks PMA. Descriptions of ICV, TBV, and TTV can be found in Table 1. The normative mean appears as a bolded black curve, whereas shaded areas represent ±1, 2, and 3 standard deviations (SD) from the normative mean. Transparent gray dots represent control neonates (*n* = 243 females and *n* = 250 males). Data for the DS cohort (*n* = 25) are shown for females (purple dots, *n* = 12) and males (blue dots, *n* = 13).

**Fig. 2:**
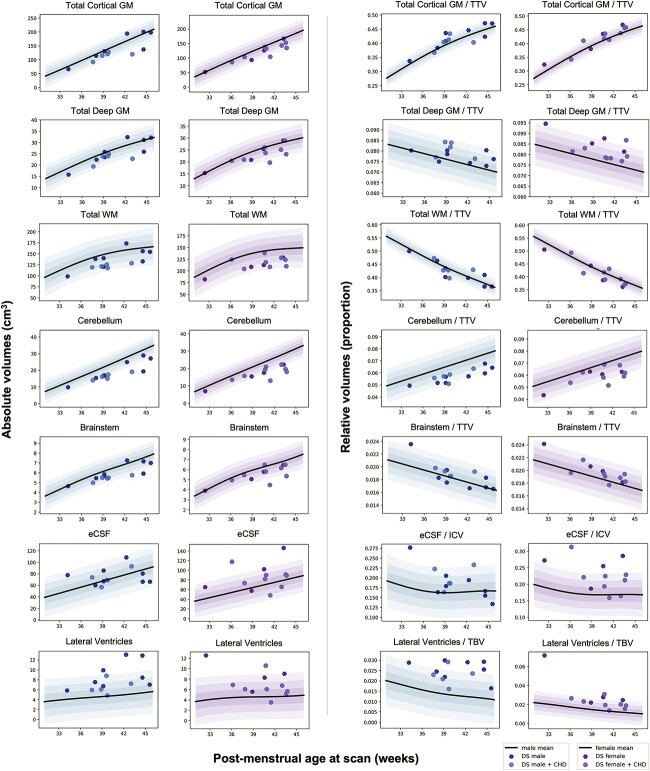
Normative modeling of main tissue classes in absolute and relative volume. GPR modeling of the main tissue classes of the brain from 32 to 46 weeks PMA. Plots for *absolute volumes* (in cm^3^) appear on the left side, whereas plots for *relative volumes* (i.e. proportion of TTV, TBV, or ICV) appear on the right side. The normative mean appears as a black bolded curve, whereas shaded areas represent ±1, 2, and 3 SD from the normative mean. Dots for control neonates are not shown for better visualization. Data for DS neonates (*n* = 25) are shown for females (purple dots, *n* = 12) and males (blue dots, *n* = 13). Lighter shaded dots for both females and males indicate DS neonates with a CHD (*n* = 13, 5 males and 8 females). GPR plots for all specific tissue segments can be found in Supplementary Fig. S1.

**Fig. 3:**
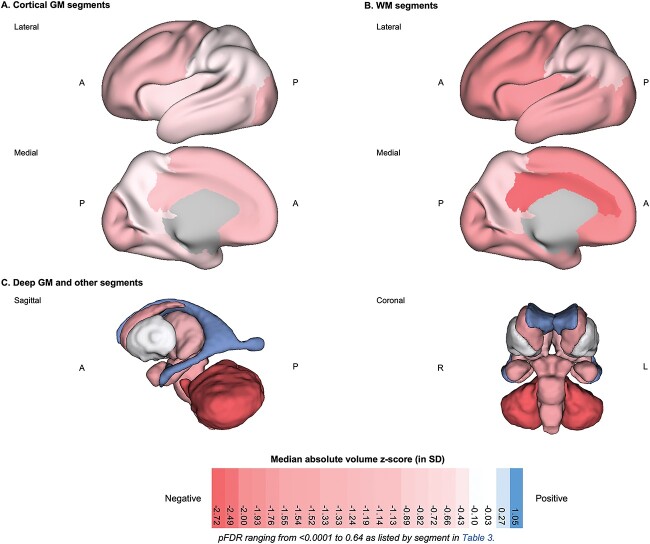
3D brain visualization of the median absolute volume *z*-score by tissue segment for neonates with DS. 3D brain visualization indicating the median absolute volume *z*-score (in SD) by tissue segment for the DS group. pFDR values can be found listed in Table 3 by tissue segment. The median absolute volume *z*-score is indicated by a color scale, whereby red indicates a negative deviation from the normative mean (i.e. *z* < 0, a smaller volume than control), white indicates no significant deviation from control (i.e. *z* ~ 0), and blue indicates a positive deviation (i.e. *z* > 0, a larger volume than control). 3D brain visualization for (a) cortical GM segments, (b) WM segments, and (c) deep GM and other segments. Axes: A = anterior, P = posterior, R = right, L = left.

Absolute volume *z*-scores were extracted from GPR normative modeling for neonates with DS for each tissue segment (Figs 1, 2, and Supplementary Fig. S1) and compared with the control group (Table 3 and Fig. 3).

**Table 3:** Groupwise comparison of absolute volume *z*-scores.

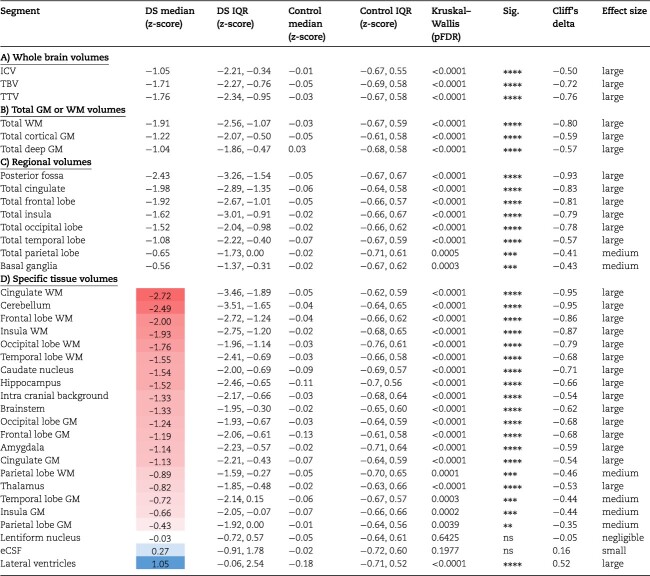

Table of median absolute volume *z*-score and interquartile range (IQR) for DS and control groups. The table is organized into A) WBVs, B) total GM or WM volumes, C) regional volumes, and D) specific tissue volumes (including CSF-filled volumes). A non-parametric Kruskal–Wallis test with Benjamini and Hochberg’s FDR multiple comparison correction (pFDR) was performed for each tissue segment. Cliff’s delta (*d*) was used to assess the effect size. A color scale has been applied, whereby red indicates a negative deviation from the normative mean (i.e. *z* < 0, a smaller volume than control), white indicates no significant deviation from control (i.e. z ~ 0), and blue indicates a positive deviation (i.e. *z* > 0, a larger volume than control).

Absolute WBVs were significantly smaller in neonates with DS compared with control, for sex and age, with large effect sizes (ICV median *z*-score = −1.05 SD, *d* = −0.50; TTV median *z*-score = −1.76 SD, *d* = −0.76; pFDR < 0.0001) (Table 3A). As WBVs were significantly smaller in DS, most underlying absolute tissue volumes were also significantly smaller than control for sex and age (Table 3D). This was clearly evidenced by the large number of segments in red listed in Table 3D (i.e. indicating a median *z*-score < 0) and seen visually in Fig. 3. Only the lentiform nuclei (comprising the putamen and pallidum) (median *z*-score = −0.03 SD, *d* = −0.05, ns) and the eCSF (median *z*-score = 0.27, *d* = +0.16, ns) were not significantly different from control, whereas the lateral ventricles were significantly enlarged (median *z*-score = +1.05 SD, *d* = +0.52, pFDR < 0.0001). Here, we must note that we were not able to assess the contribution of the *cavum septum pellucidum* on total lateral ventricular volume, nor the contribution of the third and fourth ventricles on the total eCSF volume, as our neonatal segmentation programme does not sub-segment these structures (Makropoulos et al. 2014, 2016, 2018).

Of particular interest, we observed that the total cerebral WM (median *z*-score = −1.91 SD, *d* = −0.80, pFDR < 0.0001) was significantly smaller in neonates with DS compared with control and was further deviated from the normative mean than total cortical GM (median *z*-score = −1.22 SD, *d* = −0.59, pFDR < 0.0001) (Table 3B). Lastly, from a regional perspective, the parietal lobe (median *z*-score = −0.65 SD, *d* = −0.41, pFDR = 0.0005), and the basal ganglia (comprising the caudate and lentiform nuclei) (median *z*-score = −0.56 SD, *d* = −0.43, pFDR = 0.0003) were less deviated from the normative mean than other regions in absolute volume (Table 3C).

### Relative volumes demonstrated regions with significantly altered tissue proportionality across the neonatal brain in DS

**Fig. 4:**
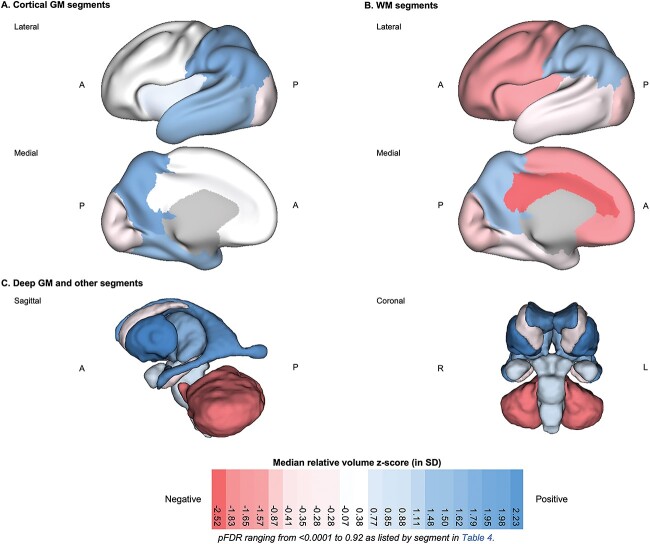
3D brain visualization of the median relative volume *z*-score by tissue segment for neonates with DS. 3D brain visualization indicating the median relative volume *z*-score (in SD) by tissue segment for the DS group. pFDR values can be found listed in Table 4 by tissue segment. The median relative volume *z*-score is indicated by a color scale, whereby red indicates a negative deviation from the normative mean (i.e. *z* < 0, a smaller proportion of WBV than control), white indicates no significant deviation in proportion from control (i.e. z ~ 0), and blue indicates a positive deviation (i.e. *z* > 0, a larger proportion of WBV than control). 3D brain visualizations for (a) cortical GM segments, (b) WM segments, and (c) deep GM and other segments. Axes: A = anterior, P = posterior, R = right, L = left.

**Fig. 5:**
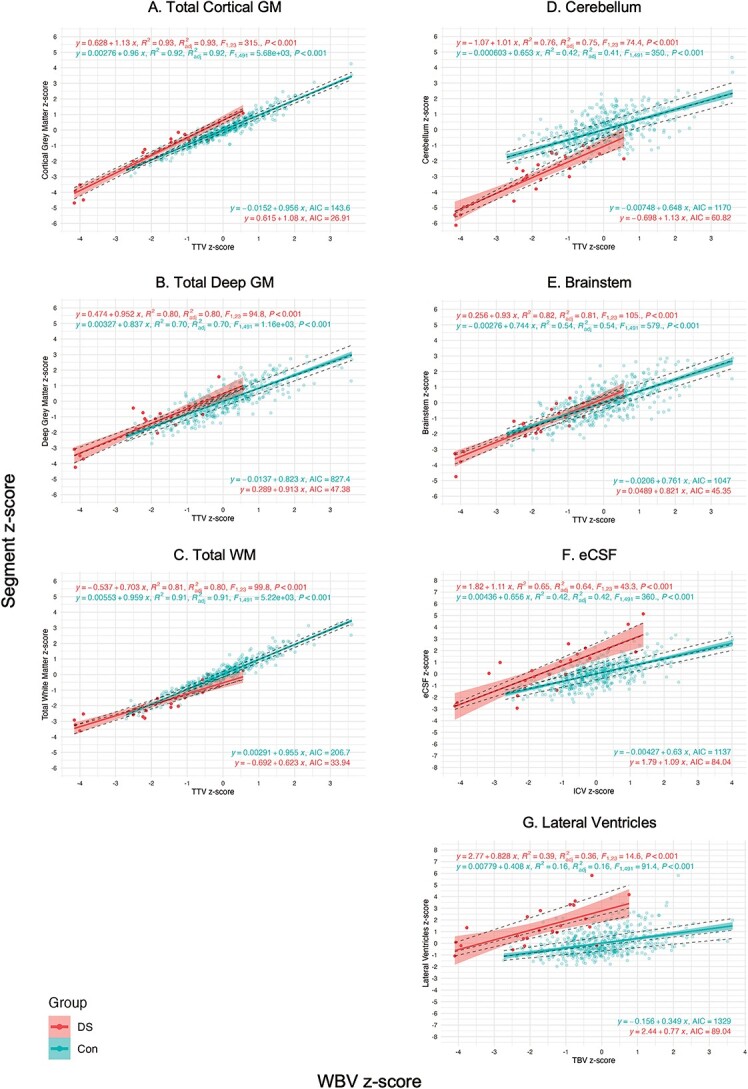
Covariation analysis of absolute volume *z*-scores against WBV *z*-scores using linear and median regression. Plots of absolute volume *z*-scores against WBV *z*-scores (i.e. ICV, TBV, or TTV). Plots for the (a) total cortical GM, (b) total deep GM, (c) total WM, (d) cerebellum, (e) brainstem, (f) eCSF, and (g) lateral ventricles. Dots for control neonates (*n* = 493, females and males) appear in blue, with colored linear regressions and colored 95% confidence intervals. Dots for neonates with DS (*n* = 25, females and males) appear in red, with colored linear regressions and colored 95% confidence intervals. Additionally, dotted black lines indicate quartile regressions (first quartile, median, and third quartile). Parameters for the linear regressions appear in the top left (i.e. equation, *R*^2^, adjusted *R*^2^, *F*- and *P*-value), whereas parameters for the median regressions appear in the bottom right of each graph (i.e. equation and AIC). Plots for all other specific tissue segments can be found in Supplementary Fig. S5. A table comparing WBV-adjusted median *z*-scores in DS vs control groups can be found in Supplementary Table S7.

Although, absolute volume *z*-scores showed that most tissue segments were significantly smaller in the DS group compared with control, the use of *z*-scores derived from relative volumes (i.e. tissue volume as a proportion of WBV, as per Table 1) revealed a detailed picture of regions with significantly altered tissue proportionality across the neonatal brain in DS (Table 4 and Fig. 4). In addition to this proportionality analysis, we also conducted a covariation analysis (of absolute volume *z*-scores and WBV *z*-scores) for each tissue segment, found in Fig. 5 and Supplementary Fig. S5. This covariation exercise was a useful complementary analysis, as different regions of the brain vary in their scaling with WBV, even in the control population. Table S7 shows WBV-adjusted median *z*-scores and group differences after multiple comparison correction. The proportionality and covariation analyses showed predominantly the same results across all regions and tissue types. Only two tissue segments differed in test results and are detailed below.

**Table 4:** Groupwise comparison of relative volume *z*-scores.

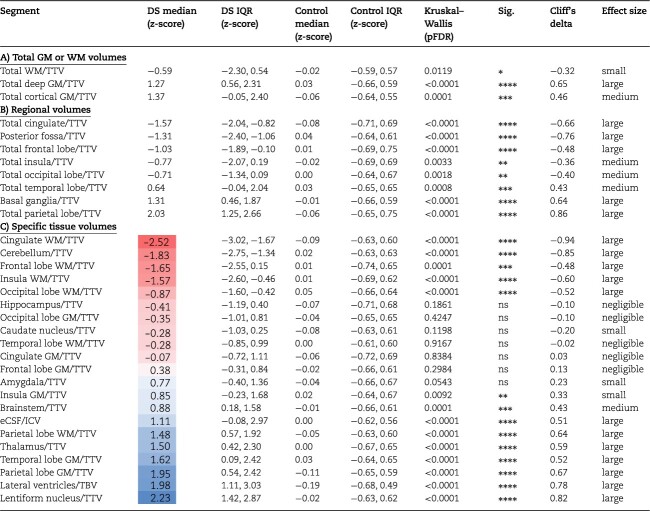

Table of median relative volume *z*-score and interquartile range (IQR) for DS and control groups. The table is organized into **A)** total GM or WM volumes, **B)** regional volumes, and **C)** specific tissue volumes (including CSF-filled volumes). A non-parametric Kruskal–Wallis test with Benjamini and Hochberg’s FDR multiple comparison correction (pFDR) was performed for each tissue label. Cliff’s delta (*d*) test was used to assess effect size. A color scale has been applied, whereby red indicates a negative deviation from the normative mean (i.e. *z* < 0, a smaller proportion of WBV than control), white indicates no significant deviation in proportion from control (i.e. z ~ 0), and blue indicates a positive deviation (i.e. *z* > 0, a larger proportion of WBV than control).

First, we observed that five tissue segments (i.e. the cerebellum, as well as the cingulate, frontal, insular and occipital WM segments) were significantly smaller in relative volume with large effect sizes (*d* = −0.52 to −0.94, pFDR < 0.0001), as indicated by darker red tones in Fig. 4 (also in Table 4C and Table S7).

Total cortical GM was significantly enlarged (median *z*-score = +1.37 SD, *d* = +0.46, pFDR = 0.0001), whereas conversely, total cerebral WM was significantly reduced in relative volume compared with control (median *z*-score = −0.59 SD, *d* = −0.32, pFDR = 0.012) (Table 4A). Looking at specific tissue segments, we observed regional differences in cortical GM and WM proportions. Most cortical GM segments showed no significant difference (i.e. occipital, cingulate, frontal GM segments; *d* = −0.10 to +0.13, pFDR = 0.30 to 0.84) or were significantly enlarged in relative volume (i.e. insular, temporal, and parietal GM segments; *d* = +0.33 to +0.67, pFDR = 0.0092 to < 0.0001). For additional information, however, the frontal GM was significantly enlarged after WBV-adjustment using the covariation analysis (DS adjusted median *z*-score = 0.31, pFDR = 0.0006, Table S7).

Comparatively, most WM segments were significantly smaller (i.e. cingulate, frontal, insular and occipital WM segments; *d* = −0.94 to −0.52, pFDR < 0.0001), except for the temporal WM, which showed no significant difference (median *z*-score = −0.28 SD, *d* = −0.02, pFDR = 0.92) and the parietal WM, which was significantly enlarged in relative volume (median *z*-score = +1.48 SD, pFDR < 0.0001, *d* = +0.64) (Table 4C). Thus, from a total lobar perspective (i.e. GM + WM), we noticed a regional pattern, whereby the temporal and parietal lobes were relatively enlarged, whereas the frontal, cingulate, insular, and occipital lobes were reduced in relative volume compared with control (Table 4B and Supplementary Table S7).

Within the deep GM, the lentiform nuclei (which were not significantly different in absolute volume, Table 3D) were disproportionately enlarged in relative volume (median *z*-score = +2.23 SD, *d =* +0.82, pFDR < 0.0001) (Table 4C). The thalami were also significantly enlarged (median *z*-score = +1.50 SD, *d =* +0.59, pFDR < 0.0001), whereas the caudate nuclei showed no difference in relative volume (median *z*-score = −0.28 SD, *d =* −0.20, pFDR = 0.12).

Within the posterior fossa, the cerebellum and the brainstem exhibited different dynamics. The cerebellum was significantly and disproportionately small with a very large effect size (median *z*-score = −1.83 SD, *d* = −0.85, pFDR < 0.0001), whereas conversely, the brainstem was significantly enlarged in relative volume with a medium effect size (median *z*-score = 0.88 SD, *d* = +0.43, pFDR = 0.0001). However, for additional information, the brainstem showed no significant difference after WBV-adjustment (DS adjusted median *z*-score = 0.05, pFDR = 0.09) (Supplementary Table S7).

CSF-filled volumes were significantly enlarged in relative volume. The lateral ventricles were disproportionately enlarged with a very large effect size (median *z*-score = +1.98 SD, *d =* +0.78, pFDR < 0.0001), although the contribution of the *cavum* was not assessed separately. The eCSF, which was not significantly different from control in absolute volume (Table 3D), was significantly enlarged in relative volume with a large effect size (median *z*-score = +1.11 SD, *d =* +0.51, pFDR < 0.0001) (Table 4C), although the contribution of the third and fourth ventricles was not assessed. Lastly, the hippocampus and the amygdala were not significantly different in relative volume compared with control (*d* = −0.10 to +0.23, pFDR = 0.054 to 0.19).

### The effect of age at scan on neonatal brain volumes in DS

The neonatal period from 32 to 46 weeks PMA at scan was marked by a phase of rapid brain development for control neonates. Absolute and relative volumetric development in the preterm- to term-born control cohort was characterized in detail and can be found as supplementary information in Supplementary Fig. S2 and Table S4.

**Fig. 6:**
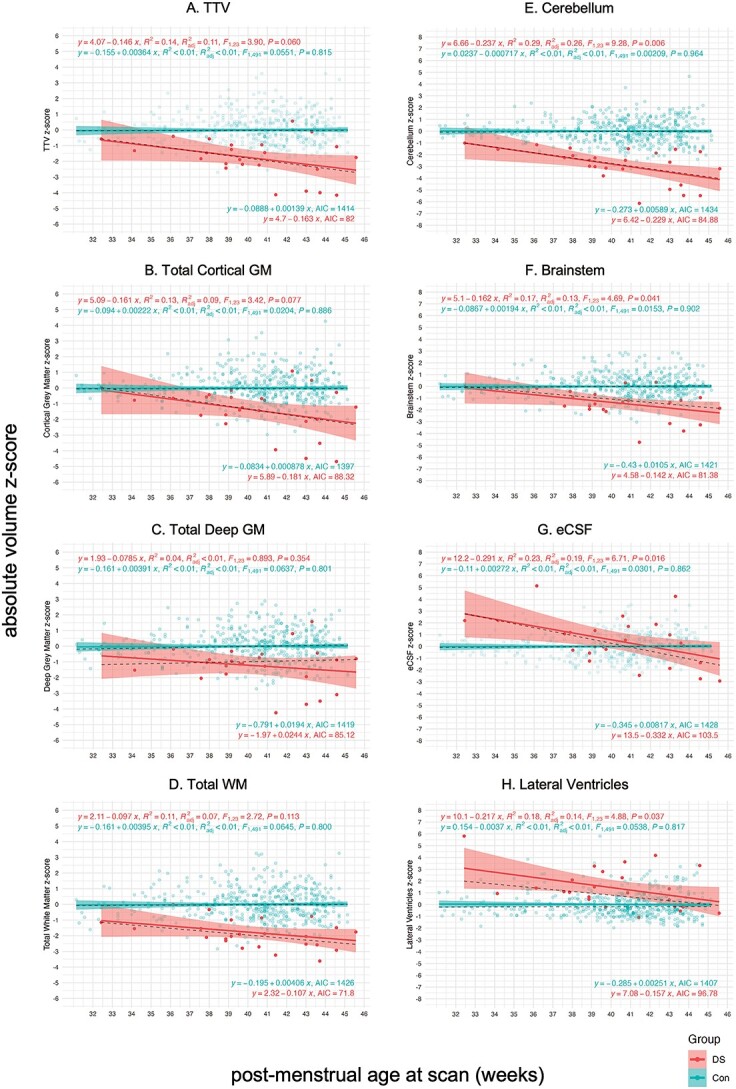
Simple linear regression of absolute volume *z*-scores against PMA at scan for whole brain and main tissue volumes. Simple linear regression plots of absolute volume *z*-scores against PMA at scan from 32 to 46 weeks for (a) the whole brain (i.e. TTV) and main tissue classes of the brain, including (b) the total cortical GM, (c) the total deep GM, (d) the total WM, (e) the cerebellum, (f) the brainstem, (g) the eCSF, and (h) the lateral ventricles. Dots for individual control neonates (*n* = 493, females and males consolidated) appear in blue, and linear regressions appear as flat blue lines at *z* = 0 with 95% confidence intervals. Dots for individual neonates with DS (*n* = 25, females and males) appear in red, and linear regressions appear as red lines with 95% confidence intervals. For additional information, dotted black lines indicate the median regression. Parameters for DS and control linear regressions appear in the top left (i.e. equation, *R*^2^, adjusted *R*^2^, *F*- and *P*-value), whereas parameters for the median regressions appear in the bottom right of each graph (i.e. equation and AIC). Linear regression plots for all other specific tissue segments can be found in Supplementary Fig. S3 and a table of results for *F*-tests in Supplementary Table S5.

Simple linear regression models plotting absolute volume *z*-scores against PMA at scan were used to appreciate age-related change in volumetric *z*-scores in the DS cohort compared with control (main tissue classes in Fig. 6A–H, and all specific segments in Supplementary Fig. S3). Linear regressions for the control group were characterized by a flat line at the normative mean (z = 0) for all ages at scan. Statistical results for the extra sum-of-squares *F*-tests are summarized in Supplementary Table S5 and the Spearman’s rank correlation tests in Supplementary Table S6.

Linear regressions for WBVs in the DS group displayed negative slopes that were significantly different from control (e.g. ICV, slope *F* ratio = 11.45, pFDR = 0.0021; TTV, slope *F* ratio = 5.42, pFDR = 0.034, Supplementary Table S5), indicating a gradual deviation from the control mean with advancing PMA at scan. As such, WBVs for neonates with DS were closer to the control mean when born and scanned preterm (i.e. < 36 weeks PMA), but were markedly smaller than control for neonates scanned after term age (approximately 37 to < 46 weeks PMA) (Fig. 6A and Supplementary Fig. S3). There was pronounced individual variability in neonates scanned at later ages, where there appeared to be a bimodal distribution of *z*-scores in which some neonates displayed typical WBVs for DS, whereas others displayed extreme negative deviations (i.e. four neonates with TTV, z ≤ −2.6 SD). The topic of individual variability in WBV, particularly at later ages at scan, is covered in a sub-section (below) pertaining to neonates with CHD.

As per WBV, many underlying tissue classes also displayed a gradual deviation from the control mean with advancing PMA (Fig. 6B–H, Supplementary Table S5), including the total cortical GM (slope *F* ratio = 6.32, pFDR = 0.022), the brainstem (slope *F* ratio = 6.36, pFDR = 0.022), and the cerebellum (slope *F* ratio = 13.15, pFDR = 0.0009). It is worth noting that cerebellar deviation was particularly pronounced in neonates with DS, with twelve neonates displaying extreme negative deviations (z ≤ −2.6 SD) (Fig. 6E). Although total deep GM and total WM (Fig. 6C and D) also displayed negative slopes, these were not significantly different from control (deep GM, slope *F* ratio = 1.63, pFDR = 0.27; WM, slope *F* ratio = 2.52, pFDR = 0.16) (Supplementary Table S5).

With regards to CSF-filled segments, the slope for eCSF (Fig. 6G) was significantly different from control (slope *F* ratio = 19.05, pFDR = 0.0003, Supplementary Table S5). Individual *z*-scores for the eCSF were markedly larger than the control mean (i.e. +5.1 > *z* > +2 SD) for neonates born and scanned preterm, indicating a tendency for excessive eCSF at these ages. However, these gradually decreased by later ages at scan, although individual variability remained high across neonates. The lateral ventricles (Fig. 6H) tended to be consistently larger than the control mean at all ages. After removing an outlier with an extreme positive deviation (z = +5.8, scanned at 32.4 weeks PMA), possibly due to early preterm birth, the slope for the lateral ventricles was not significantly different from control (slope *F* ratio = 1.61, pFDR = 0.21). However, the elevation was significantly different (intercept *F* ratio = 29.40, pFDR = 0.0003) indicating the presence of consistently enlarged lateral ventricles for sex and age across the DS cohort (although the contribution of the *cavum* was not assessed separately) (Supplementary Table S5).

For further detail on all specific tissue segments, linear regression plots can be found in Supplementary Fig. S3 and results for statistical tests in Supplementary Tables S5–S6. Of particular note, the slope and intercept for the lentiform nuclei (Supplementary Fig. S3D) were not different from control (slope *F* ratio = 0.03, pFDR = 0.86; intercept *F* ratio = 0.54, pFDR = 0.49). Thus, the lentiform nuclei represented the only brain segment in the DS cohort to develop without any observable difference to control neonates from 32 to 46 weeks PMA.

### The impact of CHD on neonatal brain volumes in DS

To assess the possible impact of CHD on neonatal brain volumes in DS, neonates were further categorized into subgroups with CHD (CHD+, *n* = 13) and without CHD (CHD−, *n* = 12) (Supplementary Table S2). Overall, CHD+ and CHD− neonates did not show any statistically significant groupwise differences in regional absolute volume after FDR multiple comparison correction (Supplementary Table S8). This was most likely due to low statistical power, as CHD subgroup sizes were small, and due to the large number of multiple comparisons. However, certain underlying trends were observed from the uncorrected *P*-values, which are discussed in Supplementary Table S8.

**Fig. 7:**
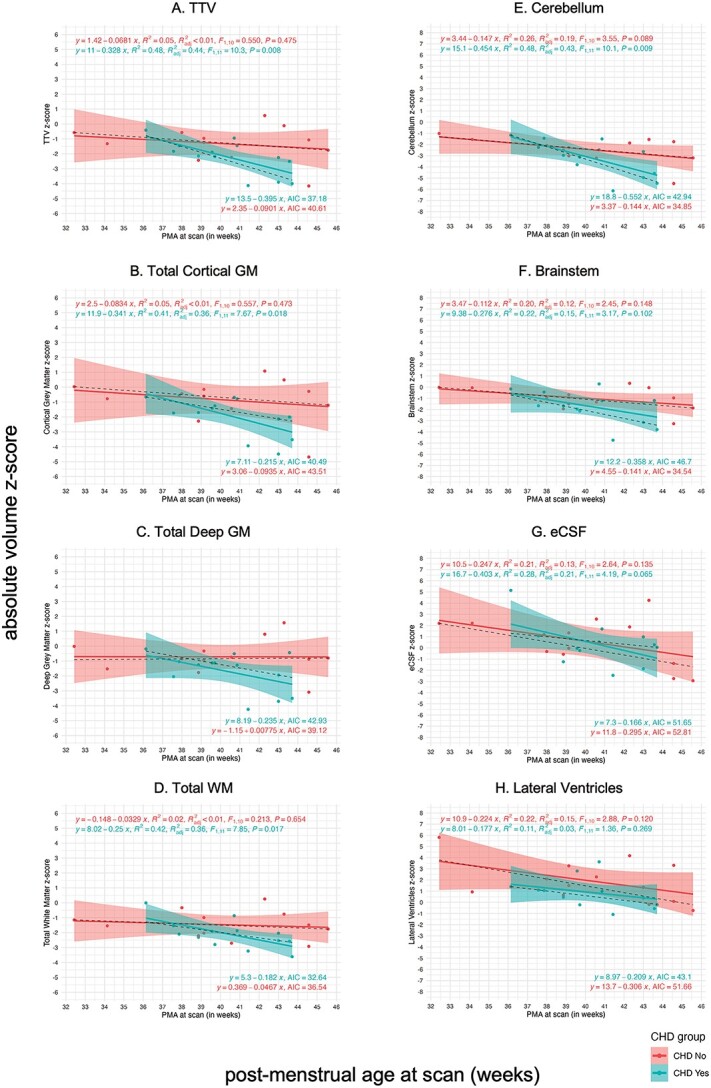
Simple linear regression of absolute volume *z*-scores against PMA at scan for whole brain and main tissue volumes in CHD+ and CHD− neonates with DS. Simple linear regression plots of absolute volume *z*-scores against PMA at scan from 32 to 46 weeks for (a) the WBV (i.e. TTV) and main tissue classes of the brain, including (b) the total cortical GM, (c) the total deep GM, (d) the total WM, (e) the cerebellum, (f) the brainstem, (g) the eCSF, and (h) the lateral ventricles. CHD+ neonates with DS (*n* = 13) appear in blue, whereas CHD− neonates with DS (*n* = 12) appear in pink. Linear regressions appear as colored lines with 95% confidence intervals, whereas for additional information, dotted black lines indicate the median regression. Parameters for the linear regressions appear in the top left (i.e. equation, *R*^2^, adjusted *R*^2^, *F*-statistic, and *P*-value), whereas parameters for the median regressions appear in the bottom right of each graph (i.e. equation and AIC). Plots for all other specific tissue segments can be found in Supplementary Fig. S4 and a table of results for *F*-tests in Supplementary Table S9.

To delve further into the impact of CHD on neonatal brain volumes, we used simple linear regressions plotting absolute volumetric *z*-scores against PMA at scan in order to observe differences in age-related volumetric change between subgroups. Linear regression plots can be found in Fig. 7 (i.e. main tissue classes) and Supplementary Fig. S4 (i.e. all specific tissue segments), as well as results for the extra sum-of-squares *F*-test (in Supplementary Table S9) and the Spearman’s rank correlation test (in Supplementary Table S10).

**Fig. 8:**
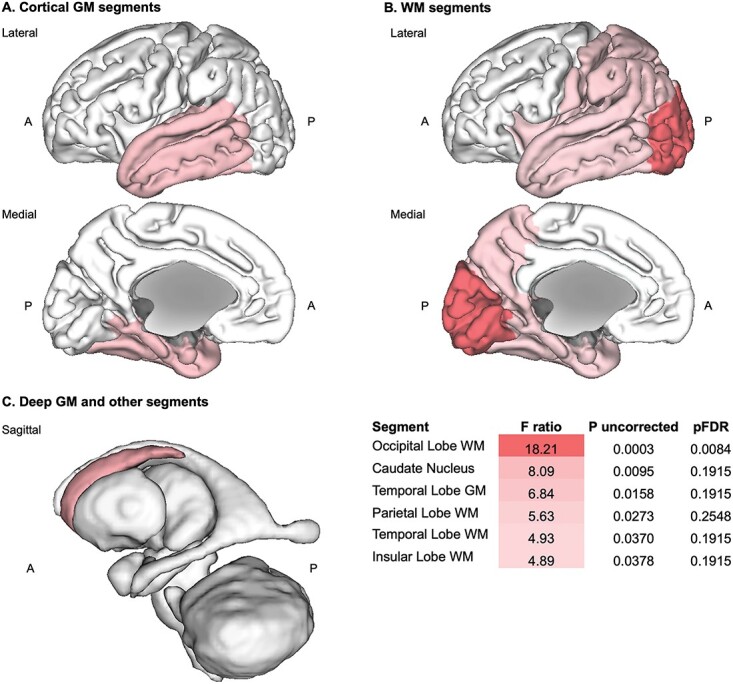
Age-related differences between CHD+ and CHD− neonates with DS. 3D brain visualization of the *F* ratio (from the extra sum-of-squares *F*-test) for tissue segments, in which the slope or intercept of CHD+ and CHD− linear regressions were significantly different prior to multiple comparison correction (*P* uncorrected < 0.05) between CHD+ and CHD− neonates with DS. Importantly, only the *F*-test for the occipital WM remained significant after multiple comparison correction (pFDR < 0.05) (see Supplementary Table S9). Axes: A = anterior, P = posterior.

Only one tissue segment, the occipital WM (Fig. 8 and Supplementary Fig. S4C), showed a statistically significant difference in slope between CHD+ and CHD− neonates after multiple comparison correction (slope *F* ratio = 18.21, pFDR = 0.0084) (Supplementary Table S9). In CHD+ neonates, the occipital WM segment also displayed a significant and very strong negative correlation with PMA (*ρ* = −0.89, *R*^2^ = 0.78, uncorrected *P*-value < 0.0001, pFDR = 0.0027), which was not the case for CHD− neonates (*ρ* = +0.15, *R*^2^ = 0.05, uncorrected *P*-value = 0.63, pFDR = 0.85) (Supplementary Table S10). Thus, absolute occipital WM volume was significantly reduced in CHD+ neonates compared with CHD− neonates by later ages at scan (from approximately 40 weeks PMA).

Although several other tissue segments displayed differences between CHD+ and CHD− neonates on the *F*-test and/or the Spearman’s test (uncorrected *P*-value < 0.05), these did not survive multiple comparison correction (pFDR < 0.05) (Supplementary Tables S9 and S10). This was most likely due to low statistical power, as CHD subgroup sizes were small, and due to the large number of multiple comparisons. However, we report certain underlying trends, which were observed from the uncorrected *P*-values for completeness of information, as this is a rare clinical cohort.

Firstly, WBV in CHD+ neonates tended to be smaller than CHD− neonates by later ages at scan. ICV and TTV in CHD+ neonates displayed strong negative correlations with advancing PMA at scan (ICV, *ρ* = −0.67, *R*^2^ = 0.48, uncorrected *P*-value = 0.015, pFDR = 0.05; TTV, *ρ* = −0.73, *R*^2^ = 0.48, uncorrected *P*-value = 0.0059, pFDR = 0.05), which was not the case for CHD− neonates (e.g. TTV, *ρ* = −0.18, *R*^2^ = 0.05, uncorrected *P*-value = 0.58, pFDR = 0.85) (Fig. 7A. and Supplementary Fig. S4A, Table S10). Upon examining individual volumetric *z*-scores and detailed CHD information (see Supplementary Table S2), we noted that three out of four neonates with extreme negative deviations in TTV (i.e. *z* ≤ −2.6 SD) had several cardiac defects in addition to being scanned late (after 41 weeks PMA). These neonates also tended to display low oxygen saturation scores at time of scan. One neonate had an AVSD and Tetralogy of Fallot, another displayed an AVSD with coarctation of the aorta, whereas the third had an ASD with persistent *patent foramen ovale*. Although the fourth neonate did not have a CHD, according to clinical notes at time of scan, this neonate suffered from thrombocytopenia and particularly poor feeding in the first few weeks of life. Thus, in certain neonates, it is possible that CHD and low oxygen saturation may be leading to a gradual deviation in WBV from the DS baseline with advancing age at scan.

Much like WBV, several underlying specific tissue segments were negatively correlated with advancing PMA prior to multiple comparison correction in CHD+ neonates, and not in CHD− neonates. These segments were the cerebellum, the frontal, parietal and occipital GM segments, the parietal WM, the hippocampi, and the thalami (*ρ* = −0.59 to −0.71, *R*^2^ = 0.35 to 0.49, uncorrected *P*-value = 0.036–0.008, pFDR = 0.07–0.05) (Supplementary Table S10). Fig. 8 displays the tissue segments for which the slope or intercept of CHD+ and CHD− linear regressions were significantly different using the *F*-test prior to multiple comparison correction (*P* uncorrected < 0.05) (Supplementary Table S9). These were the caudate nuclei, the temporal GM and WM, the parietal WM, and the insular WM (*F* ratio = 4.89 to 8.09, uncorrected *P*-value = 0.038–0.0095, pFDR = 0.19 for all segments). These segments appeared to be particularly clustered in the lateral and posterior regions of the brain, aside from the caudate nucleus. In future, the brain regions with significant age-related volumetric differences between CHD+ and CHD− neonates may become more evident with larger subgroup sizes and higher statistical power.

### Assessing the impact of other clinical comorbidities on neonatal brain volumes in DS

The possible impact of other clinical comorbidities on neonatal brain volumes were also assessed. Other clinical comorbidities for neonates with DS can be found listed in Supplementary Table S3. Gastrointestinal (GI) malformations, including duodenal atresia and Hirschsprung’s disease, were present in 8 out of 25 (32%) of neonates with DS. The same analyses as per CHD+ vs CHD− subgroups were trialed with GI+ and GI− subgroups, as well as other clinically defined subgroups, but these did not show any significant results (data not shown). This was most likely due to small subgroup size, as well as multiple comorbidities. For example, four out of eight neonates with a GI malformation also had CHD. A compounded risk factor for multiple comorbidities was trialed (data not shown), but this did not yield any statistically significant results either.

## Discussion

In this study, we conducted the first comprehensive volumetric phenotyping of the neonatal brain in DS. To the best of our knowledge, the eBiDS study represents the largest dataset of *in vivo* brain imaging in neonates with DS as part of a prospective study. Robust normative modeling allowed individual inference of volumetric deviation from the normative mean for a given sex, age at scan and age from birth. Although we had a small sample size of neonates with DS, the use of individualized *z*-scores for each brain segment greatly improved the sensitivity of our analysis compared with traditional volumetry using raw absolute volumes (*data not shown*).

In this study, we were able to corroborate prior neuroimaging findings from fetuses with DS (Patkee et al. 2020; Tarui et al. 2020), and identify novel volumetric characteristics of the developing brain in DS that had not been documented before. Furthermore, we were able to observe age-related volumetric differences between neonates with CHD and without CHD, indicating that there may be a baseline brain phenotype in neonates with DS, which is further altered in the presence of CHD in early postnatal life. The following sections discuss how our key findings relate to other *in vivo* pediatric neuroimaging studies, as well as observations from post-mortem tissue analyses of the developing brain in DS.

### Several features of the neonatal brain phenotype in DS appear to follow a developmental continuum from infancy to adulthood

We observed several volumetric features of the neonatal brain phenotype in DS, which were consistent with observations in older-age cohorts, and that appeared to follow a developmental continuum. Firstly, WBV was significantly smaller in neonates with DS, in line with other pediatric neuroimaging studies ranging from fetal (< 28 gestational weeks, GW) (Patkee et al. 2020) through to adolescent stages (Jernigan and Bellugi 1990; Jernigan et al. 1993; Pinter et al. 2001b; Kates et al. 2002; Menghini et al. 2011; Śmigielska-Kuzia et al. 2011; Lee et al. 2016; McCann et al. 2021). WBV also remains smaller in adults with DS (Weis et al. 1991; Kesslak et al. 1994; Raz et al. 1995), although it is important not to confound the effects of early onset Alzheimer’s disease, for which the mean age of diagnosis is 55 years (Sinai et al. 2017; Antonarakis et al. 2020).

Despite a significantly smaller WBV, CSF-filled compartments tended to be enlarged in neonates with DS. The lateral ventricles were consistently enlarged in both absolute and relative volume throughout the neonatal period, although the contribution of the *cavum* was not assessed separately. The lateral ventricles were previously found to be enlarged in fetuses as early as 28 GW onwards (Patkee et al. 2020). Hydrocephalus, fetal ventriculomegaly (especially in very low birth weight infants < 1,500 g), and enlargement of the third ventricle, have been reported to occur in DS (Farrell 1994; Schimmel et al. 2006; Movsas et al. 2016; Marano et al. 2020; Varagur et al. 2022). Recently, McCann et al. have also demonstrated continued enlargement of the lateral ventricles from childhood to young adulthood in DS (McCann et al. 2021). Consistent with this, in certain DS murine models (e.g. Ts1Cje, Ts1Rhr), overexpression of purkinje cell protein 4 (pcp4) impaired ciliary function in the ependymal cells of the choroid plexus, resulting in ventriculomegaly, a process that may potentially underlie ventricular enlargement in human DS, amongst other underlying mechanisms (Raveau et al. 2017).

With regards to the eCSF, we observed that neonates with DS born and scanned preterm (< 37 weeks PMA) exhibited enlarged relative eCSF volumes, which gradually decreased by later ages at scan. The contributions of the third and fourth ventricles to total eCSF volume were not assessed individually. In children with DS, relative eCSF volume was non-significantly enlarged from 0 to 5 years old, but no longer after this age range (McCann et al. 2021). eCSF is known to be significantly enlarged in prematurely born infants scanned at term equivalent age (Dimitrova et al. 2021). As such, eCSF enlargement appeared to be a feature of prematurity in our neonatal cohort, but not a long-term characteristic of the DS neonatal brain phenotype.

Decreased cerebellar volume, often referred to as cerebellar hypoplasia, is a well-recognized feature of the brain in DS (Hamner et al. 2018; Antonarakis et al. 2020). Cerebellar hypoplasia refers to a cerebellum of reduced volumed with a predominantly preserved shape (Accogli et al. 2021). Smaller cerebellar volume has been evidenced in fetuses (< 28 GW) (Patkee et al. 2020; Tarui et al. 2020), toddlers (Gunbey et al. 2017), in children (Pinter et al. 2001b), adolescents (Jernigan and Bellugi 1990; Jernigan et al. 1993; McCann et al. 2021), and adults with DS (Aylward et al. 1997a). We found that relative cerebellar volume was significantly and disproportionately small in neonates with DS, representing one of the smallest structures in comparison to control neonates. We also noted that absolute cerebellar volume continued to deviate from the normative mean with advancing age at scan during this neonatal period of typically rapid cerebellar development. Post-mortem examinations have evidenced reduced cerebellar transversal diameter from as early as 15 GW (Guihard-Costa et al. 2006), and significant hypocellularity in all cerebellar layers, which is likely due to impaired proliferation of cerebellar precursor cells in DS (Guidi et al. 2011).

Volumetric features of the deep GM, cortical GM and WM segments also appeared to follow a developmental continuum and are discussed in dedicated sections below. Lastly, regarding the hippocampus and amygdala, we found that, although these were significantly smaller in absolute volume, they were not significantly different from control in relative volume in our neonatal sample. In children (Pinter et al. 2001a) and adults with DS without dementia (Aylward et al. 1999), hippocampal volume was significantly smaller, but the amygdala was not significantly different from control in relative (or WBV-adjusted) volume. From a histological perspective, post-mortem analyses have shown evidence of decreased cell proliferation and increased apoptotic cell death in the hippocampi of fetuses with DS from 17 to 21 GW (Guidi et al. 2008).

### Deep GM structures are proportionally enlarged in neonates with DS

In this study, we observed that the total deep GM was significantly enlarged in relative volume in neonates with DS. We observed that the lentiform nuclei (comprising the putamen and pallidum) and the thalami were proportionally enlarged, whereas the caudate nuclei were not significantly different from control in relative volume.

The proportional enlargement of deep GM structures, particularly the lentiform nuclei, has been observed in several pediatric neuroimaging studies in DS. In toddlers, Gunbey et al. found that the putamen and pallidum (particularly the right sides) were not significantly different from control in absolute volume, suggesting that these would be proportionally enlarged in relative volume (Gunbey et al. 2017). Moreover, McCann et al. demonstrated that the putamen, more so than the pallidum, was enlarged as a proportion of total ICV from childhood to young adulthood in DS (McCann et al. 2021). In adults with DS, without dementia, relative putamen volume was also significantly enlarged (Aylward et al. 1997b), illustrating how this is likely to be a lifelong feature of the brain in DS, with an early developmental origin.

Lastly, in a cohort of children and young adults with DS, Pinter et al. found that WBV-adjusted deep GM volume was significantly enlarged and “*selectively preserved* […] *in the context of significantly smaller overall cerebral volumes”*, which they discuss may suggest “*there is a temporal dissociation for the development of cortical versus subcortical [deep GM] regions”* in DS (Pinter et al. 2001b). Although, the proportional enlargement of deep GM structures may suggest the “selective preservation” of these structures in volume, we would note that this does not exclude possible dysfunction, and could still reflect underlying issues, such as insufficient programmed cell death during development or aberrant neurocircuitry.

During embryonic brain development, discrete regions for the thalami and the basal ganglia (including the caudate, putamen, and pallidum) can be observed by approximately 56 days post-conception (~ 8 post-conception weeks (pcw), Carnegie stage 23) (O’Rahilly and Mller 2006; ten Donkelaar et al. 2014). It has been suggested that the thalami and basal ganglia may develop relatively normally throughout embryonic brain development (up to ~ 8 pcw) before the onset of major alterations in cortical GM and WM development throughout fetal brain development in DS (Pinter et al. 2001b). Thus, in future, it would be of great interest to better understand which morphogenetic processes are most affected during embryonic and fetal brain development in DS, and how these may be differentially impacted by the triplication of *Hsa21*.

### Regional differences in relative cortical GM and WM volumes may be linked to brachycephaly in DS

The regional dynamics in cortical GM and WM volumes that we observed in neonates appear to be consistent with findings from several other neuroimaging studies in DS ranging from childhood to adolescence (Hamner et al. 2018). These studies have reported significantly enlarged absolute and WBV-adjusted parietal GM and WM, as well as temporal GM and WM volumes (Pinter et al. 2001b; Kates et al. 2002; Lee et al. 2016). Conversely, adjusted frontal GM was not significantly different, whereas adjusted frontal WM, and absolute occipital WM volume, were reported to be significantly smaller than control (Kates et al. 2002; Lee et al. 2016).

Regional differences in cortical GM and WM proportions may be linked to a brachycephalic cranial morphology with a flat occiput commonly observed in DS (Rodrigues et al. 2019). Brachycephaly was noted in our previous study in fetuses with DS, whereby linear measurements for occipitofrontal diameter (OFD) and head circumference (HC) were significantly smaller in DS after 28 GW, together with a significantly reduced WBV (Patkee et al. 2020). Furthermore, post-mortem biometric examinations have also identified brachycephaly in fetuses with DS as early as 15 GW, as evidenced by a larger biparietal diameter (BPD) width and BPD to HC ratio (Guihard-Costa et al. 2006). In future, we hope to conduct a more systematic correlation of linear measures (evidencing brachycephaly) and volumetric results in our neonatal cohort.

### Regional differences in relative cortical GM volumes may be linked to altered cortical thickness, surface area and folding observed in DS

Regional differences in cortical GM proportions may be linked to altered cortical thickness, surface area, and folding, which have all been reported in DS (Lee et al. 2016; Levman et al. 2019; Yun et al. 2021). We observed that the frontal and occipital GM segments were not significantly different from control, whereas the temporal and parietal GM segments were significantly enlarged in proportion. Reduced cortical folding (i.e. lower average sulcal depth and gyrification index) has been observed as early as 28 GW in fetuses with DS (Yun et al. 2021). In children with DS (from 0 to 5 years), cortical thickness was increased, whereas its variability was decreased, indicating an abnormal maturation of GM in several brain regions (Levman et al. 2019). In youth with DS (from 5 to 24 years), cortical thickness was increased throughout much of the frontal, superior parietal and occipital lobes, whereas surface area was reduced in the frontal and temporal lobes (Lee et al. 2016). As such, it is likely that regional alterations in cortical thickness, surface area and folding may underlie the regional differences in relative cortical GM volumes that we have observed with neonates in DS. In future, we hope to study both volumetric and morphometric data in tandem to better understand regional changes to the cortical GM in neonates with DS.

Lastly, from a histological perspective, post-mortem analyses have reported a reduction in total neuronal number, as well as a delayed and disorganized lamination of the cortical GM in DS (Wisniewski et al. 1984; Schmidt-Sidor et al. 1990; Wisniewski 1990; Golden and Hyman 1994). More specifically, Guidi et al. identified decreased neurogenesis and increased cell death in certain GM regions, such as the hippocampus (Guidi et al. 2008), the fusiform gyrus and the inferior temporal gyrus (Guidi et al. 2018) from 17 to 21 GW. As such, greatly reduced neuronal cell proliferation, and increased cell death, may account for the reduced absolute cortical GM volumes noted in neonates with DS.

### Proportionally reduced WM volumes may be associated with altered fetal WM development in DS

With regards to the cerebral WM, we found that the cingulate, frontal, insular, and occipital WM segments were significantly reduced in relative volume. These findings may be supported by a growing body of post-mortem research evidencing aberrant fetal WM development in DS. A postnatal delay in myelination has been noted in DS since the 1980s (Wisniewski and Schmidt-Sidor 1989; Wisniewski 1990). More recently, Olmos-serrano et al. conducted a multi-region transcriptomic analysis of DS and euploid brain tissue spanning from the mid-fetal stage to adulthood, in which they uncovered the co-dysregulation of genes associated with oligodendrocyte differentiation and myelination (Olmos-Serrano et al. 2016). Several other studies have reported glial disturbances in the developing brain in DS, including significantly reduced radial glial progenitors (Baburamani et al. 2020), and an imbalance in astro- and oligodendroglial cells (Zdaniuk et al. 2011; Kanaumi et al. 2013; Reiche et al. 2019, 2021). This glial imbalance may be due to the altered expression of transcription factors that are essential for oligodendroglial differentiation (Olmos-Serrano et al. 2016; Reiche et al. 2021; Klein et al. 2022). Thus, it is possible that the cellular, axonal, and, perhaps, extracellular matrix compositions of the fetal WM are altered in DS, giving rise to the significantly reduced relative volumes observed in this study.

### The impact of CHDs on neonatal brain volumes in DS

In our study, the occipital WM displayed a significant difference in age-related volume between CHD+ and CHD− neonates with DS. Occipital WM volumes were significantly smaller in CHD+ neonates by later ages at scan (from approximately 40 weeks PMA). Interestingly, reduced occipital regional volume has been associated with later visual difficulties in preterm infants without DS (Shah et al. 2006). It is widely recognized that children with DS have a broad range and high prevalence of visual deficits (Wilton et al. 2021). Thus, in future, it would be of great interest to associate neonatal occipital WM volume with visual outcomes in CHD+ and CHD− children with DS.

We also observed other age-related volumetric differences between CHD+ and CHD− neonates, although these were not statistically significant after multiple comparison correction, most likely due to the small subgroup sizes and low statistical power in our study. Overall, WBV tended to be smaller in CHD+ neonates compared with CHD− neonates by later ages at scan. A high proportion (three out of four neonates) with extreme negative deviations in WBV were scanned at later PMA, had several cardiac defects, and presented low oxygen saturation scores at the time of their neonatal scan. All CHD+ neonates in this study were scanned prior to any cardiac surgery or intervention, although most required surgery within 6 months of life (see Supplementary Table S2 for details). Interestingly, body weight at scan, corrected for individual sex and age, was not different between CHD+ and CHD− subgroups, implying that there may be impaired brain growth, over body growth, in neonates with CHD during this neonatal period.

Taken together, our findings may indicate that there is a baseline brain phenotype in neonates with DS, which is further altered in the presence of an associated CHD. We hypothesize that the volumetric differences and trends observed in CHD+ neonates may be due to compromised cardiac function, and reduced cerebral oxygenation in early postnatal life (Sun et al. 2015; Kelly et al. 2017, 2019). It is also possible that there may be an additional genetic mechanism present in neonates with CHD, as noted in detailed gene mapping experiments using the Dp1Tyb mouse model of DS (Lana-Elola et al. 2016). In order to better understand the underlying mechanisms affecting neonates with CHD, in future, we could utilize phase contrast angiography to assess cerebral oxygen delivery (Bonthrone et al. 2021) and longitudinal follow-up scanning of the same infants over time. Earlier interventions to improve cerebral oxygen delivery may help promote early brain growth and improve developmental outcomes in DS infants with CHD (Visootsak et al. 2011, 2013, 2016).

### Limitations

The primary limitation of this study was the small sample size of neonates with DS, particularly for the clinically defined subgroups (e.g. CHD+ vs CHD−). In the UK, the estimated live birth prevalence for DS was approximately 1.16 in 1,000 in 2018, in line with the rest of Europe (de Graaf et al. 2021), whereas an estimated 85.2% of antenatal diagnoses were terminated that year (DSMIG, www.dsmig.org.uk). Our neonatal recruitment is primarily conducted through one site, St Thomas’ Hospital/Evelina Children’s Hospital (London), and thus, multi-site recruitment within the UK, or globally, would be needed to significantly increase sample size. In future, retrospective data harmonization may also be possible, depending on the type of analyses sought. Nevertheless, to the best of our knowledge, our cohort represents the largest dataset of neonates with DS scanned in a prospective study, which is unaffected by variability in acquisition parameters and in line with a robust neonatal control population.

In our study, participants were each imaged once as a neonate (up to < 46 weeks PMA), and we do not have any follow-up scans during the neonatal period. In future, it could be particularly beneficial to conduct follow-up imaging to monitor the continued impact of CHD on the neonatal brain. Although we have used a well-validated segmentation pipeline optimized for the neonatal brain (Makropoulos et al. 2014, 2018), some current limitations include the lack of automated segmentation for the *cavum septum pellucidum*, the third and fourth ventricles, and the cerebellar sub-structures. Structural MRI, and volumetric quantification, cannot tell us detailed information about underlying microstructure and neurocircuitry. In future, we hope to associate data derived from diffusion MRI to complement our volumetric findings. Lastly, although these have not been discussed in this paper, neurodevelopmental outcomes are being collected from participants in our study. These data will be essential to understand if any features of the neonatal brain may serve as early biomarkers for later developmental outcomes in DS.

## Conclusion

In conclusion, we conducted the first comprehensive volumetric phenotyping of the neonatal brain in DS. We have demonstrated significant volumetric groupwise differences across multiple brain segments between neonates with DS and a robust control cohort. For the first time, we have also identified age-related volumetric differences between CHD+ and CHD− neonates with DS.

We observed several volumetric features of the neonatal brain, which appear to follow a developmental continuum in DS, including a reduced absolute WBV; relatively reduced frontal and occipital lobar volumes, in contrast with relatively enlarged temporal and parietal lobar volumes; relatively enlarged deep GM structures, particularly the lentiform nuclei; a decreased cerebellar volume; and a tendency for enlargement of the lateral ventricles, amongst other features.

There is a relative scarcity of knowledge about neonatal brain development in DS and how this may be associated with later neurodevelopmental outcomes. Currently, there are no pediatric longitudinal neuroimaging investigations in DS, starting from the very earliest time points (e.g. fetal and/or neonatal), which greatly impedes our understanding of the developmental continuum of neuroanatomical and cognitive parameters. In future, this field of research could greatly benefit from long-term longitudinal imaging and larger sample sizes, which could be delivered by collaborative multi-site investigations. Although life expectancy of individuals with DS has greatly improved over the last few decades (De Graaf et al. 2017; Antonarakis et al. 2020), early interventions may be essential to help improve outcomes and long-term quality of life in DS.

## Supplementary Material

CC_Final_Supplementary_bhad171
