# Supplementary material for: Comprehensive volumetric phenotyping of the neonatal brain in Down syndrome
Source: Cereb Cortex. 2023 May 26;33(14):8921–41. doi: 10.1093/cercor/bhad171 (PMC10350827; doi:10.1093/cercor/bhad171)
Supplement: CC_Final_Supplementary_bhad171 [file cc_final_supplementary_bhad171.docx]

**SUPPLEMENTARY INFORMATION**

**Index**

**Supplementary Figures**

- **Figure S1:** GPR plots for all tissue segments and structures.
- **Figure S2:** Volumetric brain development in the control group from 32 to < 46 weeks PMA.
- **Figure S3:** DS and control simple linear regressions by brain segment (using absolute volume z-scores).
- **Figure S4:** CHD+ and CHD- simple linear regressions by brain segment (using absolute volume z-scores).
- **Figure S5:** Covariation analysis of absolute volume z-scores against whole brain volume (WBV) z-scores using linear and median regression.

**Supplementary Tables**

- **Table S1**: Demographic, weight, and head circumference data for neonates with DS.
- **Table S2:** Congenital heart defects (CHD) in neonates with DS.
- **Table S3:** Additional clinical details for neonates with DS.
- **Table S4:** Volumetric brain development in control group from 32 to < 46 weeks PMA.
- **Table S5:** Table of results for the extra sum-of-squares F tests comparing DS and control simple linear regressions (absolute volume z-scores against PMA at scan).
- **Table S6:** Table of results for Spearman’s rank correlation tests (absolute volume z-scores against PMA at scan).
- **Table S7:** Group comparison of whole brain volume-adjusted medians (derived from the covariation analysis) between DS and control neonates.
- **Table S8:** Group comparison of absolute volume z-scores between CHD+ and CHD- neonates.
- **Table S9:** Table of results for the extra sum-of-squares F tests comparing simple linear regressions for CHD+ vs. CHD- neonates with DS (using absolute volume z-scores).
- **Table S10:** Table of results for Spearman’s rank correlation tests for CHD+ and CHD- neonates with DS (using absolute volume z-scores).

**SUPPLEMENTARY FIGURES**

**Figure S1:** GPR plots for all tissue segments and structures.

**
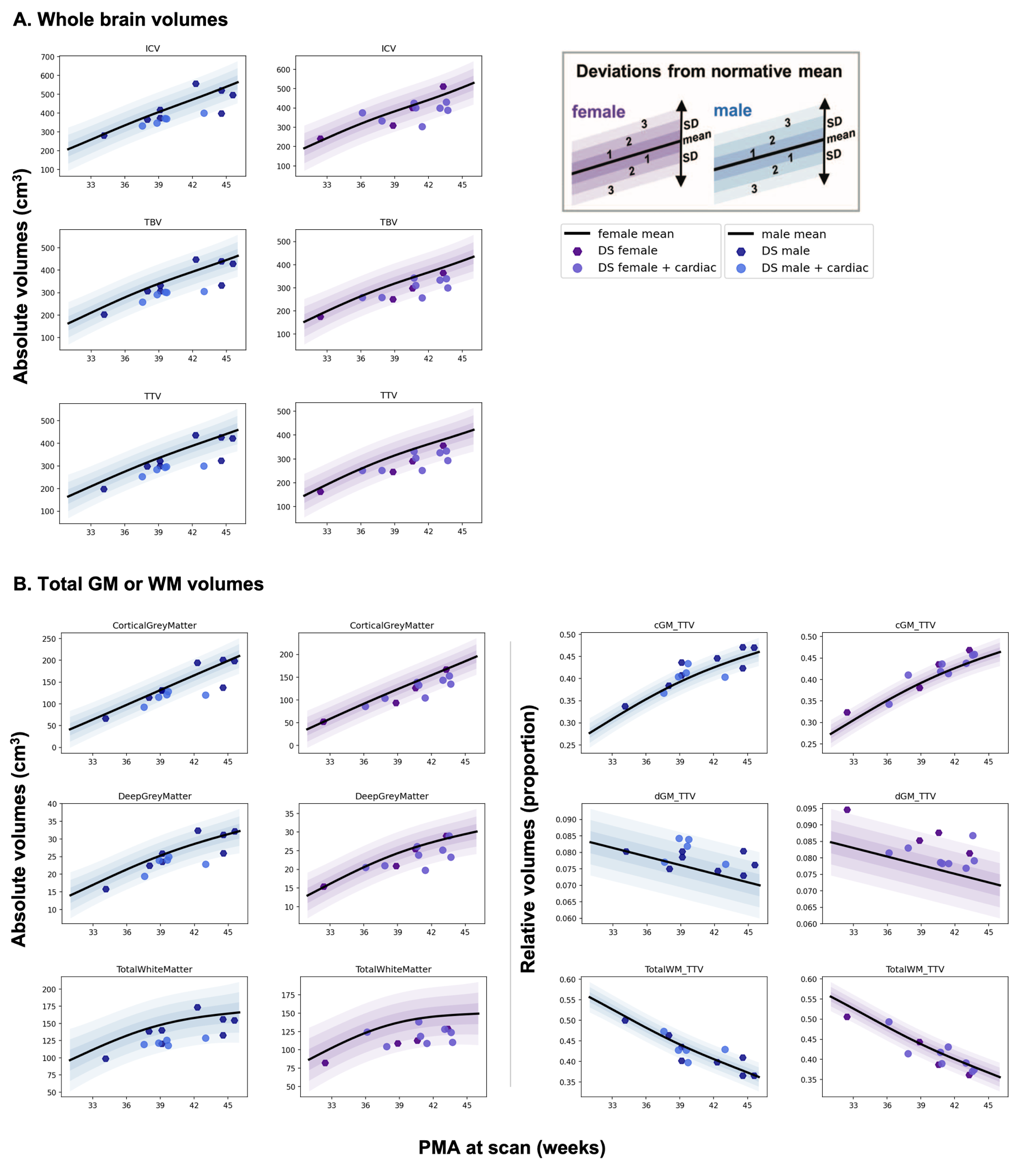
**

**
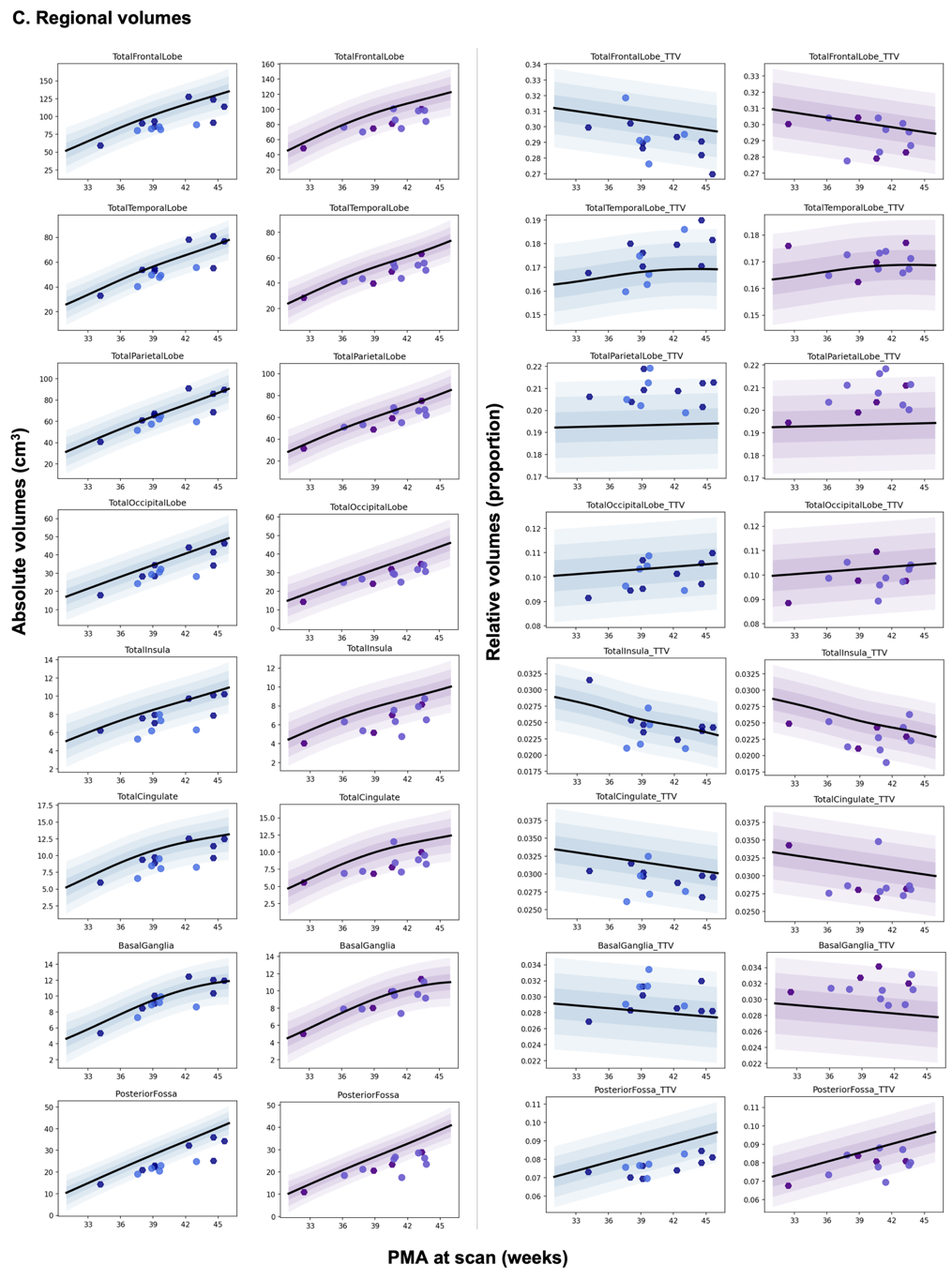
**

**
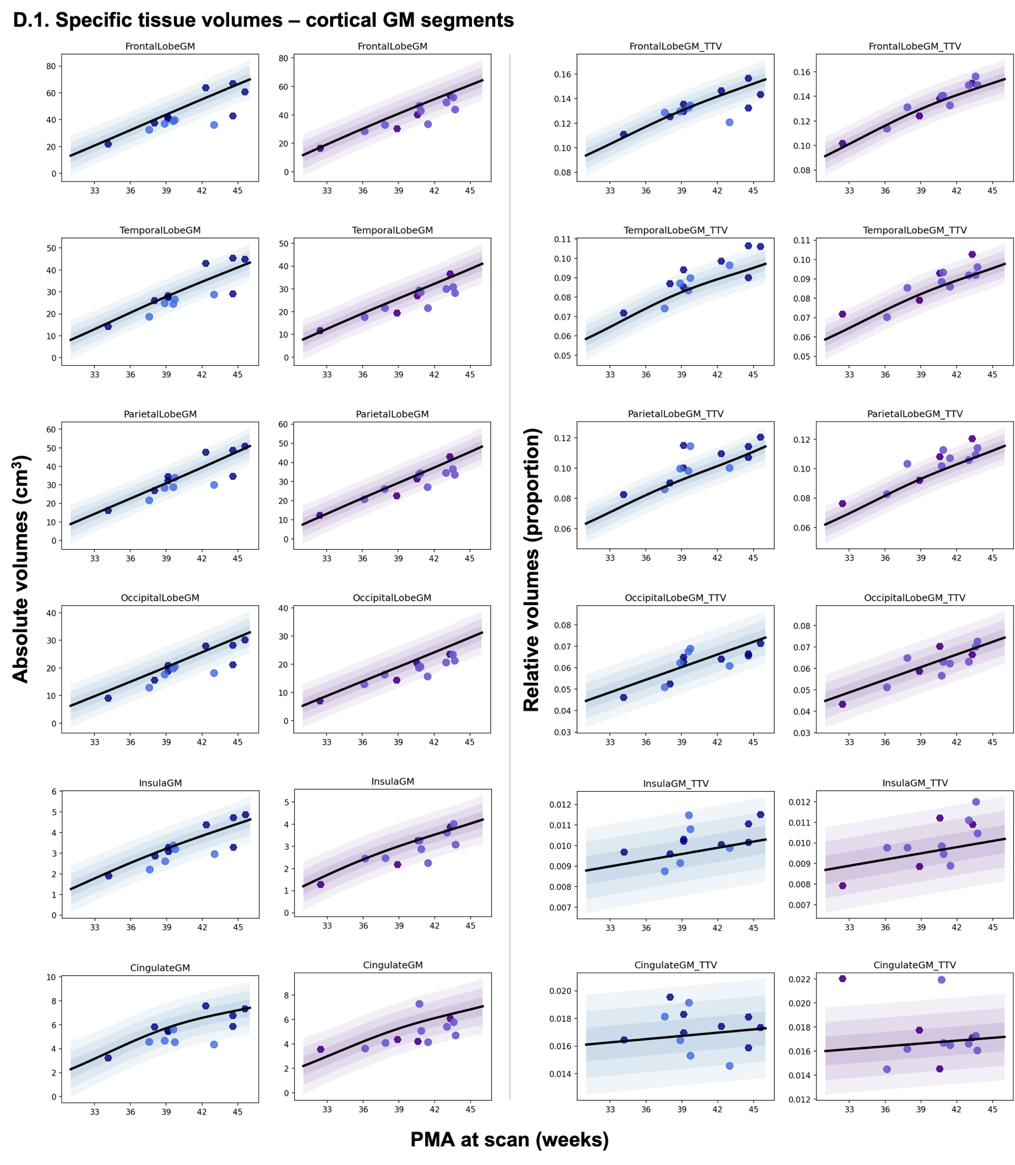
**

**
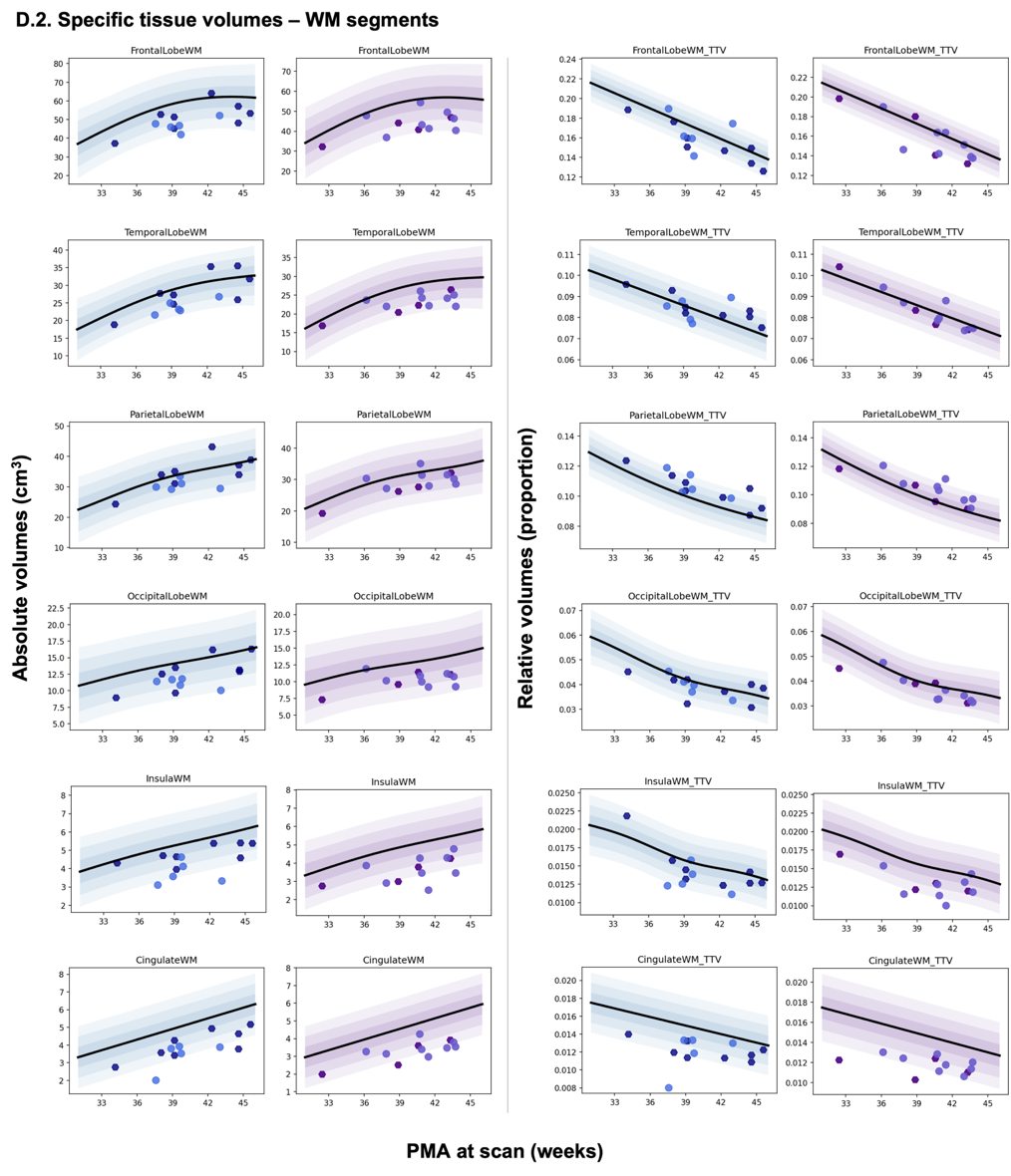
**


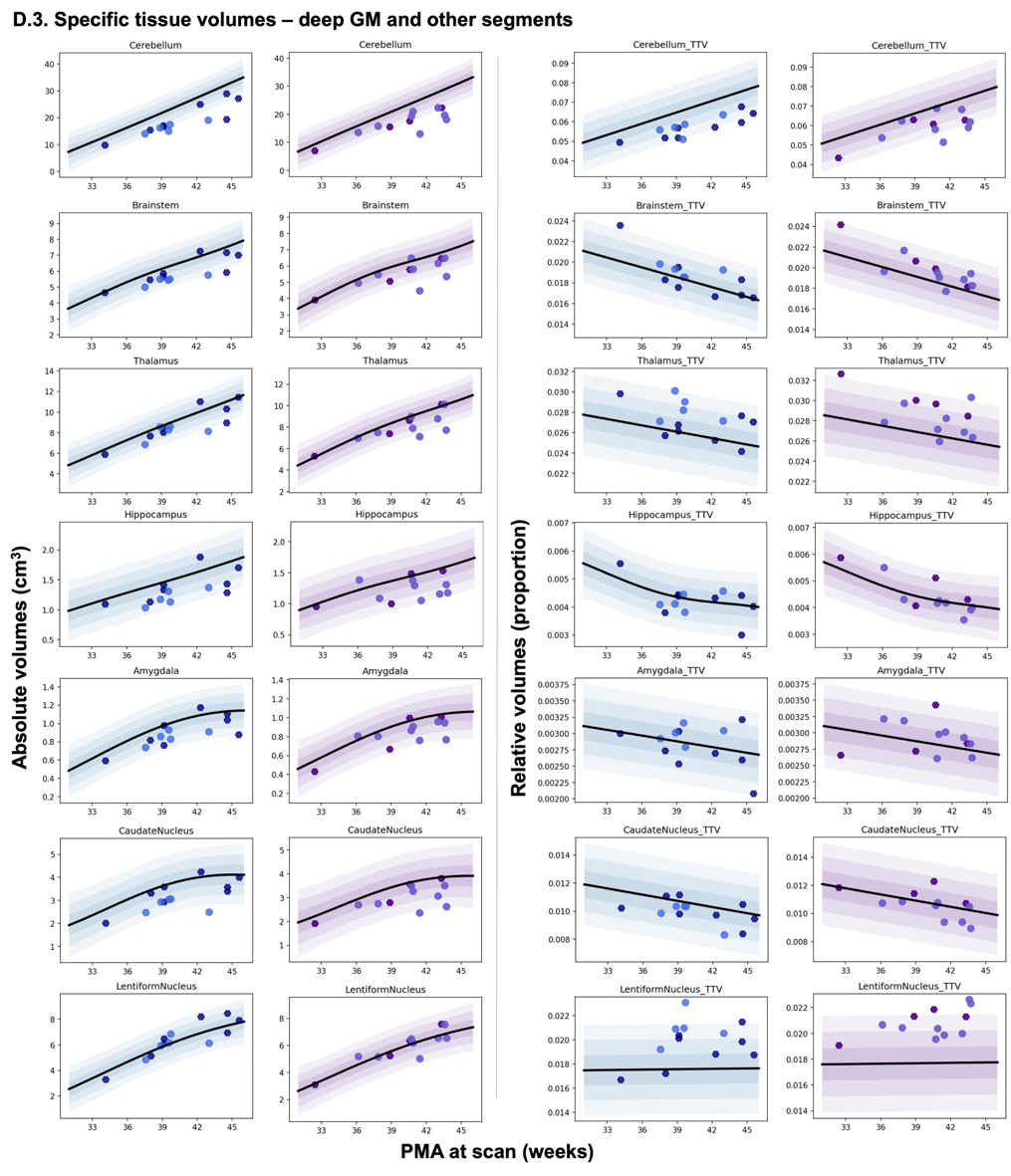


**Figure S2:** Volumetric brain development in the control group from 32 to < 46 weeks PMA.

Scatter plots of absolute (in cm^3^) or relative volumes (proportional) from 32 to < 46 weeks PMA for n = 493 preterm to term-born control neonates (females and males consolidated). Data was fitted with a Gaussian curve (bolded curve) and 95% confidence intervals (dotted lines). Plots for **A)** whole brain volumes (in cm^3^), **B)** main tissue classes (in cm^3^) and **C)** main tissue classes (in relative volume), **D)** cortical GM segments (in cm^3^), **E)** WM segments (in cm^3^), **F)** Deep GM and other segments (in cm^3^), **G)** cortical GM segments (in relative volume), **H)** WM segments (in relative volume) and **I)** Deep GM and other segments (in relative volume). Refer to Table S4 for more information.

**
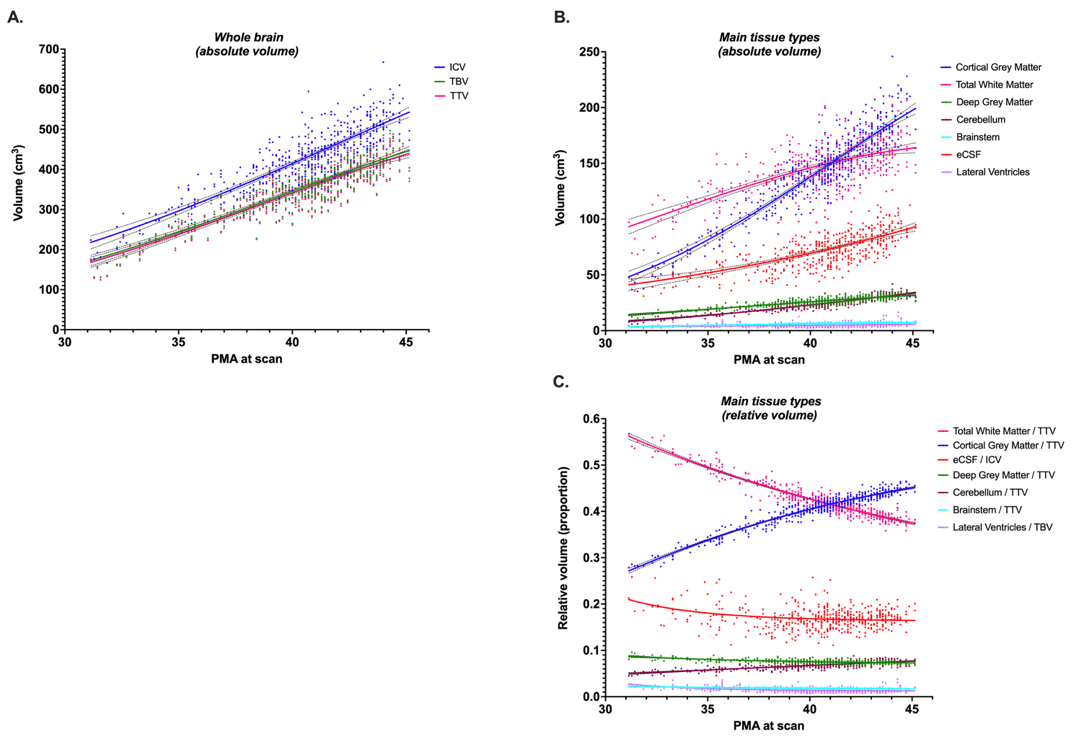
**

**
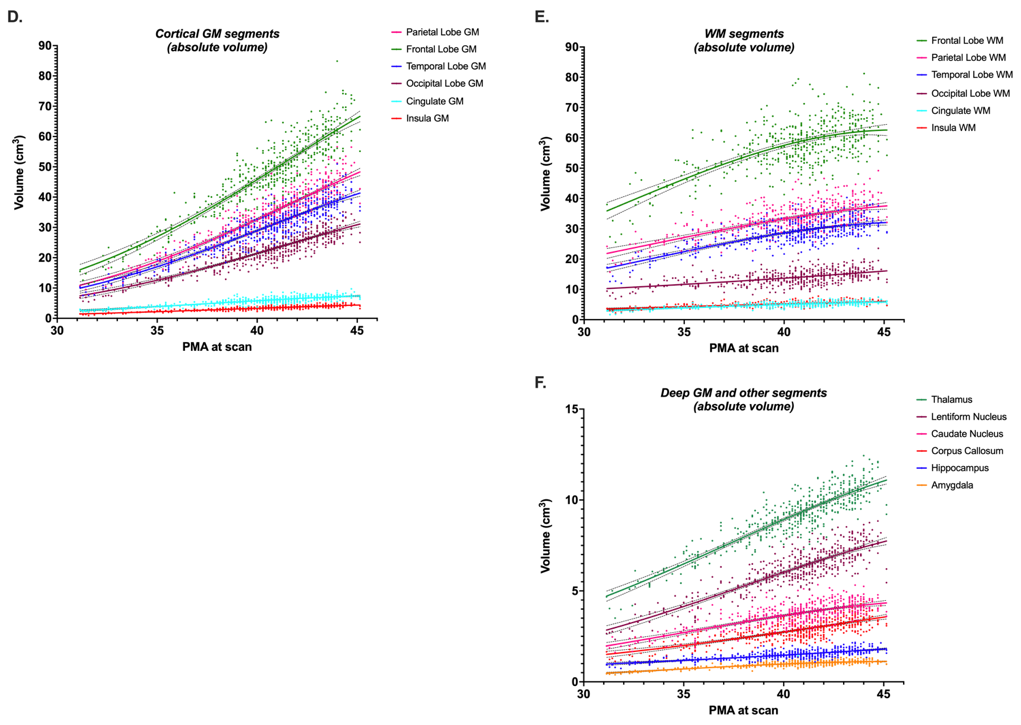
**

**
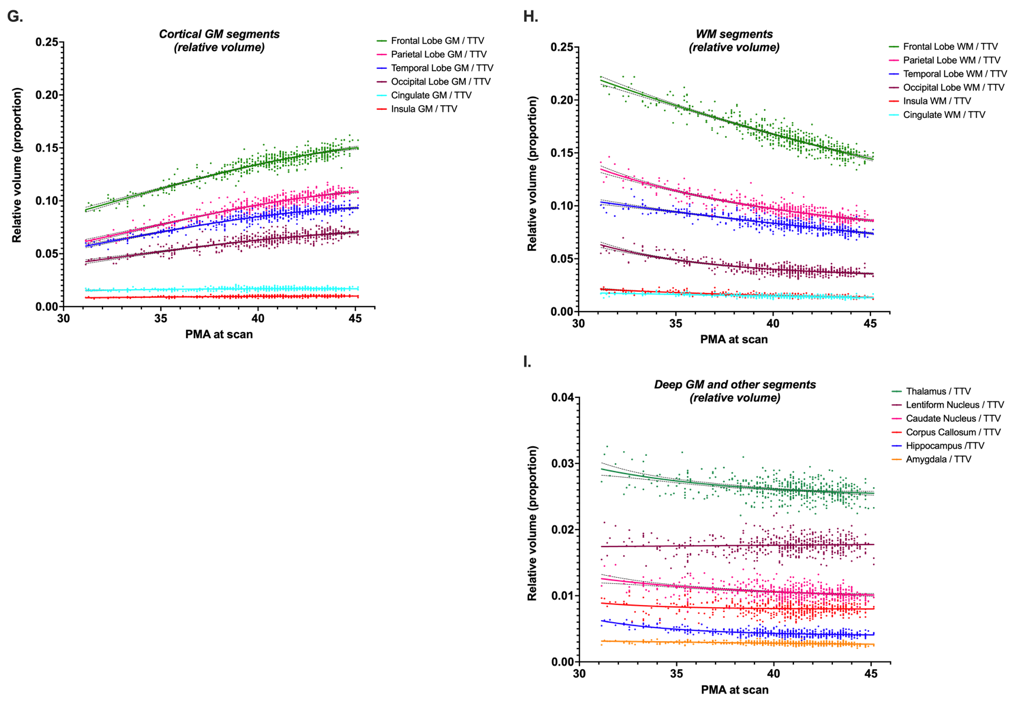
**

**Figure S3:** DS and control simple linear regressions by brain segment (using absolute volume z-scores).

Simple linear regression plots of absolute volume z-scores against PMA at scan from 32 to 46 weeks PMA for **A.** whole brain, **B.** cortical GM segments, **C.** WM segments, **D.** deep GM and other segments. Main tissue classes can be found in Figure 5 of main text. Dots for individual control neonates (n = 493, females and males consolidated) appear in blue, and linear regressions appear as flat blue lines at z = 0 with 95% confidence intervals. Dots for individual neonates with DS (n = 25, females and males) appear in red, and linear regressions appear as red lines with 95% confidence intervals. For additional information, dotted black lines indicate the median regression. Parameters for DS and control linear regressions appear in the top left (i.e., equation, R^2^, adjusted R^2^, F and P-value), whilst parameters for the median regressions appear in the bottom right of each graph (i.e., equation and AIC). A table of results for F-tests can be found in Table S5 and Spearman’s correlation in Table S6.

**
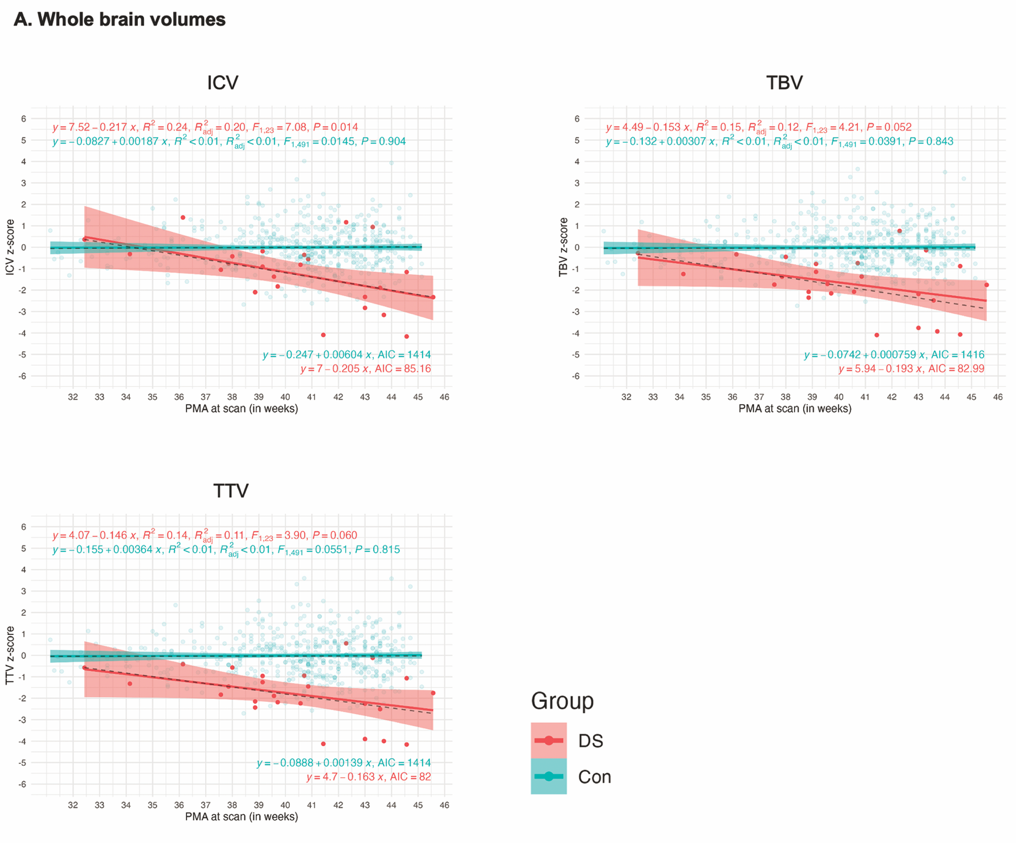
**

**(See next pages for B, C, D).**


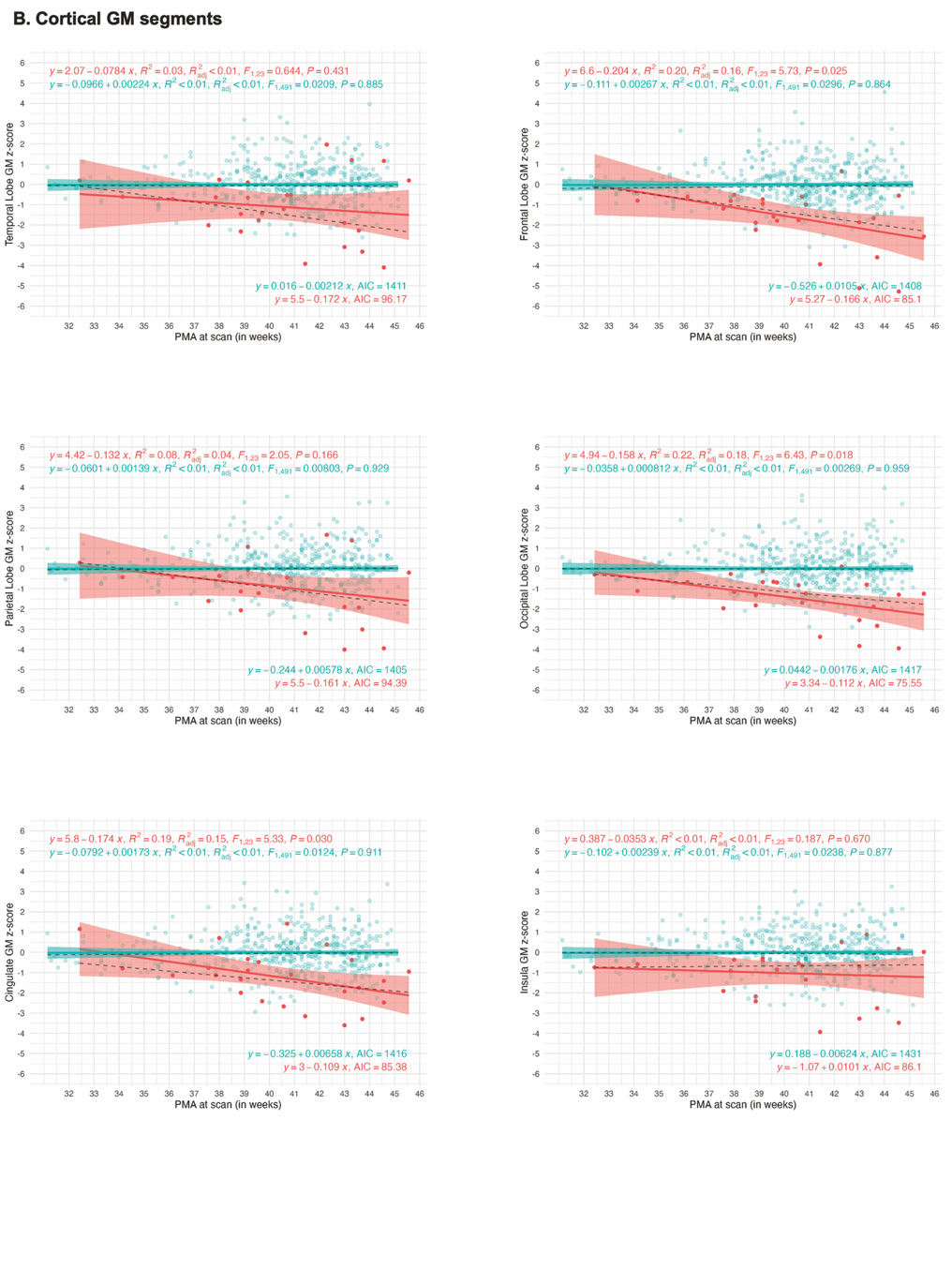


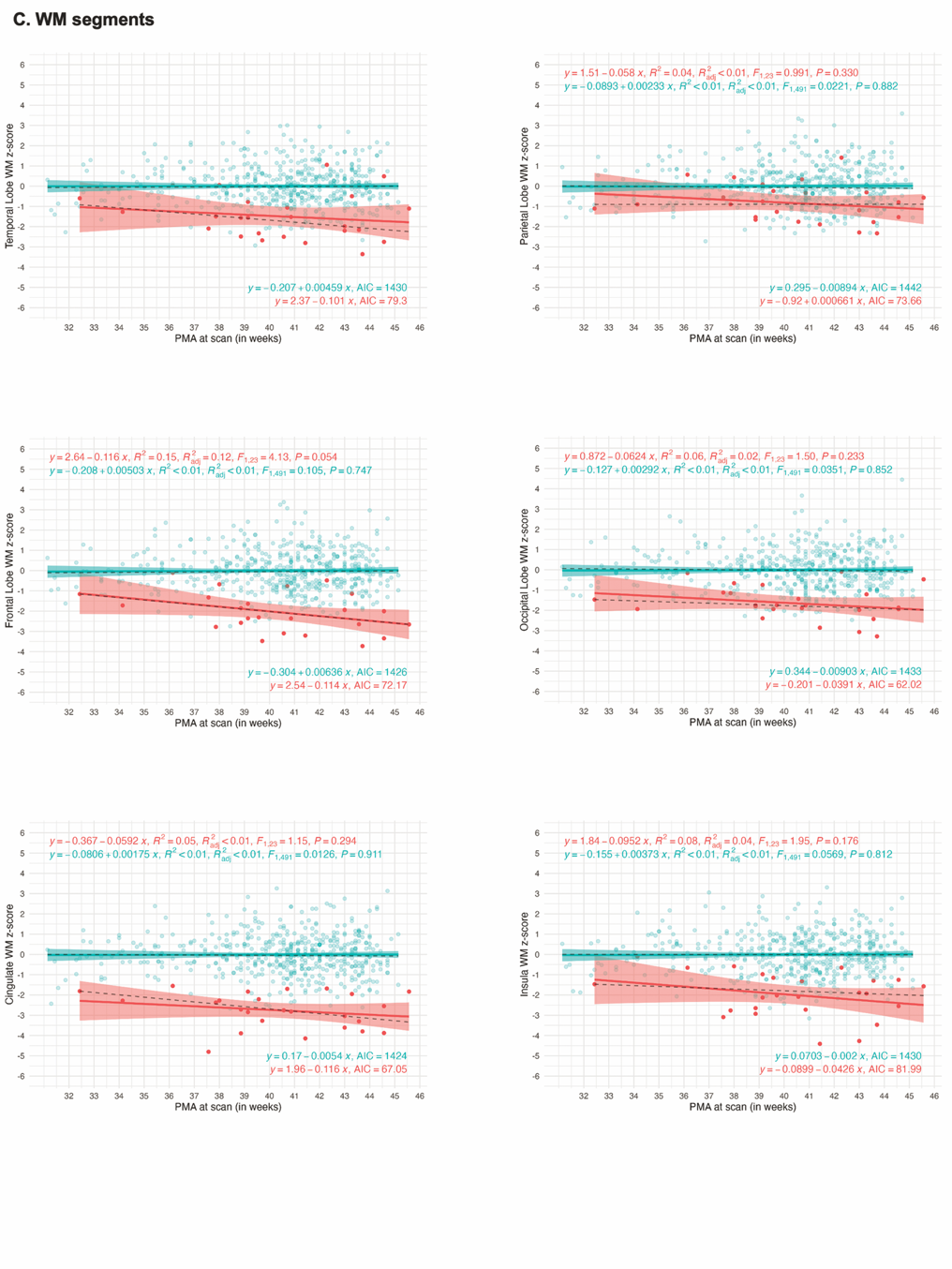


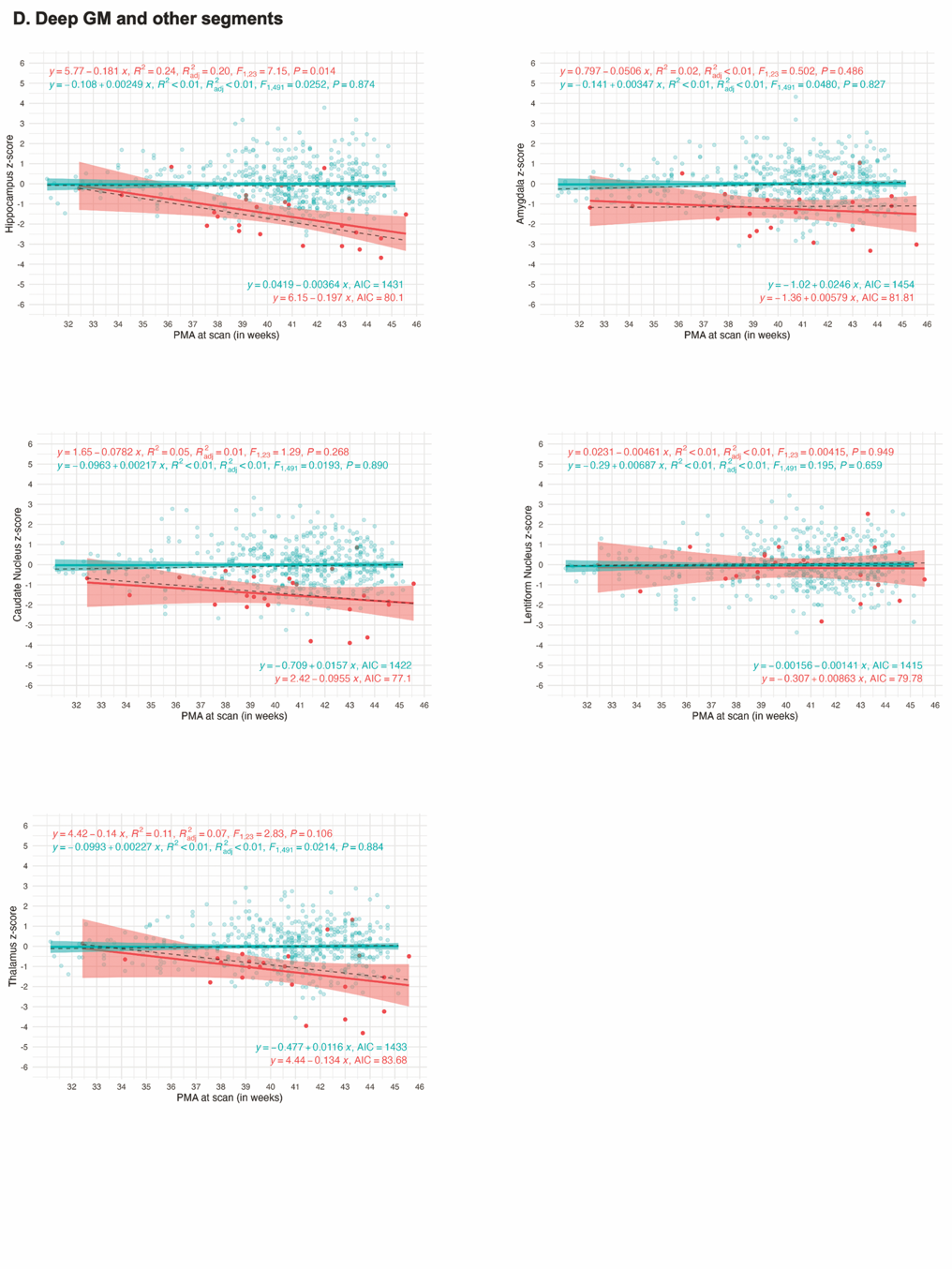


**Figure S4:** CHD+ and CHD- simple linear regressions by brain segment (using absolute volume z-scores).

Simple linear regression plots of absolute volume z-scores against PMA at scan from 32 to 46 weeks PMA for **A.** whole brain volumes, **B.** cortical GM segments, **C.** WM segments, **D.** deep GM and other segments. CHD+ neonates with DS (n = 13) appear in blue, whilst CHD- neonates with DS (n = 12) appear in red. Linear regressions appear as coloured lines with 95% confidence intervals, whilst for additional information dotted black lines indicate the median regression. Parameters for the linear regressions appear in the top left (i.e., equation, R^2^, adjusted R^2^, F statistic and P-value), whilst parameters for the median regressions appear in the bottom right of each graph (i.e., equation and AIC). Plots for main tissue classes can be found in Figure 6 of main text. A table of results for F-tests can be found in Table S9 and Spearman’s correlation in Table S10.


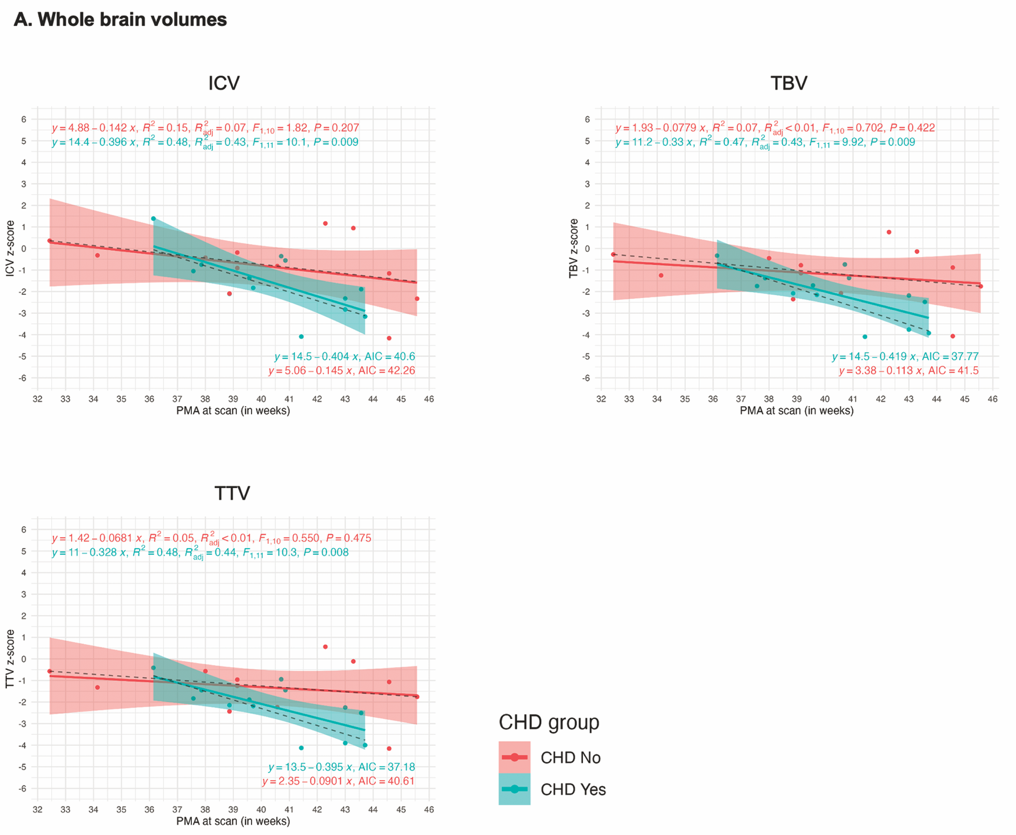


**(See next pages for B, C, D).**


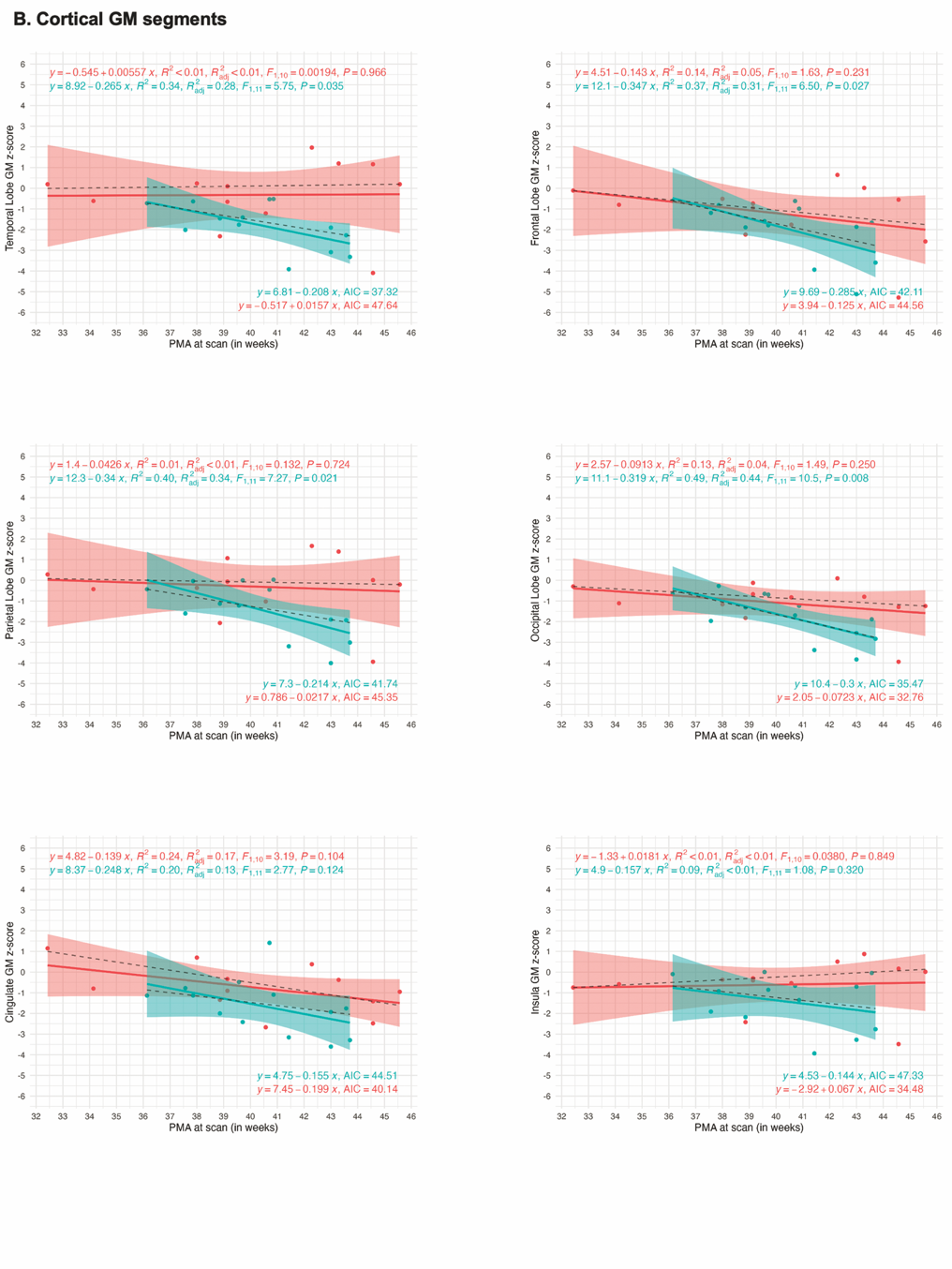


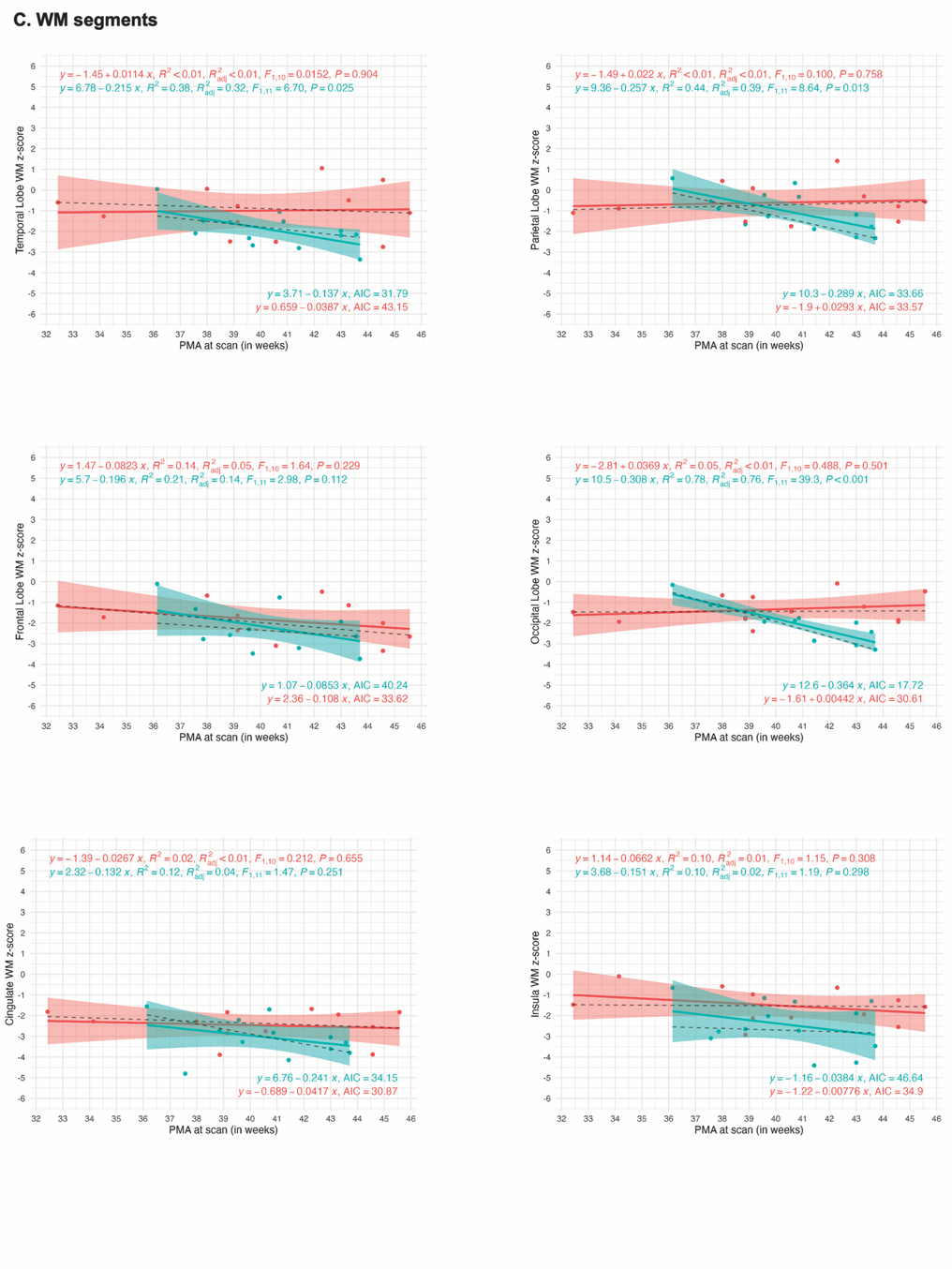


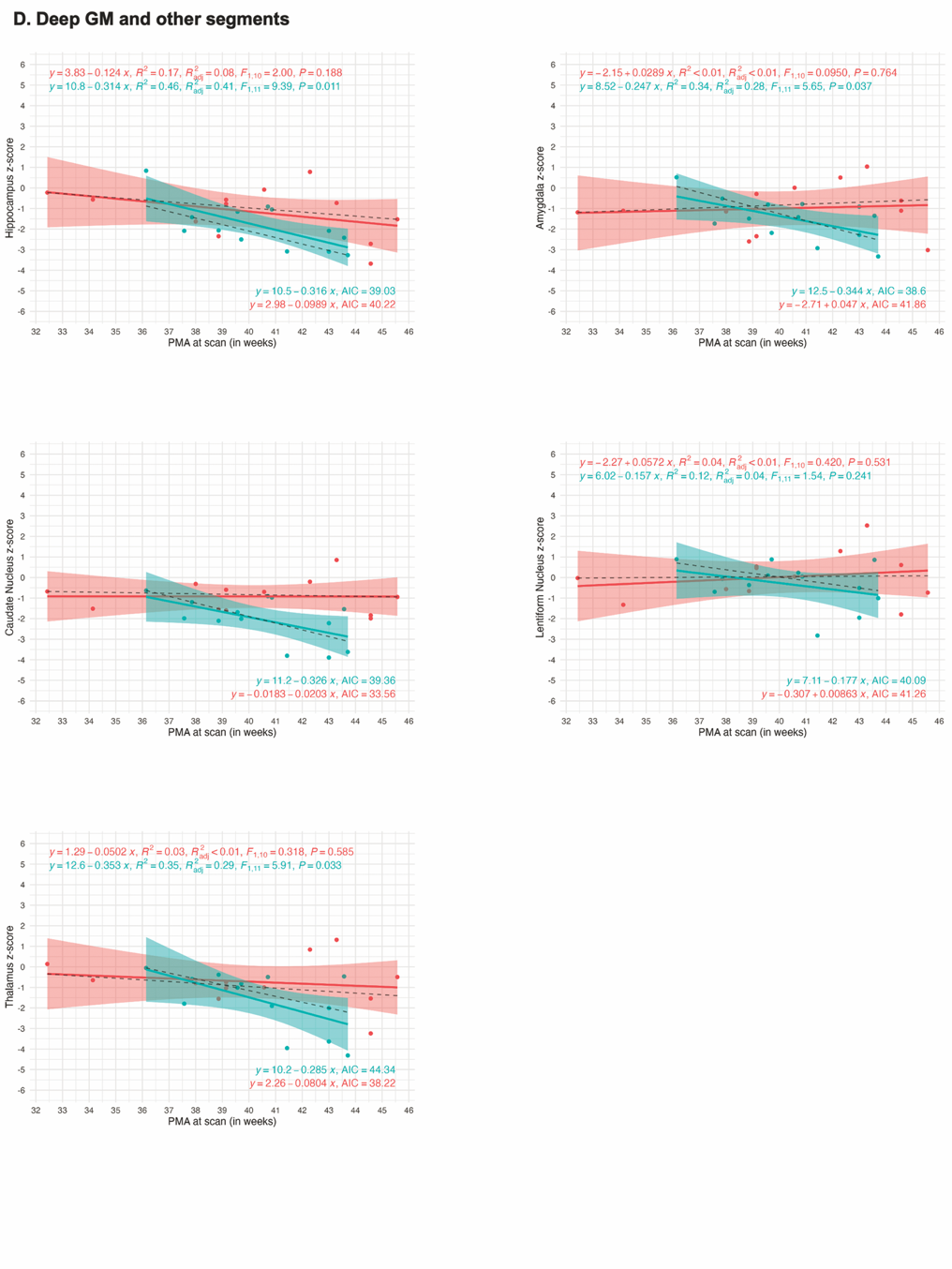


**Figure S5:** Covariation analysis of absolute volume z-scores against whole brain volume z-scores using linear and median regression.

Plots of absolute volume z-scores against WBV z-scores (i.e., ICV, TBV or TTV). Plots for **A.** cortical GM segments, **B.** WM segments, **C.** deep GM and other segments. Dots for control neonates (n = 493, females and males) appear in blue, with coloured linear regressions and coloured 95% confidence intervals. Dots for neonates with DS (n = 25, females and males) appear in red, with coloured linear regressions and coloured 95% confidence intervals. Additionally, dotted black lines indicate quartile regressions (i.e., 1st quartile, median, and 3rd quartile). Parameters for the linear regressions appear in the top left (i.e., equation, R^2^, adjusted R^2^, F and P-value), whilst parameters for the median regressions appear in the bottom right of each graph (i.e., equation and AIC). Plots for main tissue classes can be found in main text Figure 5. A table comparing WBV-adjusted median z-scores in DS vs control groups can be found in Table S7.


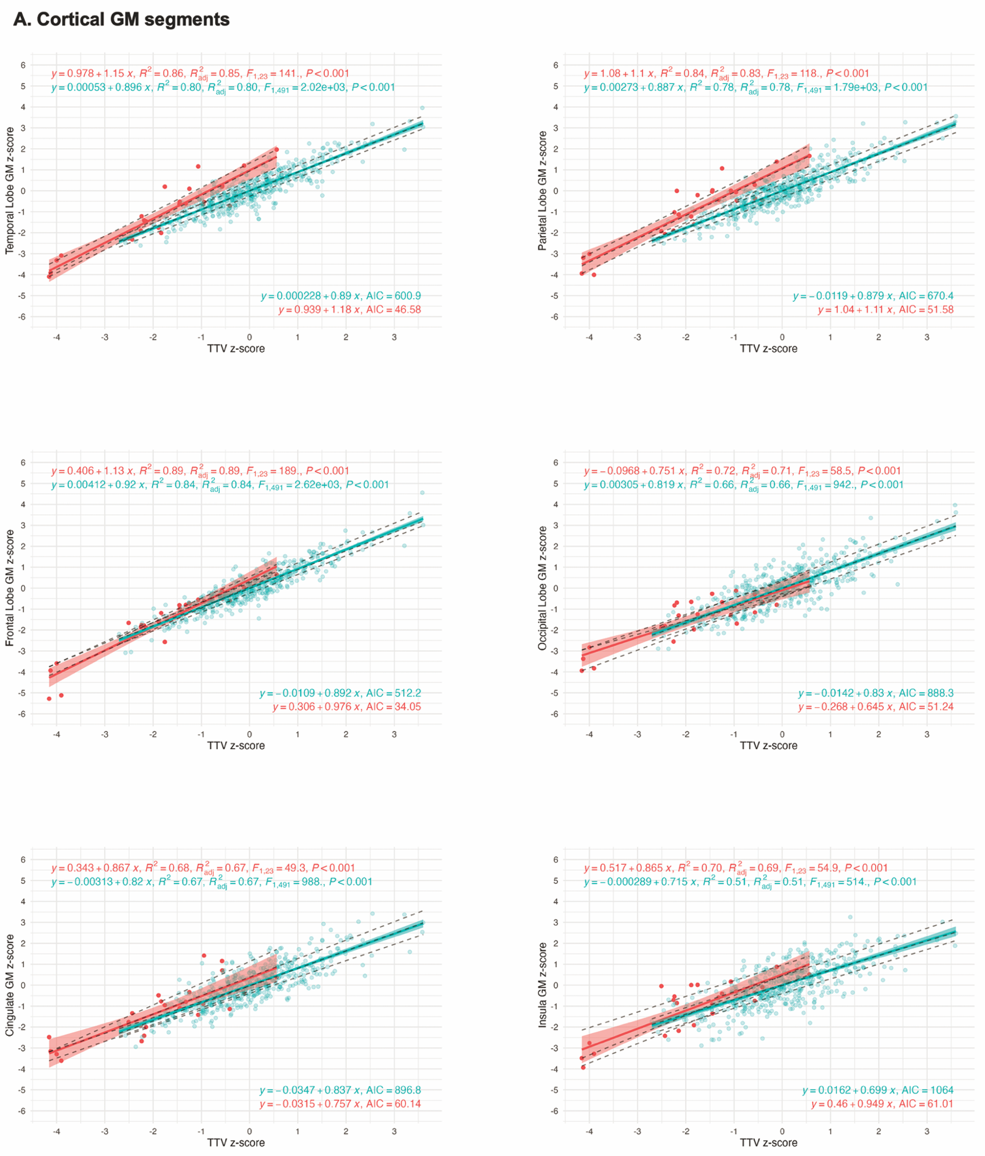


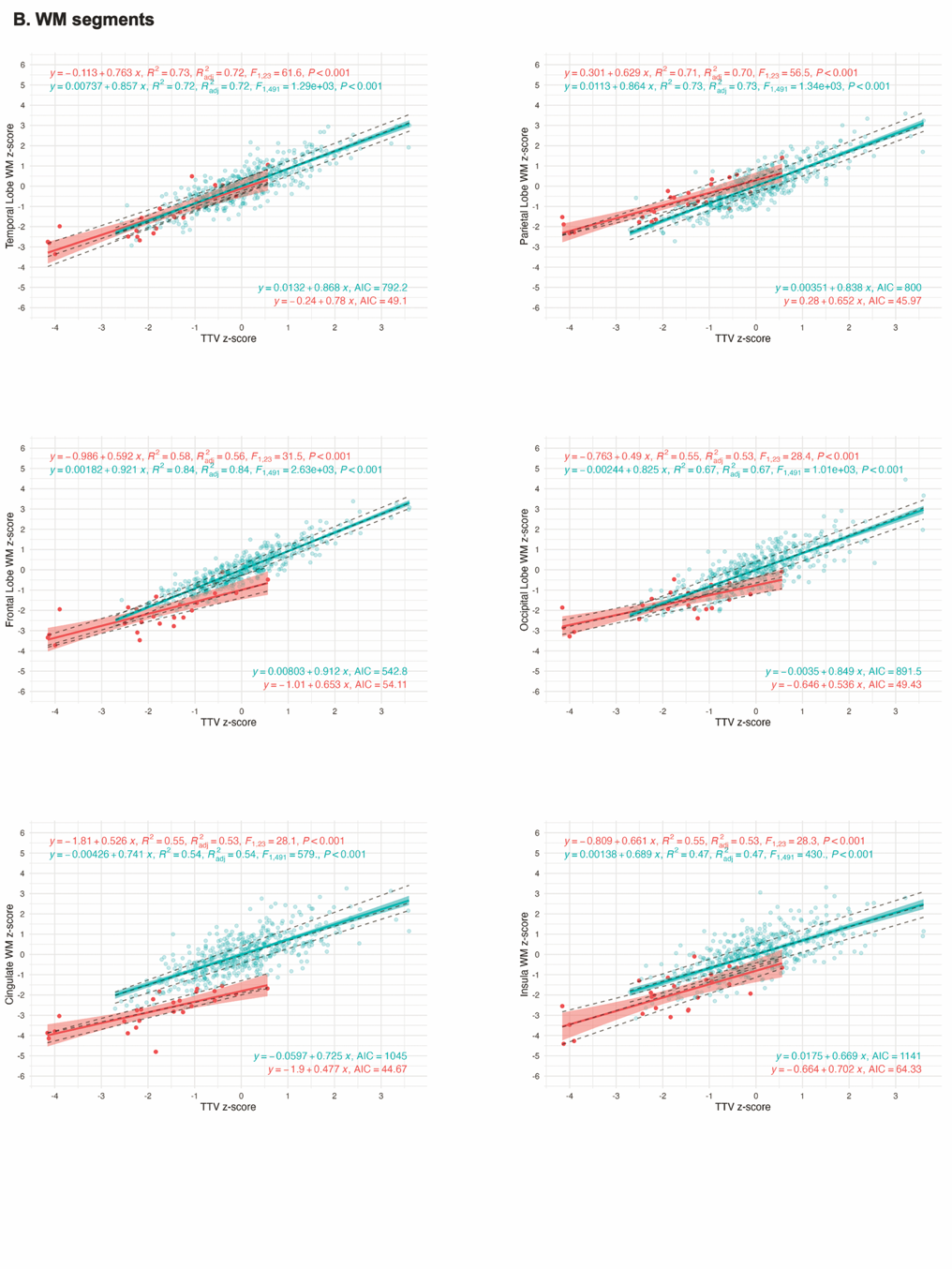


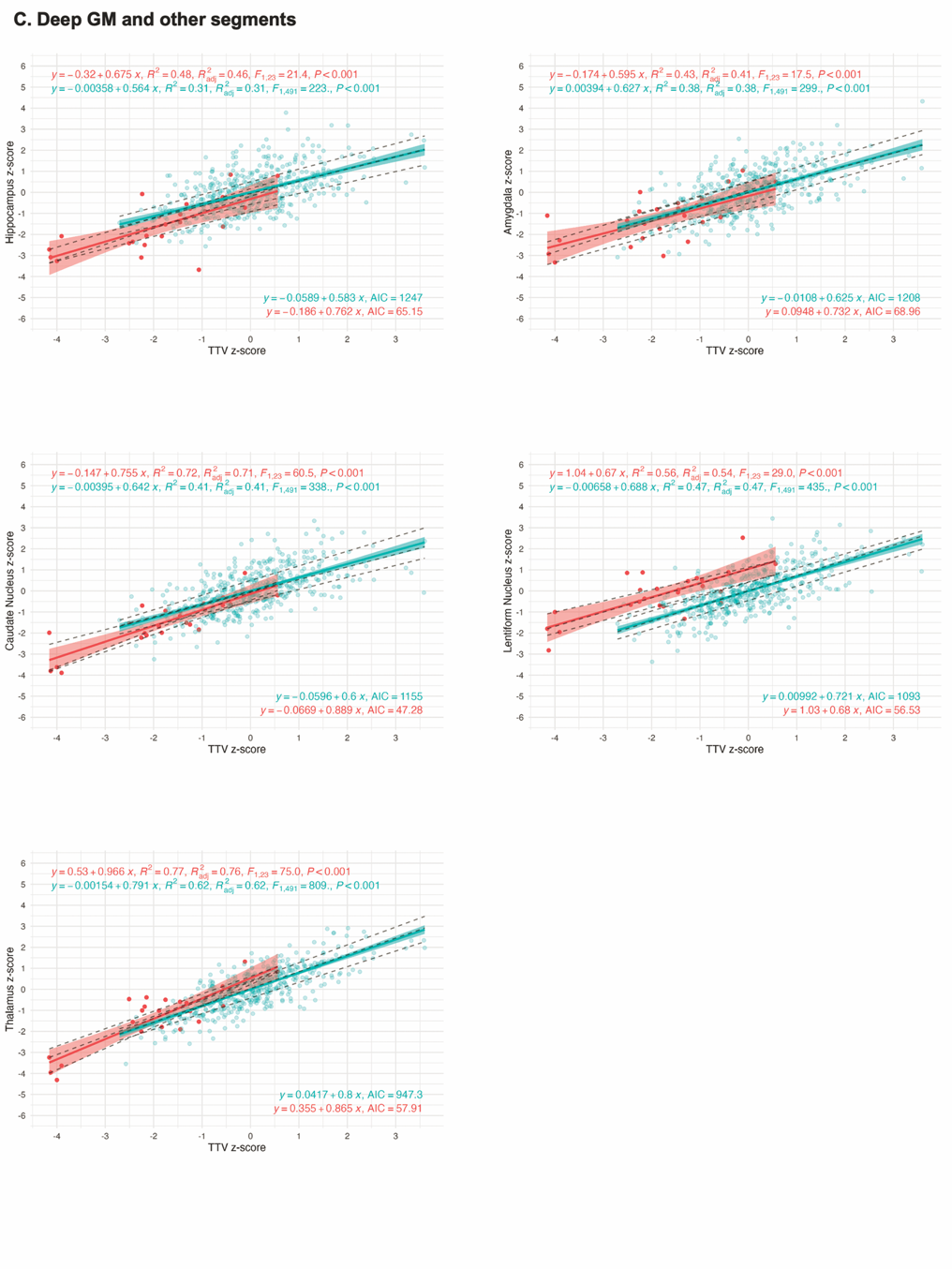


**SUPPLEMENTARY TABLES**

**Table S1:** Demographic, weight, and head circumference (HC) data for neonates with DS.

Data extracted from clinical records on day of scan. HC at birth was missing for 3 neonates with DS. Z-scores were calculated based on the RCPCH UK-WHO growth charts using the ‘*childsds*’ package (v0.7.6) in R.

| **ID** | **Sex** | **Preterm/Term category** | **GA at birth (weeks)** | **PMA at scan (weeks)** | **Age from birth (weeks)** | **Birth Weight (kg)** | **Birth Weight (z)** | **Birth**  **HC (cm)** | **Birth**  **HC (z)** | **Scan Weight (kg)** | **Scan Weight (z)** | **Scan**  **HC (cm)** | **Scan**  **HC (z)** |
| --- | --- | --- | --- | --- | --- | --- | --- | --- | --- | --- | --- | --- | --- |
| **NT21_6** | F | Early Preterm | 31.43 | 43.29 | 11.86 | 1.56 | -0.15 | 28.0 | -0.91 | 3.45 | -0.89 | 36.8 | 0.70 |
| **NT21_8** | F | Late Preterm | 36.43 | 43.00 | 6.57 | 2.28 | -0.99 |  |  | 3.31 | -1.03 | 33.4 | -2.09 |
| **NT21_9** | M | Late Preterm | 36.57 | 39.14 | 2.57 | 2.58 | -0.64 | 33.0 | -0.07 | 3.19 | -0.47 | 35.3 | 0.68 |
| **NT21_10** | F | Term | 37.57 | 40.57 | 3.00 | 3.13 | 0.44 | 31.5 | -1.44 | 3.40 | -0.22 | 33.6 | -0.68 |
| **NT21_11** | F | Early Preterm | 32.29 | 36.14 | 3.85 | 2.50 | 2.16 | 32.0 | 1.34 | 2.81 | 0.44 | 33.9 | 1.08 |
| **NT21_12** | F | Term | 38.14 | 41.43 | 3.29 | 2.69 | -0.89 | 31.2 | -1.96 | 2.62 | -2.40 | 30.8 | -3.88 |
| **NT21_13** | M | Term | 41.71 | 43.00 | 1.29 | 3.38 | -0.86 | 33.2 | -2.26 | 3.54 | -0.98 | 32.7 | -3.31 |
| **NT21_16** | F | Term | 37.71 | 43.57 | 5.86 | 3.17 | 0.46 | 32.0 | -1.08 | 3.83 | -0.28 | 34.6 | -1.36 |
| **NT21_17** | M | Term | 38.43 | 39.57 | 1.14 | 2.90 | -0.81 | 33.0 | -0.91 | 2.90 | -1.29 | 33.5 | -0.98 |
| **NT21_18** | M | Late Preterm | 36.71 | 44.57 | 7.86 | 3.06 | 0.38 | 33.0 | -0.13 | 3.27 | -2.36 | 33.1 | -3.63 |
| **NT21_19** | M | Term | 37.57 | 44.57 | 7.00 | 2.95 | -0.31 | 31.0 | -2.07 | 4.56 | 0.04 | 36.2 | -1.02 |
| **NT21_21** | F | Early Preterm | 32.00 | 32.43 | 0.43 | 1.86 | 0.46 | 29.5 | -0.22 | 1.86 | 0.18 | 30.0 | -0.08 |
| **NT21_22** | M | Term | 37.00 | 37.57 | 0.57 | 2.50 | -1.04 | 31.5 | -1.41 | 2.40 | -1.55 | 32.3 | -1.06 |
| **NT21_23** | M | Late Preterm | 35.29 | 38.86 | 3.57 | 2.00 | -1.34 |  |  | 2.20 | -2.63 | 31.5 | -2.36 |
| **NT21_24** | F | Term | 37.00 | 37.86 | 0.86 | 3.01 | 0.45 | 32.0 | -0.78 | 3.01 | 0.02 | 32.7 | -0.56 |
| **NT21_25** | M | Term | 37.14 | 38.00 | 0.86 | 2.33 | -1.50 | 32.0 | -1.09 | 2.26 | -2.08 | 32.2 | -1.36 |
| **NT21_26** | M | Early Preterm | 31.71 | 34.14 | 2.43 | 1.66 | -0.28 | 29.0 | -0.55 | 1.80 | -1.24 | 29.9 | -1.17 |
| **NT21_29** | M | Term | 38.43 | 39.14 | 0.71 | 3.36 | 0.21 | 34.0 | -0.10 | 3.38 | -0.05 | 34.2 | -0.25 |
| **NT21_30** | F | Term | 37.57 | 38.86 | 1.29 | 2.89 | -0.12 | 30.8 | -2.04 | 2.98 | -0.54 | 31.8 | -1.71 |
| **NT21_31** | F | Term | 39.86 | 40.86 | 1.00 | 3.55 | 0.38 | 34.5 | 0.40 | 3.75 | 0.50 | 33.6 | -0.84 |
| **NT21_32** | F | Term | 38.00 | 43.71 | 5.71 | 2.52 | -1.24 | 32.4 | -0.85 | 2.61 | -2.99 | 33.0 | -2.76 |
| **NT21_33** | M | Late Preterm | 36.29 | 42.29 | 6.00 | 2.70 | -0.21 |  |  | 4.08 | 0.37 | 37.5 | 1.28 |
| **NT21_34** | F | Late Preterm | 36.14 | 40.71 | 4.57 | 2.44 | -0.44 | 31.0 | -1.21 | 3.11 | -0.98 | 34.0 | -0.37 |
| **NT21_35** | M | Term | 38.57 | 45.57 | 7.00 | 3.11 | -0.40 | 33.2 | -0.81 | 3.60 | -2.15 | 36.5 | -1.17 |
| **NT21_36** | M | Term | 37.43 | 39.71 | 2.29 | 2.77 | -0.63 | 32.0 | -1.23 | 2.93 | -1.28 | 33.0 | -1.50 |

**Table S2:** Congenital heart defects (CHD) in neonates with DS.

**Columns:** in blue = data from clinical records on day of scan, prior to surgery; in yellow = details of later surgery; in green = categorical labels applied for research purposes.

| **ID** | **CHD type(s)** | **CHD details** | **Evidence of low SPO2** | **Age at surgery (months) (EPR)** | **Surgery details**  **(EPR)** | **CHD** | **Cyanotic/**  **Acyanotic** | **Severity Classification** |
| --- | --- | --- | --- | --- | --- | --- | --- | --- |
| **NT21_6** |  |  |  |  |  |  |  |  |
| **NT21_8** | AVSD, RAA | Complete AVSD, Bi-directional shunt, Right-sided aortic arch. | Yes (below 90%) | 2.87 | AVSD repair | Yes | Cyanotic | Serious |
| **NT21_9** |  |  |  |  |  |  |  |  |
| **NT21_10** |  |  |  |  |  |  |  |  |
| **NT21_11** | Small VSD, PVS, RAA | Small VSD, Pulmonary valve stenosis, Right-sided aortic arch, PDA. | No evidence | N/A | No cardiac surgery. | Yes | Acyanotic | Significant |
| **NT21_12** | AVSD, ToF | AVSD (L to R shunt), ToF (tetralogy canal defect), PDA. | Yes (below 90%) | 4.30 | Elective AVSD closure and PDA ligation | Yes | Cyanotic | Serious |
| **NT21_13** | Small ASD, Persistent PFO | Small ASD, Persistent PFO. | No evidence | N/A | No cardiac surgery. | Yes | Acyanotic | Significant |
| **NT21_16** | AVSD, Small LV, CoA/HAA | Complete unbalanced AVSD, Small LV, Coarctation of the aorta / Hypoplastic aortic arch, PDA. | No evidence (high 90s %) | 3.33 | CoA repair and PA banding | Yes | Acyanotic | Serious |
| **NT21_17** | AVSD, Small LV | Complete AVSD, Small LV, Mild right AV valve regurgitation. | No evidence (high 90s %) | 3.27 | AVSD repair | Yes | Acyanotic | Serious |
| **NT21_18** |  |  |  |  |  |  |  |  |
| **NT21_19** |  |  |  |  |  |  |  |  |
| **NT21_21** |  |  |  |  |  |  |  |  |
| **NT21_22** | AVSD, CoA | Complete AVSD, Coarctation of the aorta. | Yes (below 90%) | 0.37 | CoA repair and PA banding | Yes | Cyanotic | Critical |
| **NT21_23** | Small ASD | Small ASD. | No evidence | N/A | No cardiac surgery. | Yes | Acyanotic | Non-significant |
| **NT21_24** | AVSD | Complete AVSD. | Yes (below 90%) | 5.80 | AVSD repair | Yes | Cyanotic | Serious |
| **NT21_25** |  |  |  |  |  |  |  |  |
| **NT21_26** |  |  |  |  |  |  |  |  |
| **NT21_29** |  |  |  |  |  |  |  |  |
| **NT21_30** |  |  |  |  |  |  |  |  |
| **NT21_31** | Small VSD, Small ASD | Small VSD, Small secundum ASD. | No evidence | 4.07 | Closure of VSD and ASD | Yes | Acyanotic | Serious |
| **NT21_32** | AVSD, CoA | Complete AVSD, Coarctation of the aorta, Bi-directional shunt. | Yes (below 90%) | 0.13 | CoA repair and PA banding | Yes | Cyanotic | Critical |
| **NT21_33** |  |  |  |  |  |  |  |  |
| **NT21_34** | Small ASD | Small ASD, Large PDA (L to R shunt). | No evidence | 5.80 | Attempted PDA occlusion by cardiac catheterisation | Yes | Acyanotic | Serious |
| **NT21_35** |  |  |  |  |  |  |  |  |
| **NT21_36** | AVSD, HLH, HAA | Complete unbalanced AVSD [large VSD (inlet), L-to-R shunt, Primum ASD, Dominant right ventricle and right AV valve, PFO), Hypoplastic left heart, Hypoplastic aortic arch. | Yes (below 90%) | 1.03 | PA banding | Yes | Cyanotic | Critical |

**Abbreviations:** *ASD = Atrial Septal Defect; AVSD = Atrioventricular Septal Defect; CoA = Coarctation of the Aorta; HAA = Hypoplastic Aortic Arch; HLH = Hypoplastic Left Heart Syndrome; LV = Left Ventricle; PDA = Patent Ductus Arteriosus; PFO = Patent Foramen Ovale; PVS = Pulmonary valve stenosis; RAA: Right-sided Aortic Arch; SPO2 = oxygen saturation; ToF = Tetralogy of Fallot; VSD = Ventricular Septal Defect.*

**Table S3:** Additional clinical details for neonates with DS.

| **ID** | **GI**  **issue** | **GI detail** | **GI surgery** | **Infection** | **Antibiotics** | **Haematological**  **issue** | **Metabolic**  **issue** | **Jaundice /**  **Phototherapy** |
| --- | --- | --- | --- | --- | --- | --- | --- | --- |
| **NT21_6** |  |  |  |  |  |  | Hypoglycaemia | Yes |
| **NT21_8** |  |  |  | sepsis suspected | Yes |  |  | Yes |
| **NT21_9** | Yes | Duodenal Atresia. | Yes, prior |  |  | Polycythaemia |  | Yes |
| **NT21_10** | Yes | Duodenal Atresia. | Yes, prior |  |  |  |  |  |
| **NT21_11** | Yes | Duodenal stricture. Imperforate anus. | Yes, prior | sepsis suspected | Yes |  | Hypoglycaemia | Yes |
| **NT21_12** | Yes | Duodenal Atresia. | Yes, prior |  |  |  |  |  |
| **NT21_13** | Yes | Hirschsprung Disease. | Yes, after | sepsis confirmed | Yes | Thrombocytopenia (platelet transfusion) |  |  |
| **NT21_16** |  |  |  | sepsis confirmed | Yes |  | Hypoglycaemia |  |
| **NT21_17** |  |  |  |  |  |  |  |  |
| **NT21_18** |  |  |  | sepsis suspected | Yes | Thrombocytopenia |  |  |
| **NT21_19** |  |  |  |  |  |  |  |  |
| **NT21_21** |  |  |  | sepsis confirmed | Yes |  |  |  |
| **NT21_22** |  |  |  |  |  |  |  |  |
| **NT21_23** | Yes | Hirschsprung Disease. | Yes, after | sepsis confirmed | Yes |  |  | Yes |
| **NT21_24** |  |  |  |  |  |  |  |  |
| **NT21_25** |  |  |  |  |  |  |  |  |
| **NT21_26** | Yes | Duodenal Atresia. | Yes, prior |  |  |  |  | Yes |
| **NT21_29** |  |  |  | sepsis suspected | Yes |  |  | Yes |
| **NT21_30** | Yes | Duodenal Atresia. | Yes, prior |  |  | Thrombocytopenia, Polycythaemia |  | Yes |
| **NT21_31** |  |  |  | sepsis suspected | Yes |  | Raised TSH and/or Hypothyroidism |  |
| **NT21_32** |  |  |  |  |  |  | Raised TSH and/or Hypothyroidism, Hypoglycaemia |  |
| **NT21_33** |  |  |  |  |  |  |  |  |
| **NT21_34** |  |  |  | sepsis suspected | Yes |  | Hypoglycaemia | Yes |
| **NT21_35** |  |  |  | sepsis suspected | Yes | Polycythaemia |  | Yes |
| **NT21_36** |  |  |  |  |  |  | Hyponatremia |  |

**Abbreviations:**

*GI = Gastrointestinal.*

*GI surgery: ‘Yes, prior’ = prior to neonatal scan. ‘Yes, after’ = after neonatal scan.*

*TSH = Thyroid stimulating hormone*

**Table S4:** Volumetric brain development in control group from 32 to < 46 weeks PMA.

Table detailing mean absolute volume (in cm^3^) and mean relative volume (in %) for **A)** whole brain, **B)** main tissue classes, **C)** cortical GM segments, **D)** WM segments, **E)** deep GM and other segments for the control cohort at 32- and 45-weeks PMA. Male and female control neonates (n = 493) have been consolidated. Total % change, as well as an estimated % change per week (pw) are provided. This table is complementary to the graphs in Figure S2.

|  | Mean absolute volume (cm3) 32 weeks PMA | Mean absolute volume (cm3) 45 weeks PMA | Total % Change | Est. % Change per week |  | Mean relative volume (%) 32 weeks PMA | Mean relative volume (%) 45 weeks PMA | Total % Change | Est. % Change per week |
| --- | --- | --- | --- | --- | --- | --- | --- | --- | --- |
| **A) Whole Brain** |  |  |  |  |  |  |  |  |  |
| ICV | 234.3 | 539.9 | 130% | 10.0% |  | - | - | - | - |
| TBV | 187.7 | 444.6 | 137% | 10.5% |  | - | - | - | - |
| TTV | 183.1 | 437.8 | 139% | 10.7% |  | - | - | - | - |
|  |  |  |  |  |  |  |  |  |  |
| **B) Main Tissue Classes** |  |  |  |  |  |  |  |  |  |
| Cortical Grey Matter | 54.8 | 197.9 | 261% | 20.1% |  | 28.8% | 45.1% | 56.8% | 4.4% |
| Cerebellum | 9.6 | 33.7 | 252% | 19.4% |  | 5.2% | 7.7% | 49.0% | 3.8% |
| eCSF | 43.4 | 92.5 | 113% | 8.7% |  | 20.0% | 16.5% | -17.7% | -1.4% |
| Deep Grey Matter | 15.2 | 31.6 | 108% | 8.3% |  | 8.5% | 7.3% | -14.7% | -1.1% |
| Brainstem | 3.9 | 7.4 | 93% | 7.1% |  | 2.2% | 1.7% | -20.6% | -1.6% |
| White Matter | 98.8 | 163.7 | 66% | 5.0% |  | 54.6% | 37.4% | -31.4% | -2.4% |
| Lateral Ventricles | 3.6 | 5.8 | 60% | 4.6% |  | 2.4% | 1.3% | -47.4% | -3.6% |
|  |  |  |  |  |  |  |  |  |  |
| **C) Cortical GM** |  |  |  |  |  |  |  |  |  |
| Parietal Lobe GM | 12.4 | 47.9 | 285% | 22.0% |  | 6.5% | 10.9% | 66.4% | 5.1% |
| Occipital Lobe GM | 8.4 | 30.9 | 266% | 20.5% |  | 4.5% | 7.0% | 56.9% | 4.4% |
| Frontal Lobe GM | 18.2 | 66.2 | 264% | 20.3% |  | 9.6% | 15.0% | 56.0% | 4.3% |
| Temporal Lobe GM | 11.4 | 41.1 | 262% | 20.1% |  | 6.0% | 9.3% | 54.8% | 4.2% |
| Insula GM | 1.6 | 4.4 | 174% | 13.4% |  | 0.9% | 1.0% | 16.2% | 1.2% |
| Cingulate GM | 2.8 | 7.4 | 161% | 12.4% |  | 1.6% | 1.7% | 9.0% | 0.7% |
|  |  |  |  |  |  |  |  |  |  |
| **D) WM** |  |  |  |  |  |  |  |  |  |
| Cingulate WM | 3.2 | 5.8 | 82% | 6.3% |  | 1.7% | 1.3% | -23.5% | -1.8% |
| Temporal Lobe WM | 18.2 | 32.0 | 76% | 5.8% |  | 10.1% | 7.4% | -27.0% | -2.1% |
| Frontal Lobe WM | 38.3 | 62.6 | 63% | 4.9% |  | 21.3% | 14.4% | -32.2% | -2.5% |
| Parietal Lobe WM | 23.1 | 37.5 | 63% | 4.8% |  | 12.9% | 8.6% | -33.4% | -2.6% |
| Insula WM | 3.7 | 6.0 | 61% | 4.7% |  | 2.0% | 1.4% | -33.5% | -2.6% |
| Occipital Lobe WM | 10.6 | 16.1 | 52% | 4.0% |  | 5.9% | 3.6% | -39.1% | -3.0% |
|  |  |  |  |  |  |  |  |  |  |
| **E) Deep GM and Other** |  |  |  |  |  |  |  |  |  |
| Lentiform Nucleus | 3.1 | 7.7 | 147% | 11.3% |  | 1.7% | 1.8% | 1.6% | 0.1% |
| Thalamus | 5.1 | 11.1 | 117% | 9.0% |  | 2.9% | 2.5% | -11.0% | -0.8% |
| Amygdala | 0.5 | 1.1 | 111% | 8.5% |  | 0.3% | 0.3% | -13.8% | -1.1% |
| Caudate Nucleus | 2.1 | 4.3 | 103% | 7.9% |  | 1.2% | 1.0% | -17.6% | -1.4% |
| Hippocampus | 1.0 | 1.8 | 76% | 5.9% |  | 0.6% | 0.4% | -29.8% | -2.3% |

**Supplementary text:**

The period from 32 up to < 46 weeks PMA at scan was a phase of rapid whole brain volume expansion (*section A of table*). The average intracranial volume (ICV) in control neonates grew more than 2-fold (+130%, an estimated +10% per week, pw) from 234.3 cm^3^ at 32 weeks to 539.9 cm^3^ by 45 weeks (males and females consolidated). Similarly, the average total tissue volume (TTV), which represents ICV minus CSF-filled structures (i.e., eCSF and lateral ventricles) grew +139% (estimated +10.7% pw) from 183.1 cm^3^ to 437.8 cm^3^.

Looking at the main tissue classes of the brain (*section B*), we found that the average total cortical GM grew approximately 3.5-fold (+261%, +20.1% pw) from 54.8 cm^3^ to 197.9 cm^3^, representing the fastest growing tissue class in the brain during this period. This was composed of, in order from fastest to slowest growing GM segments, the parietal, occipital, frontal, temporal, insular and cingulate GM, which grew between +285% and +161% during this period (*section C*). Another fast-growing tissue type was the cerebellum, which grew 3.5-fold during this period (+252%, +19.4% pw) from 9.6 to 33.7 cm^3^ (*section B*). Total deep GM showed a relatively moderate growth of +108% (+8.3% pw), comprised of the lentiform nuclei (+ 147%), the thalami (+117%) and the caudate nuclei (+103%) (*section E*). The brainstem grew relatively moderately compared to other tissue at +93% (+ 7.1% pw, from 3.9 to 7.4 cm^3^) (*section B*). Total WM only grew +66% (+5.0% pw, from 98.8 to 163.7 cm^3^), representing the slowest-growing major tissue class during this period. This was composed of, in order from fastest to slowest growing WM segments, the cingulate, temporal, frontal, parietal, insular and occipital WM, which grew between +82% (+6.3% pw) and +52% (+4.0% pw) during this period (*section D*). Finally, the eCSF grew moderately (+113%), whilst the lateral ventricles grew the least from 3.6 to 5.8 cm^3^ (+60%) over this period (*section B*).

The relative volume of a tissue type indicated its share of the whole brain (as detailed in Table 1 in main text). Total cortical GM grew its relative share of TTV the fastest from 28.8% at 32 weeks to 45.1% at 45 weeks (+ 56.8% change), followed by the cerebellum, which grew from 5.2% to 7.7% of TTV (+ 49.0% change). In contrast, the share of total WM declined from 54.6% to 37.4% of TTV (-31.4% change) during this period. Relative volumes for the brainstem (-20.6%), deep GM structures (-14.7%), eCSF (-17.7%) and lateral ventricles (-47.4%) all declined during this period as they were outcompeted by faster-growing tissue types (*section B*)*.*

**Table S5:** Table of results for the extra sum-of-squares F tests comparing DS and control simple linear regressions (absolute volume z-scores against PMA at scan).

Table of results for the extra sum-of-squares F tests comparing the parameters (i.e., slope and intercept) of DS and control simple linear regressions for absolute volume z-scores against PMA at scan. All corresponding simple linear regression plots can be found in Figure S3. ‘F’ = F ratio, ‘DFn’ = degree of freedom for the numerator of the F ratio, ‘DFd’ = degree of freedom for the denominator of the F ratio, ‘Sig.’ = significance level. The uncorrected P-value, as well as the FDR-corrected P-value are shown. A cell highlighted in green indicates a P-value < 0.05.

|  | **Extra Sum-of-Squares F Test** | | | | | | | | | | | | | | |
| --- | --- | --- | --- | --- | --- | --- | --- | --- | --- | --- | --- | --- | --- | --- | --- |
|  | **DS All (n = 25) vs Control (n = 493)** | | | | | | | | | | | | | | |
|  | ***Are the slopes equal?*** | | | | | | |  | ***Are the elevations or intercepts equal?*** | | | | | | |
|  | **F ratio** | **DFn** | **DFd** | **P value (uncorrected)** | **Sig** | **pFDR** | **Sig** |  | **F ratio** | **DFn** | **DFd** | **P value (uncorrected)** | **Sig** | **pFDR** | **Sig** |
| **Whole Brain Volumes** |  |  |  |  |  |  |  |  |  |  |  |  |  |  |  |
| ICV | 11.45 | 1 | 514 | 0.0008 | *** | 0.0021 | ** |  | - | - | - | - | - | - | - |
| TTV | 5.42 | 1 | 514 | 0.0203 | * | 0.0338 | * |  | - | - | - | - | - | - | - |
|  |  |  |  |  |  |  |  |  |  |  |  |  |  |  |  |
| **Main Tissue Volumes** |  |  |  |  |  |  |  |  |  |  |  |  |  |  |  |
| Cortical GM | 6.32 | 1 | 514 | 0.0122 | * | 0.0222 | * |  | - | - | - | - | - | - | - |
| Deep GM | 1.63 | 1 | 514 | 0.2026 | ns | 0.2701 | ns |  | 35.72 | 1 | 515 | <0.0001 | **** | 0.0003 | *** |
| WM | 2.52 | 1 | 514 | 0.1130 | ns | 0.1614 | ns |  | 78.07 | 1 | 515 | <0.0001 | **** | 0.0003 | *** |
| Cerebellum | 13.15 | 1 | 514 | 0.0003 | *** | 0.0009 | *** |  | - | - | - | - | - | - | - |
| Brainstem | 6.36 | 1 | 514 | 0.0120 | * | 0.0222 | * |  | - | - | - | - | - | - | - |
| eCSF | 19.05 | 1 | 514 | <0.0001 | **** | 0.0003 | *** |  | - | - | - | - | - | - | - |
| Lateral Ventricles | 10.01 | 1 | 514 | 0.0016 | ** | 0.0038 | ** |  | - | - | - | - | - | - | - |
| Lateral Ventricles (ex-outlier) | 1.61 | 1 | 513 | 0.2054 | ns | 0.2710 | ns |  | 29.40 | 1 | 514 | <0.0001 | **** | 0.0003 | *** |
|  |  |  |  |  |  |  |  |  |  |  |  |  |  |  |  |
| **Cortical GM segments** |  |  |  |  |  |  |  |  |  |  |  |  |  |  |  |
| Temporal Lobe GM | 1.52 | 1 | 514 | 0.2189 | ns | 0.2736 | ns |  | 26.96 | 1 | 515 | <0.0001 | **** | 0.0003 | *** |
| Frontal Lobe GM | 10.10 | 1 | 514 | 0.0016 | ** | 0.0038 | ** |  | - | - | - | - | - | - | - |
| Parietal Lobe GM | 4.17 | 1 | 514 | 0.0417 | * | 0.0642 | ns |  | - | - | - | - | - | - | - |
| Occipital Lobe GM | 6.16 | 1 | 514 | 0.0134 | * | 0.0233 | * |  | - | - | - | - | - | - | - |
| Cingulate Lobe GM | 7.45 | 1 | 514 | 0.0066 | ** | 0.0132 | * |  | - | - | - | - | - | - | - |
| Insular Lobe GM | 0.34 | 1 | 514 | 0.5589 | ns | 0.5732 | ns |  | 25.05 | 1 | 515 | <0.0001 | **** | 0.0003 | *** |
|  |  |  |  |  |  |  |  |  |  |  |  |  |  |  |  |
| **WM segments** |  |  |  |  |  |  |  |  |  |  |  |  |  |  |  |
| Temporal Lobe WM | 0.80 | 1 | 514 | 0.3707 | ns | 0.4119 | ns |  | 52.16 | 1 | 515 | <0.0001 | **** | 0.0003 | *** |
| Frontal Lobe WM | 3.65 | 1 | 514 | 0.0567 | ns | 0.0840 | ns |  | 100.80 | 1 | 515 | <0.0001 | **** | 0.0003 | *** |
| Parietal Lobe WM | 0.89 | 1 | 514 | 0.3455 | ns | 0.3949 | ns |  | 16.71 | 1 | 515 | <0.0001 | **** | 0.0003 | *** |
| Occipital Lobe WM | 1.06 | 1 | 514 | 0.3035 | ns | 0.3679 | ns |  | 64.97 | 1 | 515 | <0.0001 | **** | 0.0003 | *** |
| Cingulate Lobe WM | 0.92 | 1 | 514 | 0.3386 | ns | 0.3949 | ns |  | 181.60 | 1 | 515 | <0.0001 | **** | 0.0003 | *** |
| Insular Lobe WM | 2.36 | 1 | 514 | 0.1247 | ns | 0.1720 | ns |  | 93.57 | 1 | 515 | <0.0001 | **** | 0.0003 | *** |
|  |  |  |  |  |  |  |  |  |  |  |  |  |  |  |  |
| **Deep GM & Other** |  |  |  |  |  |  |  |  |  |  |  |  |  |  |  |
| Hippocampus | 8.11 | 1 | 514 | 0.0046 | ** | 0.0097 | ** |  | - | - | - | - | - | - | - |
| Amygdala | 0.69 | 1 | 514 | 0.4071 | ns | 0.4401 | ns |  | 35.50 | 1 | 515 | <0.0001 | **** | 0.0003 | *** |
| Caudate Nucleus | 1.57 | 1 | 514 | 0.2114 | ns | 0.2728 | ns |  | 52.97 | 1 | 515 | <0.0001 | **** | 0.0003 | *** |
| Lentiform Nucleus | 0.03 | 1 | 514 | 0.8580 | ns | 0.8580 | ns |  | 0.54 | 1 | 515 | 0.4642 | ns | 0.4886 | ns |
| Thalamus | 4.80 | 1 | 514 | 0.0290 | * | 0.0464 | * |  | - | - | - | - | - | - | - |

**Table S6:** Table of results for Spearman’s rank correlation tests (for absolute volume z-scores against PMA at scan).

Table of results for Spearman’s rank correlation tests assessing the correlation of absolute z-scores and PMA at scan (in weeks). All corresponding plots can be found in Figure S3. The uncorrected P-value, as well as the FDR-corrected P-value are shown. A cell highlighted in green indicates a P-value < 0.05. The R^2^ value is provided to assess linear model goodness of fit.

|  | ***Correlation of absolute z-scores and PMA at scan*** | | | | | |
| --- | --- | --- | --- | --- | --- | --- |
|  | **DS All (n = 25)** | | | | | |
|  | **Spearman's Rho** | **uncorrected P value** | **Sig.** | **pFDR** | **Sig.** | **R^2^** |
| **Whole Brain Volumes** |  |  |  |  |  |  |
| ICV | -0.52 | 0.0077 | ** | 0.0824 | ns | 0.24 |
| TTV | -0.36 | 0.0755 | ns | 0.1853 | ns | 0.15 |
|  |  |  |  |  |  |  |
| **Main Tissue Volumes** |  |  |  |  |  |  |
| Cortical GM | -0.29 | 0.1536 | ns | 0.2680 | ns | 0.13 |
| Deep GM | -0.12 | 0.5838 | ns | 0.6369 | ns | 0.04 |
| WM | -0.31 | 0.1263 | ns | 0.2436 | ns | 0.11 |
| Cerebellum | -0.54 | 0.0058 | ** | 0.0824 | ns | 0.29 |
| Brainstem | -0.34 | 0.0945 | ns | 0.1963 | ns | 0.17 |
| eCSF | -0.44 | 0.0279 | * | 0.1256 | ns | 0.23 |
| Lateral Ventricles | -0.39 | 0.0540 | ns | 0.1620 | ns | 0.18 |
|  |  |  |  |  |  |  |
| **Cortical GM segments** |  |  |  |  |  |  |
| Temporal Lobe GM | -0.11 | 0.6133 | ns | 0.6369 | ns | 0.03 |
| Frontal Lobe GM | -0.38 | 0.0642 | ns | 0.1733 | ns | 0.20 |
| Parietal Lobe GM | -0.19 | 0.3636 | ns | 0.4675 | ns | 0.08 |
| Occipital Lobe GM | -0.47 | 0.0177 | * | 0.0956 | ns | 0.22 |
| Cingulate Lobe GM | -0.41 | 0.0440 | * | 0.1485 | ns | 0.19 |
| Insular Lobe GM | 0.11 | 0.5914 | ns | 0.6369 | ns | 0.01 |
|  |  |  |  |  |  |  |
| **WM segments** |  |  |  |  |  |  |
| Temporal Lobe WM | -0.20 | 0.3299 | ns | 0.4454 | ns | 0.03 |
| Frontal Lobe WM | -0.42 | 0.0386 | * | 0.1485 | ns | 0.15 |
| Parietal Lobe WM | -0.26 | 0.2084 | ns | 0.3210 | ns | 0.04 |
| Occipital Lobe WM | -0.35 | 0.0883 | ns | 0.1963 | ns | 0.06 |
| Cingulate Lobe WM | -0.21 | 0.3178 | ns | 0.4454 | ns | 0.05 |
| Insular Lobe WM | -0.17 | 0.4162 | ns | 0.5108 | ns | 0.08 |
|  |  |  |  |  |  |  |
| **Deep GM & Other** |  |  |  |  |  |  |
| Hippocampus | -0.49 | 0.0122 | * | 0.0824 | ns | 0.24 |
| Amygdala | -0.11 | 0.5991 | ns | 0.6369 | ns | 0.02 |
| Caudate Nucleus | -0.26 | 0.2140 | ns | 0.3210 | ns | 0.05 |
| Lentiform Nucleus | -0.02 | 0.9229 | ns | 0.9229 | ns | 0.00 |
| Thalamus | -0.29 | 0.1588 | ns | 0.2680 | ns | 0.11 |

**Table S7:** Group comparison of whole brain volume-adjusted medians (derived from covariation analysis) between DS and control neonates.

Table of unadjusted and whole brain volume (WBV)-adjusted median absolute volume z-scores for neonates with DS (n = 25) and control neonates (n = 493). Group medians were adjusted using median regression (as per McGreevy et al. 2009). A Mann-Whitney U test was conducted to compare DS vs control WBV-adjusted medians, which was corrected for multiple comparisons (pFDR). A colour scale has been applied to DS WBV-adjusted medians, whereby red indicates a negative deviation (adjusted median z < 0), white indicates no significant deviation (z ~ 0), and blue indicates a positive deviation from the normative mean (adjusted median z > 0). A cell highlighted in green indicates a pFDR value < 0.05.

|  | **DS** | | | | **Control** | | | | **Mann-Whitney U test with FDR corr.** | |
| --- | --- | --- | --- | --- | --- | --- | --- | --- | --- | --- |
| **Tissue segment** | **Unadjusted median** | **Adjusted median** | **95% CI Lower** | **95% CI Upper** | **Unadjusted median** | **Adjusted median** | **95% CI Lower** | **95% CI Upper** | **W** | **pFDR** |
| **Main Tissue Volumes** |  |  |  |  |  |  |  |  |  |  |
| Cortical GM | -1.22 | 0.615 | 0.302 | 0.928 | -0.05 | -0.015 | -0.042 | 0.012 | 1324 | **<0.0001** |
| Deep GM | -1.04 | 0.289 | -0.205 | 0.783 | 0.03 | -0.014 | -0.072 | 0.045 | 3642 | **0.0004** |
| WM | -1.91 | -0.692 | -1.109 | -0.274 | -0.03 | 0.003 | -0.025 | 0.031 | 10746 | **<0.0001** |
| Cerebellum | -2.49 | -0.699 | -1.379 | -0.018 | -0.04 | -0.007 | -0.081 | 0.066 | 9964 | **<0.0001** |
| Brainstem | -1.33 | 0.049 | -0.411 | 0.509 | -0.02 | -0.021 | -0.081 | 0.040 | 5054 | 0.09 |
| eCSF | 0.27 | 1.794 | 1.047 | 2.541 | -0.02 | -0.004 | -0.077 | 0.069 | 1159 | **<0.0001** |
| Lateral Ventricles | 1.05 | 2.438 | 1.831 | 3.044 | -0.18 | -0.156 | -0.249 | -0.063 | 1142 | **<0.0001** |
|  |  |  |  |  |  |  |  |  |  |  |
| **Cortical GM segments** |  |  |  |  |  |  |  |  |  |  |
| Temporal Lobe GM | -0.72 | 0.939 | 0.384 | 1.494 | -0.06 | 0.000 | -0.045 | 0.046 | 1781 | **<0.0001** |
| Frontal Lobe GM | -1.19 | 0.306 | -0.032 | 0.645 | -0.13 | -0.011 | -0.060 | 0.038 | 3821 | **0.0006** |
| Parietal Lobe GM | -0.43 | 1.038 | 0.283 | 1.793 | -0.01 | -0.012 | -0.070 | 0.046 | 1182 | **<0.0001** |
| Occipital Lobe GM | -1.24 | -0.268 | -0.806 | 0.270 | -0.03 | -0.014 | -0.070 | 0.041 | 7599 | 0.13 |
| Cingulate GM | -1.13 | -0.031 | -0.655 | 0.592 | -0.07 | -0.035 | -0.105 | 0.035 | 5212 | 0.13 |
| Insular GM | -0.66 | 0.460 | -0.339 | 1.258 | -0.07 | 0.016 | -0.052 | 0.085 | 3952 | **0.0015** |
|  |  |  |  |  |  |  |  |  |  |  |
| **WM segments** |  |  |  |  |  |  |  |  |  |  |
| Temporal Lobe WM | -1.55 | -0.240 | -0.679 | 0.198 | -0.03 | 0.013 | -0.036 | 0.063 | 7223 | 0.30 |
| Frontal Lobe WM | -2.00 | -1.010 | -1.636 | -0.383 | -0.04 | 0.008 | -0.051 | 0.067 | 11415 | **<0.0001** |
| Parietal Lobe WM | -0.89 | 0.280 | -0.397 | 0.957 | -0.05 | 0.004 | -0.045 | 0.052 | 4821 | **0.0468** |
| Occipital Lobe WM | -1.76 | -0.646 | -1.308 | 0.016 | -0.03 | -0.004 | -0.067 | 0.060 | 10522 | **<0.0001** |
| Cingulate WM | -2.72 | -1.895 | -2.383 | -1.408 | -0.05 | -0.060 | -0.129 | 0.010 | 12659 | **<0.0001** |
| Insular WM | -1.93 | -0.664 | -1.153 | -0.174 | -0.02 | 0.017 | -0.057 | 0.092 | 9936 | **<0.0001** |
|  |  |  |  |  |  |  |  |  |  |  |
| **Deep GM & Other** |  |  |  |  |  |  |  |  |  |  |
| Hippocampus | -1.52 | -0.186 | -0.952 | 0.580 | -0.11 | -0.059 | -0.149 | 0.031 | 7327 | 0.25 |
| Amygdala | -1.14 | 0.095 | -0.683 | 0.873 | -0.02 | -0.011 | -0.079 | 0.058 | 6763 | 0.64 |
| Caudate Nucleus | -1.54 | -0.067 | -0.450 | 0.316 | -0.09 | -0.060 | -0.121 | 0.001 | 7033 | 0.42 |
| Lentiform Nucleus | -0.03 | 1.033 | 0.585 | 1.480 | -0.05 | 0.010 | -0.048 | 0.068 | 2170 | **<0.0001** |
| Thalamus | -0.82 | 0.355 | -0.376 | 1.087 | -0.02 | 0.042 | -0.014 | 0.097 | 3707 | **0.0005** |

**Table S8:** Group comparison of absolute volume z-scores between CHD+ and CHD- neonates.

Table of median absolute volume z-scores for the DS neonates with CHD (CHD+, n = 13) and without CHD (CHD-, n = 12). The table is organised into the following sub-sections**: A)** whole brain volumes, **B)** total GM or WM volumes, **C)** regional volumes and **D)** specific tissue volumes (including CSF-filled volumes). A non-parametric Kruskal-Wallis test with FDR multiple comparison correction (pFDR) was performed for each tissue label. Uncorrected P-values are also displayed for information. Cliff’s delta (*d*) test was used to assess the effect size. A colour scale has been applied, whereby red indicates a negative deviation from the normative mean (z < 0, i.e., a smaller volume than norm), white indicates no significant deviation (z = 0), and blue indicates a positive deviation (z > 0, i.e., a larger volume than norm). A cell highlighted in green indicates a P-value < 0.05 or a large effect size.

| **A) Whole Brain Volumes** | **CHD+ median (n = 13)** | **CHD- median (n = 12)** | **Kruskal Wallis (uncorrected P-value)** | **Kruskal Wallis (pFDR)** | **Sig.** | **Cliff's delta** | **Effect size** |
| --- | --- | --- | --- | --- | --- | --- | --- |
| ICV | -1.83 | -0.62 | 0.1078 | 0.2668 | ns | -0.35 | medium |
| TBV | -2.08 | -1.02 | 0.0684 | 0.2668 | ns | -0.46 | medium |
| TTV | -2.15 | -1.16 | 0.0785 | 0.2668 | ns | -0.44 | medium |
| **B) Total GM or WM** |  |  |  |  |  |  |  |
| Total White Matter | -2.10 | -1.52 | 0.2025 | 0.3159 | ns | -0.40 | medium |
| Cortical Grey Matter | -1.71 | -0.51 | 0.0367 | 0.2455 | ns | -0.53 | large |
| Deep Grey Matter | -1.26 | -0.83 | 0.1058 | 0.2668 | ns | -0.46 | medium |
| **C) Regional Volumes** |  |  |  |  |  |  |  |
| Posterior Fossa | -2.74 | -2.08 | 0.4365 | 0.5320 | ns | -0.29 | small |
| Total Cingulate | -2.62 | -1.49 | 0.1367 | 0.2834 | ns | -0.42 | medium |
| Total Frontal Lobe | -2.02 | -1.38 | 0.2293 | 0.3227 | ns | -0.31 | small |
| Total Insula | -2.41 | -1.13 | 0.0631 | 0.2668 | ns | -0.47 | large |
| Total Occipital Lobe | -1.68 | -1.06 | 0.1855 | 0.3014 | ns | -0.45 | medium |
| Total Temporal Lobe | -2.10 | -0.60 | 0.0128 | 0.2455 | ns | -0.58 | large |
| Total Parietal Lobe | -1.13 | -0.44 | 0.1300 | 0.2834 | ns | -0.42 | medium |
| Basal Ganglia | -0.73 | -0.48 | 0.2317 | 0.3227 | ns | -0.29 | small |
| **D) Specific Tissue Volumes** |  |  |  |  |  |  |  |
| Cingulate WM | -3.04 | -2.28 | 0.5020 | 0.5594 | ns | -0.33 | medium |
| Cerebellum | -2.98 | -2.18 | 0.4713 | 0.5406 | ns | -0.24 | small |
| Insula WM | -2.65 | -1.52 | 0.1407 | 0.2834 | ns | -0.45 | medium |
| Frontal Lobe WM | -2.36 | -1.79 | 0.4680 | 0.5406 | ns | -0.28 | small |
| Temporal Lobe WM | -2.09 | -0.94 | 0.0504 | 0.2455 | ns | -0.46 | medium |
| Hippocampus | -2.08 | -0.75 | 0.1095 | 0.2668 | ns | -0.38 | medium |
| Caudate Nucleus | -1.98 | -0.82 | 0.0356 | 0.2455 | ns | -0.60 | large |
| Occipital Lobe WM | -1.89 | -1.45 | 0.2267 | 0.3227 | ns | -0.40 | medium |
| Temporal Lobe GM | -1.76 | 0.15 | 0.0064 | 0.2455 | ns | -0.59 | large |
| Cingulate GM | -1.76 | -0.85 | 0.0829 | 0.2668 | ns | -0.41 | medium |
| Occipital Lobe GM | -1.70 | -0.97 | 0.1621 | 0.2927 | ns | -0.36 | medium |
| Brainstem | -1.67 | -0.94 | 0.1821 | 0.3014 | ns | -0.36 | medium |
| Frontal Lobe GM | -1.66 | -0.77 | 0.1453 | 0.2834 | ns | -0.40 | medium |
| Amygdala | -1.42 | -1.11 | 0.3203 | 0.4307 | ns | -0.24 | small |
| Parietal Lobe GM | -1.22 | -0.13 | 0.0476 | 0.2455 | ns | -0.49 | large |
| Parietal Lobe WM | -1.19 | -0.77 | 0.4020 | 0.5057 | ns | -0.26 | small |
| Thalamus | -1.01 | -0.77 | 0.1651 | 0.2927 | ns | -0.28 | small |
| Insula GM | -0.92 | -0.39 | 0.0925 | 0.2668 | ns | -0.46 | medium |
| Lentiform Nucleus | -0.07 | 0.01 | 0.6915 | 0.7097 | ns | -0.09 | negligible |
| eCSF | 0.27 | 0.65 | 0.7726 | 0.7726 | ns | -0.08 | negligible |
| Lateral Ventricles | 1.00 | 1.80 | 0.6902 | 0.7097 | ns | -0.33 | medium |

Overall, CHD+ and CHD- neonates did not show any statistically significant groupwise differences after FDR multiple comparison correction. This was most likely due to low statistical power, as CHD subgroup sizes were small, and due to the large number of multiple comparisons. However, certain underlying trends were observed from the uncorrected P-values. In particular, the caudate nuclei (uncorrected P-value = 0.0356, pFDR = 0.25, d = -0.60, large effect) were smaller in CHD+ compared to CHD- neonates prior to multiple comparison correction. Furthermore, two GM segments, the temporal GM (uncorrected P-value = 0.0064, pFDR = 0.246, d = -0.59, large effect) and the parietal GM (uncorrected P-value = 0.0476, pFDR = 0.246, d = -0.49, large effect) were also smaller in CHD+ compared to CHD- neonates prior to multiple comparison correction. In future, larger subgroup sizes may help clarify which tissue segments significantly differ between subgroups.

**Table S9:** Table of results for the extra sum-of-squares F tests comparing simple linear regressions for CHD+ vs. CHD- neonates with DS (using absolute volume z-scores).

Table of results for the extra sum-of-squares F tests comparing the parameters (i.e., slope and intercept) of simple linear regressions using z-scores derived from absolute volumes for CHD+ vs CHD- neonates with DS. All corresponding simple linear regression plots can be found in Figure S4. ‘F’ = F ratio, ‘DFn’ = degree of freedom for the numerator of the F ratio, ‘DFd’ = degree of freedom for the denominator of the F ratio, ‘Sig.’ = significance level. The uncorrected P-value, as well as the FDR-corrected P-value are shown. A cell highlighted in green indicates a P-value < 0.05.

|  | **Extra Sum-of-Squares F Test** | | | | | | | | | | | | | | |
| --- | --- | --- | --- | --- | --- | --- | --- | --- | --- | --- | --- | --- | --- | --- | --- |
|  | **CHD+ (n = 13) vs CHD- (n = 12)** | | | | | | | | | | | | | | |
|  | ***Are the slopes equal?*** | | | | | | |  | ***Are the elevations or intercepts equal?*** | | | | | | |
|  | **F ratio** | **DFn** | **DFd** | **P-value (uncorrected)** | **Sig** | **pFDR** | **Sig** |  | **F ratio** | **DFn** | **DFd** | **P-value (uncorrected)** | **Sig** | **pFDR** | **Sig** |
| **Whole Brain Volumes** |  |  |  |  |  |  |  |  |  |  |  |  |  |  |  |
| ICV | 2.14 | 1 | 21 | 0.1584 | ns | 0.2977 | ns |  | 2.01 | 1 | 22 | 0.1705 | ns | 0.2243 | ns |
| TTV | 3.07 | 1 | 21 | 0.0945 | ns | 0.2646 | ns |  | 3.86 | 1 | 22 | 0.0621 | ns | 0.1915 | ns |
| **Main Tissue Volumes** |  |  |  |  |  |  |  |  |  |  |  |  |  |  |  |
| Cortical GM | 2.05 | 1 | 21 | 0.1670 | ns | 0.2977 | ns |  | 3.68 | 1 | 22 | 0.0680 | ns | 0.1915 | ns |
| Deep GM | 2.27 | 1 | 21 | 0.1470 | ns | 0.2977 | ns |  | 3.84 | 1 | 22 | 0.0628 | ns | 0.1915 | ns |
| WM | 3.27 | 1 | 21 | 0.0851 | ns | 0.2646 | ns |  | 2.84 | 1 | 22 | 0.1059 | ns | 0.1915 | ns |
| Cerebellum | 3.75 | 1 | 21 | 0.0664 | ns | 0.2646 | ns |  | 2.43 | 1 | 22 | 0.1331 | ns | 0.1915 | ns |
| Brainstem | 1.03 | 1 | 21 | 0.3208 | ns | 0.4251 | ns |  | 2.43 | 1 | 22 | 0.1334 | ns | 0.1915 | ns |
| eCSF | 0.36 | 1 | 21 | 0.5573 | ns | 0.5790 | ns |  | 0.01 | 1 | 22 | 0.9200 | ns | 0.9200 | ns |
| Lateral Ventricles | 0.05 | 1 | 21 | 0.8303 | ns | 0.8303 | ns |  | 2.84 | 1 | 22 | 0.1063 | ns | 0.1915 | ns |
|  |  |  |  |  |  |  |  |  |  |  |  |  |  |  |  |
| **Cortical GM segments** |  |  |  |  |  |  |  |  |  |  |  |  |  |  |  |
| Temporal Lobe GM | 2.02 | 1 | 21 | 0.1701 | ns | 0.2977 | ns |  | 6.84 | 1 | 22 | 0.0158 | * | 0.1915 | ns |
| Frontal Lobe GM | 1.19 | 1 | 21 | 0.2879 | ns | 0.4031 | ns |  | 1.61 | 1 | 22 | 0.2184 | ns | 0.2730 | ns |
| Parietal Lobe GM | 2.53 | 1 | 21 | 0.1269 | ns | 0.2961 | ns |  | 3.98 | 1 | 22 | 0.0585 | ns | 0.1915 | ns |
| Occipital Lobe GM | 3.13 | 1 | 21 | 0.0915 | ns | 0.2646 | ns |  | 2.61 | 1 | 22 | 0.1204 | ns | 0.1915 | ns |
| Cingulate Lobe GM | 0.45 | 1 | 21 | 0.5097 | ns | 0.5709 | ns |  | 3.36 | 1 | 22 | 0.0802 | ns | 0.1915 | ns |
| Insular Lobe GM | 0.98 | 1 | 21 | 0.3340 | ns | 0.4251 | ns |  | 2.61 | 1 | 22 | 0.1202 | ns | 0.1915 | ns |
|  |  |  |  |  |  |  |  |  |  |  |  |  |  |  |  |
| **WM segments** |  |  |  |  |  |  |  |  |  |  |  |  |  |  |  |
| Temporal Lobe WM | 2.63 | 1 | 21 | 0.1201 | ns | 0.2961 | ns |  | 4.93 | 1 | 22 | 0.0370 | * | 0.1915 | ns |
| Frontal Lobe WM | 0.79 | 1 | 21 | 0.3845 | ns | 0.4544 | ns |  | 1.01 | 1 | 22 | 0.3253 | ns | 0.3697 | ns |
| Parietal Lobe WM | 5.63 | 1 | 21 | 0.0273 | * | 0.2548 | ns |  | - | - | - | - | - | - | - |
| Occipital Lobe WM | 18.21 | 1 | 21 | 0.0003 | *** | 0.0084 | ** |  | - | - | - | - | - | - | - |
| Cingulate Lobe WM | 0.77 | 1 | 21 | 0.3895 | ns | 0.4544 | ns |  | 2.52 | 1 | 22 | 0.1265 | ns | 0.1915 | ns |
| Insular Lobe WM | 0.35 | 1 | 21 | 0.5583 | ns | 0.5790 | ns |  | 4.89 | 1 | 22 | 0.0378 | * | 0.1915 | ns |
|  |  |  |  |  |  |  |  |  |  |  |  |  |  |  |  |
| **Deep GM & Other** |  |  |  |  |  |  |  |  |  |  |  |  |  |  |  |
| Hippocampus | 1.73 | 1 | 21 | 0.2032 | ns | 0.3161 | ns |  | 2.37 | 1 | 22 | 0.1379 | ns | 0.1915 | ns |
| Amygdala | 3.32 | 1 | 21 | 0.0826 | ns | 0.2646 | ns |  | 1.08 | 1 | 22 | 0.3106 | ns | 0.3697 | ns |
| Caudate Nucleus | 4.10 | 1 | 21 | 0.0557 | ns | 0.2646 | ns |  | 8.09 | 1 | 22 | 0.0095 | ** | 0.1915 | ns |
| Lentiform Nucleus | 1.84 | 1 | 21 | 0.1895 | ns | 0.3121 | ns |  | 0.63 | 1 | 22 | 0.4360 | ns | 0.4542 | ns |
| Thalamus | 3.18 | 1 | 21 | 0.0892 | ns | 0.2646 | ns |  | 2.96 | 1 | 22 | 0.0992 | ns | 0.1915 | ns |

**Table S10:** Table of results for Spearman’s rank correlation tests for CHD+ and CHD- neonates with DS (using absolute volume z-scores).

Table of results for Spearman’s rank correlation tests assessing the correlation of absolute volume z-scores and PMA at scan (in weeks) for CHD+ and CHD- neonates with DS. All corresponding simple linear regression plots can be found in Figure S4. The uncorrected P-value, as well as the FDR-corrected P-value are shown. A cell highlighted in green indicates a P-value < 0.05. The R^2^ value is provided to assess linear model goodness of fit.

|  | ***Correlation of absolute z-scores and PMA at scan*** | | | | | | | | | | | |
| --- | --- | --- | --- | --- | --- | --- | --- | --- | --- | --- | --- | --- |
|  | **CHD+ (n = 13)** | | | | | | **CHD- (n = 12)** | | | | | |
|  | **Spearman's Rho** | **uncorrected P value** | **Sig** | **pFDR** | **Sig** | **R squared** | **Spearman's Rho** | **uncorrected P value** | **Sig** | **pFDR** | **Sig** | **R squared** |
| **Whole Brain Volumes** |  |  |  |  |  |  |  |  |  |  |  |  |
| ICV | -0.67 | 0.0149 | * | 0.0503 | ns | 0.48 | -0.40 | 0.1929 | ns | 0.6931 | ns | 0.15 |
| TTV | -0.73 | 0.0059 | ** | 0.0503 | ns | 0.48 | -0.18 | 0.5832 | ns | 0.8506 | ns | 0.05 |
|  |  |  |  |  |  |  |  |  |  |  |  |  |
| **Main Tissue Volumes** |  |  |  |  |  |  |  |  |  |  |  |  |
| Cortical GM | -0.69 | 0.0110 | * | 0.0503 | ns | 0.41 | -0.12 | 0.7111 | ns | 0.8932 | ns | 0.05 |
| Deep GM | -0.40 | 0.1706 | ns | 0.2063 | ns | 0.23 | 0.04 | 0.9076 | ns | 0.9602 | ns | 0.00 |
| WM | -0.63 | 0.0238 | * | 0.0663 | ns | 0.42 | -0.19 | 0.5601 | ns | 0.8506 | ns | 0.02 |
| Cerebellum | -0.71 | 0.0080 | ** | 0.0503 | ns | 0.48 | -0.43 | 0.1655 | ns | 0.6931 | ns | 0.26 |
| Brainstem | -0.39 | 0.1834 | ns | 0.2063 | ns | 0.22 | -0.37 | 0.2373 | ns | 0.6931 | ns | 0.20 |
| eCSF | -0.39 | 0.1834 | ns | 0.2063 | ns | 0.28 | -0.44 | 0.1581 | ns | 0.6931 | ns | 0.21 |
| Lateral Ventricles | -0.51 | 0.0781 | ns | 0.1172 | ns | 0.11 | -0.39 | 0.2145 | ns | 0.6931 | ns | 0.22 |
|  |  |  |  |  |  |  |  |  |  |  |  |  |
| **Cortical GM segments** |  |  |  |  |  |  |  |  |  |  |  |  |
| Temporal Lobe GM | -0.54 | 0.0615 | ns | 0.1107 | ns | 0.34 | 0.19 | 0.5450 | ns | 0.8506 | ns | 0.00 |
| Frontal Lobe GM | -0.62 | 0.0261 | * | 0.0663 | ns | 0.37 | -0.27 | 0.3926 | ns | 0.8154 | ns | 0.14 |
| Parietal Lobe GM | -0.59 | 0.0351 | * | 0.0752 | ns | 0.40 | 0.06 | 0.8470 | ns | 0.9529 | ns | 0.01 |
| Occipital Lobe GM | -0.67 | 0.0138 | * | 0.0503 | ns | 0.49 | -0.32 | 0.3150 | ns | 0.7603 | ns | 0.13 |
| Cingulate Lobe GM | -0.52 | 0.0711 | ns | 0.1129 | ns | 0.20 | -0.55 | 0.0687 | ns | 0.6931 | ns | 0.24 |
| Insular Lobe GM | -0.22 | 0.4614 | ns | 0.4614 | ns | 0.09 | 0.45 | 0.1406 | ns | 0.6931 | ns | 0.00 |
|  |  |  |  |  |  |  |  |  |  |  |  |  |
| **WM segments** |  |  |  |  |  |  |  |  |  |  |  |  |
| Temporal Lobe WM | -0.55 | 0.0557 | ns | 0.1074 | ns | 0.38 | 0.02 | 0.9602 | ns | 0.9602 | ns | 0.00 |
| Frontal Lobe WM | -0.41 | 0.1645 | ns | 0.2063 | ns | 0.21 | -0.44 | 0.1581 | ns | 0.6931 | ns | 0.14 |
| Parietal Lobe WM | -0.69 | 0.0119 | * | 0.0503 | ns | 0.44 | 0.11 | 0.7278 | ns | 0.8932 | ns | 0.01 |
| Occipital Lobe WM | -0.89 | <0.0001 | **** | 0.0027 | ** | 0.78 | 0.15 | 0.6301 | ns | 0.8506 | ns | 0.05 |
| Cingulate Lobe WM | -0.46 | 0.1110 | ns | 0.1499 | ns | 0.12 | -0.02 | 0.9426 | ns | 0.9602 | ns | 0.02 |
| Insular Lobe WM | -0.28 | 0.3456 | ns | 0.3589 | ns | 0.10 | -0.30 | 0.3379 | ns | 0.7603 | ns | 0.10 |
|  |  |  |  |  |  |  |  |  |  |  |  |  |
| **Deep GM & Other** |  |  |  |  |  |  |  |  |  |  |  |  |
| Hippocampus | -0.62 | 0.0270 | * | 0.0663 | ns | 0.46 | -0.35 | 0.2567 | ns | 0.6931 | ns | 0.17 |
| Amygdala | -0.50 | 0.0873 | ns | 0.1241 | ns | 0.34 | 0.24 | 0.4588 | ns | 0.8506 | ns | 0.01 |
| Caudate Nucleus | -0.52 | 0.0711 | ns | 0.1129 | ns | 0.32 | -0.20 | 0.5303 | ns | 0.8506 | ns | 0.00 |
| Lentiform Nucleus | -0.37 | 0.2180 | ns | 0.2354 | ns | 0.12 | 0.17 | 0.5987 | ns | 0.8506 | ns | 0.04 |
| Thalamus | -0.59 | 0.0362 | * | 0.0752 | ns | 0.35 | -0.09 | 0.7696 | ns | 0.9034 | ns | 0.03 |
